# Large-scale screening of HBV epitopes restricted by multiple prevalent HLA-B/C allotypes and routine detection of HBV-specific T cells in CHB patients

**DOI:** 10.3389/fimmu.2025.1717543

**Published:** 2026-01-09

**Authors:** Yandan Wu, Yu Zhao, Ruixue Ji, Pinqing Li, Huijuan Chen, Fangping Yue, Yi Wu, Jie Qiu, Chuanlai Shen

**Affiliations:** 1 Department of Microbiology and Immunology, Medical School of Southeast University, Nanjing, Jiangsu, China; 2 Department of Hepatitis, The Second Hospital of Nanjing, Affiliated to Nanjing University of Chinese Medicine, Nanjing, Jiangsu, China; 3 Department of Infectious Diseases, The First Affiliated Hospital of Bengbu Medical University, Bengbu, Anhui, China

**Keywords:** ELISpot assay, hepatitis B virus, HLA-A, HLA-B, HLA-C, T-cell epitope

## Abstract

**Background and aims:**

In our previous work, a total of 103 CD8^+^ T-cell epitopes restricted by 13 prevalent HLA-A allotypes in Northeast Asians were validated from the main proteins of hepatitis B virus (HBV). This study aims to further screen the T-cell epitopes restricted by 15 prevalent HLA-B and 14 prevalent HLA-C allotypes and establish a universal assay for counting reactive HBV-specific T cells in patients with chronic hepatitis B (CHB).

**Methods:**

CD8^+^ T-cell epitopes were systematically screened through a combination of *in silico* prediction, *ex vivo* co-cultures of peptides with patient-derived peripheral blood mononuclear cells (PBMCs), and peptide competitive binding assays using HLA-B/C transfected cell lines.

**Results:**

A total of 89 novel CD8^+^ T-cell epitopes were identified from four HBV main proteins using PBMCs from 250 CHB patients. Furthermore, 201 validated CD8^+^ T-cell epitope peptides restricted by the 13 HLA-A, 15 HLA-B, and 14 HLA-C allotypes were integrated to construct a broad-spectrum epitope peptide library, and by which the ELISpot assay was established followed by clinical testing for 81 CHB patients. The counts of reactive HBV-specific T cells and T cells reactive to each HBV protein (HBsAg-, HBpol-, HBx-, or HBeAg-) in PBMCs showed a negative correlation with serum HBsAg levels and no correlation with HBeAg or ALT levels. NUCs/IFN-α combination elicited significantly more reactive HBV-specific T cells (including those targeting HBsAg, HBpol, HBx, or HBeAg) than NUCs or IFN-α monotherapy.

**Conclusions:**

The proposed method holds great potential for facilitating routine evaluation of HBV-specific CD8^+^ T cell reactivity in CHB patients.

## 1 Introduction

Hepatitis B virus (HBV) infection affects over 250 million people globally, with an estimated 880,000 deaths annually attributed to liver cirrhosis (LC) and hepatocellular carcinoma (1, 2). HBV-specific T cells significantly influence the outcome of HBV infection (3–5), antiviral efficacy (6) and disease recurrence after therapy discontinuation (7–9). Especially, HBV-specific CD8^+^ T cell-mediated cytotoxicity is crucial for viral clearance, as numerous viral T-cell epitope peptides can be presented to CD8^+^ T cells via HLA-A, B, C molecules, enabling cytotoxic T lymphocytes to kill virus-infected hepatocytes. To date, the functionally validated T-cell epitopes of HBV proteome remains very limited, including only 205 CD8^+^ T-cell epitopes and 79 CD4^+^ T-cell epitopes as our previously reviewed, and most ones are presented by a few common HLA supertypes such as HLA-A0201, A2402, DR04, and DR12 molecules, while little is known about the epitopes presented by prevalent HLA-B and C allotypes (10). However, HLA-B and C allotypes, as classical HLA class I molecules, can also present antigenic peptides and induce robust CTL responses. Moreover, certain HLA-B or -C allotype carriers are correlated with the responsiveness to IFN-α therapy and can predict the outcome of HBV infection (11–13). Therefore, the currently validated T-cell epitope profile of HBV cannot cover the highly polymorphic HLA alleles in a designated geographical population and can not represent the richness of epitopes in each HBV protein. This limitation has significantly hindered the development of detection systems for universally enumerating HBV-specific T cells, particularly CD8^+^ T cell reactivity, and also impeded the development of therapeutic vaccines based on T-cell epitope peptides.

In our previous researches (14, 15), 103 CD8^+^ T-cell epitopes were defined from HBsAg (covering pre-S1, pre-S2 and S), HBeAg (containing HBcAg), HBx, and HBpol proteins. They are restricted by 13 prevalent HLA-A allotypes which cover over 90% of Northeast Asians, even higher in Chinese population. Using this broad-spectrum CD8^+^ T-cell epitope peptides, an ELISpot assay was set up to count reactive HBV-specific CD8^+^ T cells for patients with chronic hepatitis B (CHB). In order to enhance the universality of this detection tool for a broader range of patients, this study focuses on the large-scale screening of the T-cell epitopes restricted by prevalent 15 HLA-B and 14 HLA-C allotypes which gathering, respectively, an accumulative gene frequency of over 70% and 90% in Northeast Asian population, 73% and 94% in Chinese population. Consequently, these validated CD8^+^ T-cell epitopes, restricted by these prevalent HLA-A, B and C allotypes, were integrated to improve the previous ELISpot assay, followed by methodological validation and clinical testing in CHB patients.

## 2 Methods

### 2.1 Patient cohort and HLA genotyping

This study recruited 331 HBV-infected CHB patients and 10 HBV-infected LC patients from the Department of Hepatitis, the Second Hospital of Nanjing. According to the EASL 2017 Clinical Practice Guidelines on the management of hepatitis B virus infection (9), CHB patients exhibit clinical, biochemical, and virological evidences of chronic hepatitis B infection with positive HBsAg for at least 6 months; the LC patients were also defined by liver histology or clinical, laboratory and imaging evidences. Exclusion criteria for these subjects include infection with hepatitis C virus, hepatitis A virus, or human immunodeficiency virus, as well as malignancies. Clinical and laboratory data were collected from patient’ electronic medical records. For each patient, the written informed consent was obtained. Peripheral blood sample collection and utilization in this study were conducted in accordance with the principles outlined in the Helsinki Declaration and have been approved by the Clinical Ethics Committee of the Second Hospital of Nanjing (2018-LY-kt054, 2019-LY-ky011, 2021-LS-ky013).

Genomic DNA was extracted from each fresh blood sample using the Blood DNA Extraction kit from Tiangen Biotech Co., Ltd (Beijing) and followed by PCR-sequencing-based typing on HLA-B or C loci (exon 2 and 3) using the primers recommended by International HLA working group (IHWG).

### 2.2 *In silico* prediction of CD8^+^ T-cell epitopes and peptide synthesis

IEDB online epitope prediction tool (http://tools.iedb.org/main/), including seven algorithms (NetMHCpan 4.1 BA, NetMHCpan 4.1 EL, Pickpocket, NetMHCstabpan, ANN, SMM and consensus) was used to *in silico* predict T-cell epitopes spanning HBsAg (covering pre-S1, pre-S2 and S), HBeAg (containing HBcAg), HBpol, and HBx proteins and presented by different HLA-B and HLA-C allotypes. For each protein and each HLA-B or HLA-C allotype, 1 to 10 top-scoring (highest affinity) 9-mer and 10-mer peptides as defined by at least two prediction tools were selected as candidate epitopes for further validation. The gene frequency data for each HLA-B and HLA-C allele in the Northeast Asian and Chinese population were obtained from the international online database (https://allelefrequencies.net) and a number of published regional literature works. After comprehensive analysis, 15 HLA-B and 14 HLA-C alleles with a gene frequency greater than 1% in the Chinese population were selected, which together account for approximately 70% and 90% of the gene frequencies, respectively, in Northeast Asian and Chinese populations. The 15 HLA-B allotypes include B4601, B4001, B5801, B1502, B5101, B1301, B1302, B1501, B4006, B5401, B3501, B5201, B0702, B4403, B4801, and the 14 HLA-C allotypes include C0102, C0602, C0702, C0801, C0304, C0302, C0303, C0401, C1402, C1202, C1502, C1403, C1203, C0701. This study primarily focused on the common HBV serotypes (adr and adw) as well as prevalent genotypes B and C in the Northeast Asian populations, with consideration for genotypes A and D. The entire amino acid sequences of each protein from different genotypes were obtained from the UniProt database and aligned as shown in **Supplementary Figure S1**. Peptides were synthesized by GenSript Biotech Corporation (Nanjing, China), with purity >95%. Freeze-dried peptides were reconstituted in dimethyl sulfoxide (DMSO) and serum-free medium (Dakewe Biotech Co., Ltd., Shanghai) solution at a stock concentration of 2 mg/mL and stored in aliquots at -80 °C.

### 2.3 Peptide-PBMCs coculture experiment using patient-derived PBMCs

Briefly, fresh peripheral blood (20 mL) with heparin was collected from each patient, and peripheral blood mononuclear cells (PBMCs) were immediately separated by density gradient centrifugation using Human Lymphocyte Separation Medium (Dakewe Biotech) and seeded in 96-well plates (4×10^5^ PBMCs/well), and then co-cultured with a single candidate epitope peptide (40 μg/mL), that presented by the patient’s HLA allotypes as *in silico* predicted, in RPMI1640 medium with 10% FBS (BioChannel Biological Technology, Nanjing, China) at 37 °C, 5% CO_2_ for 1 hour. In parallel, the PBMCs co-culture well with PHA (10 μg/mL) and PBMCs alone well were also carried out as positive control and negative control, respectively. Subsequently, a mixture of Brefeldin A and Monensin (420601, 420701, Biolegend, CA, US) was added to each well followed by an additional 5-hour co-culture. Cells were then harvested, washed, and blocked with Fc receptor-blocking reagent (130-059-901, Miltenyi Biotec, German) for 20 minutes, followed by staining with FITC-conjugated anti-human CD3 (UCHT1 clone, Biolegend) and APC-conjugated anti-human CD8 (SK1 clone, Biolegend) at 4°C for 30 minutes. The cells did not stain with viability dye. After washing, the cells were fixed and permeabilized using the Fix & Perm kit (Multi Sciences Biotech Co., Ltd., Hangzhou, China) according to the protocol, stained with PE-conjugated anti-human IFN-γ antibody (B27 clone, BD Bioscience, NJ, US) at 4°C for further 30 minutes, and then analyzed by flow cytometry (Guava^®^ easyCyteTM HT, Luminex Corporation, Austin, TX, US). The frequency of IFN-γ^+^ cells in the CD3^+^/CD8^+^ population in each well was calculated.

### 2.4 Peptide competitive binding assay for HLA-B and HLA-C molecules using transfected HMy2.CIR cell lines

HMy2.CIR is a human B lymphocyte strain with HLA class I antigen deficiency, which does not express HLA-A and B molecules and only expresses trace HLA-Cw4. The HMy2.CIR cell lines stably expressing indicated HLA-B molecules (B4601, B4001, B5801, B1502, B5101, B1301, B1302, B1501, B4006, B5401, B3501, B5201, B0702, B4403, and B4801), or HLA-C molecules (C0102, C0602, C0702, C0801, C0304, C0302, C0303, C0401, C1402, C1202, C1502, C1403, C1203, and C0701) were constructed in our prior research (16). Peptide competitive binding assays were performed as previously described (14, 16, 17). A set of Cy5-labeled reference peptides for the indicated HLA-B and C molecules (**Supplementary Table S1**).

Briefly, HMy2.CIR cells stably expressing specific HLA-B or C molecules were incubated with Cy5-labeled reference peptides (either from published papers or in-house validated) and no-labeled candidate epitope peptide (tested peptide). In parallel, a maximum control well (CIR cells and Cy5-reference peptide) and a background well (CIR cells only) were set up. After a 24-hour incubation period, unbound peptides were removed. The relative binding affinity of the tested peptide to the indicated HLA-B or C molecule was quantified by the decrease in the mean fluorescence intensity (MFI) of the reference peptide binding to the CIR cell surface. Each cell line was simultaneously subjected to a series of peptide competitive binding assays for testing more than ten epitope peptides to avoid batch-to-batch errors in different assay batches. Notably, each HMy2.CIR cell line was treated with an acidic buffer solution, leveraging a low pH environment to disrupt the binding affinity between HLA class I molecules and endogenous peptides, thereby facilitating the release of endogenous peptides from the antigen-binding groove. Recombinant human β2-microglobulin protein was then supplemented into the culture medium, followed by the co-incubation of Cy5-reference peptide and unlabeled candidate epitope peptide. The relative binding affinity of a given peptide was calculated using the formula: Competitive binding (%) = [1–(MFI_sample_– MFI_background_)/(MFI_max_–MFI_background_)]×100%. The concentration of unlabeled tested peptide needed to inhibit the binding of the Cy5-labeled reference peptide by 50% (IC_50_) was calculated from the competitive binding inhibition (%) of the tested peptide at 5 and 15 μM. The binding affinity of each unlabeled peptide with the indicated HLA-B or C molecule was assessed according to the IC_50_ value. IC_50_ < 5 μM (5 μM inhibition >50%) means high binding affinity, 5 μM< IC_50_ < 15 μM (5 μM inhibition <50% but 15 μM inhibition > 50%) means intermediate binding affinity, IC_50_ > 15 μM means low or no binding affinity (5 μM inhibition 20% -50% or 15 μM inhibition 30% -50% means low binding affinity; 5 μM inhibition < 20% or 15 μM inhibition < 30% means no binding affinity.

### 2.5 ELISpot assay to count reactive HBV-specific T cells

Fresh anticoagulated peripheral blood (7 mL) was collected from each patient. PBMCs were isolated by density gradient centrifugation, and were adjusted to 4 × 10^6^ cells/mL in serum-free cell culture medium (Dakewe Biotech), seeded into 13 wells in 96-well ELISpot plate pre-coated with human IFN-γ ELISpot capture antibody (551873, 1:200 dilution, BD Pharmingen, San Diego, CA, USA) at a density of 4 × 10^5^ cells/well (but only 2 × 10^5^ cells in the positive control well), and then 11 peptide pools were added into the 1^th^ to 11^th^ experimental wells, with one well dedicated to one peptide pool (27 μL/peptide pool/well, 2 μg/peptide/well). Simultaneously, the irreverent peptide (myelin-oligodendrocyte glycoprotein, MOG_37-46_, 5μg/well) was added into the 12^th^ well as a negative control, and phytohemagglutinin (PHA, 2.5 μg/well) was added into the 13^th^ well as a positive control. DMSO and dimethylformamide (DMF) were added to the negative control well to equalize the DMSO/DMF concentration with that in the peptide/PBMCs co-culture wells. The plate was incubated in a cell culture incubator for 20 h. Then, wells were washed with deionized water to remove the cells, human IFN-γ detection antibody (BD Pharmingen, 551873, diluted 1:250) was added to each well and incubated at 37 °C for 2 h. After washes, HRP-streptavidin (557630, diluted 1:100, BD Pharmingen) was added into each well and incubated at 37 °C for another 1 h. After washing, AEC set (551951, diluted 1:50, BD Pharmingen) was added into each well to develop the color spots for 30 min, and followed by air-dry and spots counting. For each blood sample, subtract the number of spots in the negative control well from the number of spots in each experimental well, then sum up these values to obtain the actual total number of spots (spot-forming units, SFUs) in the 11 experimental wells. This represents the count of HBV-specific T cells with reactivity in 4×10^5^ PBMCs. When the spot number in the indicated experimental well is less than that in the negative control well, SFUs in the indicated experimental well are taken as 0 SFUs. Meanwhile, the SFUs/4×10^5^ PBMCs for each HBV protein were also calculated according to the peptide pools derived from each protein. The clinical baseline features and the real-time data from the clinical laboratory were collected for each patient.

### 2.6 Statistical analysis

Statistical analysis was performed using GraphPad Prism 9 (GraphPad, La Jolla, CA, USA). Data were presented as median (interquartile range). A Mann-Whitney (non-parametric) test was used for the analyses of SFUs medians between two groups. Kruskal-Wallis test (non-parametric) was performed when analyzing more than two groups. Spearman correlation test was performed to analyze correlation between continuous data. Multivariate linear regression analysis was performed to explore the factors effecting HBV-specific T cells (SFUs). The diagnostic performances of HBV-specific T cells (SFUs) for the discrimination of CHB and LC were assessed by receiver operating characteristic (ROC) and area under the ROC curve (AUC). P < 0.05 was considered statistically significant.

## 3 Results

### 3.1 96 CD8^+^ T-cell candidate epitopes restricted by 15 HLA-B allotypes and 89 CD8^+^ T-cell candidate epitopes restricted by 14 HLA-C allotypes were *in silico* predicted from HBV main antigens

Ultimately, 256 and 276 T-cell epitopes binding to HLA-B and -C allotypes were chosen as candidate epitopes, respectively. After removing sequence-identical repetitive candidates cross-bound by different HLA-B or C molecules, only 96 and 89 sequence-distinct candidate epitopes restricted by HLA-B (**Table 1**) and -C (**Table 2**) molecules were synthesized as peptides for further validation. Of which, 24, 38, 13, 21 candidates binding to HLA-B (**Supplementary Table S2**; **Supplementary Figure S2A**) and 24, 34, 14, 17 candidates binding to HLA-C (**Supplementary Table S3**; **Supplementary Figure S2B**) harbor in HBsAg, HBeAg, HBx, and HBpol proteins, respectively, with a similar distribution density of epitopes in each protein.

**Table 1:** 57 of 96 candidate epitopes restricted by HLA-B molecules were validated by peptide-PBMC *ex vivo* coculture experiments and their HLA cross-restrictions.

Peptide ID	Peptide name	Peptide sequence	HLA-B allotypes binding to epitopes as *in silico* predicted	HLA-B allotypes of the patients displaying positive CD8^+^ T cell response in peptide-PBMCs cocultures	HLA-B allotypes binding to epitopes in competitive peptide binding assay			
					High affinity	Inter affinity	Low affinity	No affinity
T8	HBe_139-147_	FGRETVLEY	B4601/1502/1501		B1501	B1502	B4601	
T13	HBe_38-47_	FGASVELLSF	B4601			B4601		
**T35**	HBe_105-113_	LEDPASREL	B4001/1301/4006	2 patients B4001/1301/1302	B4001		B4006>B1301>B1302	
**T36**	HBe _141-149_	RETVLEYLV	B4001/1302/4006	1 patient B1302			B4001>B4006>B1302	
**T38**	HBe_68-76_	REALESPEH	B4006	1 patient B1501/4801			B1501>B4006	
T39	HBe_111-120_	RELVVSYVNV	B4001/1302/4006/1501		B1501>B4001		B1302>B4006	
T40	HBe_92-101_	GELMNLATWV	B5801/1301/4006		B5801		B1301	B4006
T69	HBe_18-26_	QASKLCLGW	B1301/5801				B5801>B1301	
T70	HBe_45-53_	LSFLPSDFF	B1502/5801/1501		B1501	B1502	B5801	
**T71**	HBe_122-131_	MGLKIRQLLW	B5801	1 patient B5801	B5801			
**T72**	HBe_115-124_	VSYVNVNMGL	B5801	2 patients B1501/4801/5801			B5801>B1501	
**T100**	HBe_131-139_	WFHISCLTF	B1502	1 patient B1301/4601	B4601>B1502	B1301		
**T101**	HBe_26-35_	WLRGMDIDTY	B1502	2 patients B4601/1522/5102/5111			B1502	B4601
**T124**	HBe_1-9_	MQLFHLCLI	B5101/1301/1302	1 patient B1301/4601	B1301	B5101	B4601>B1302	
**T125**	HBe_107-115_	DPASRELVV	B5101/5401/4601/1501	1 patient B1501/5601			B5101>B1501	B4601>B5401
**T126**	HBe_14-22_	CPTVQASKL	B5101	2 patients B1501/5101/5102/5111	B5101		B1501	B4601
**T127**	HBe_48-56_	LPSDFFPSI	B5101	3 patients B5101/4001/B1501		B5101	B1501>B4001	
**T182**	HBe_127-135_	RQLLWFHIS	B1302/1502	3 patients B1302/1502/1302/1501	B1501		B1502>B1302	
T270	HBe_184-192_	SPRRRTPSP	B5401		B5401			
**T272**	HBe_78-87_	SPHHTALRQA	B5401	1 patient B5102/4801				B5401
T273	HBe_166-175_	APILSTLPET	B5401				B5401	
**T19**	Pol_541-549_	RAFPHCLAF	B4601/5801/1301/1501/1502/5101/5401	1 patient B1502/1302	B1502>B5801		B1301>B5101>B1302>B1501	B4601>B5401
**T20**	Pol_409-417_	FAVPNLQSL	B4601	1 patient B4601/1522				B4601
T21	Pol_428-436_	LSLDVSAAF	B1502/4601/5801/1501		B1501>B4601	B5801> B1502		
T24	Pol_834-842_	FASPLHVAW	B4601/5801		B4601	B5801		
T25	Pol_644-653_	FAAPFTQCGY	B4601/5401				B5401>B4601	
**T47**	Pol_529-538_	AQFTSAICSV	B4001/1301/1302/4006	1 patient B1301/4601			B4001>B1302>B4006>B1301	B4601
**T48**	Pol_40-48_	AEDLNLGNL	B4001	1 patient B4001			B4001	
**T50**	Pol_162-170_	RETTRSASF	B4001/4006	1 patient B1501/Bw57	B4001		B1501>B4006	
T52	Pol_175-184_	WEQELQHGRL	B4001/4006				B4001>B4006	
**T54**	Pol_384-392_	TESRLVVDF	B4001	1 patient B4001/5101	B4001		B5101	
T55	Pol_16-25_	DEAGPLEEEL	B4001			B4001		
T57	Pol_331-339_	SEPCSDYCL	B4001				B4001	
T59	Pol_679-687_	KQYLHLYPV	B4001/4006		B4001	B4006		
T61	Pol_5-14_	YQHFRKLLLL	B4001/1301				B1301>B4001	
**T84**	Pol_560-568_	KSVQHLESL	B5801	2 patientsB1501/4801/5801	B5801		B1501	
**T86**	Pol_65-74_	STVPVFNPEW	B5801	2 patients B1501/4801/5801	B1501>B5801			
**T110**	Pol_640-648_	GLLGFAAPF	B1502/1501	1 patient B1301/4601	B4601>B1501		B1502>B1301	
T111	Pol_772-781_	WILRGTSFVY	B1502/1501		B1501	B1502		
**T114**	Pol_676-685_	FTFSPTYKAF	B1502	1 patient B5102/5111	B1502			
**T115**	Pol_667-675_	QAFTFSPTY	B1502	2 patients B1502/1302/4601/1522			B1302>B1502	B4601
**T136**	Pol_760-768_	FPWLLGCAA	B5101/5401	1 patient B5101	B5101			B5401
**T141**	Pol_440-449_	HPAAMPHLLV	B5101/5401	1 patient B1301/4601	B4601>B5101		B1301	B5401
T143	Pol_547-556_	LAFSYMDDVV	B5101					B5101
**T144**	Pol_111-119_	MPARFYPNL	B5101	1 patient B5102/5111	B5101			
**T165**	Pol_502-510_	KLHLYSHPI	B1301/1302	1 patient B4601	B4601		B1301>B1302	
**T166**	Pol_389-397_	RLVVDFSQF	B1301/1501	1 patient B1501/5601	B1502>B1501		B1301	
T167	Pol_594-602_	SLNFMGYVI	B1302				B1302	
**T170**	Pol_661-669_	IQSKQAFTF	B1301	1 patient B5102/5111		B1301		
**T195**	Pol_370-378_	RVTGGVFLV	B1302	1 patient B4001			B1302>B4001	
**T199**	Pol_93-101_	QQYVGPLTV	B1302	1 patient B4601	B4601		B1302	
T201	Pol_74-82_	WQTPSFPNI	B1302				B1302	
T203	Pol_393-402_	SQFSRGSTHV	B1302				B1302	
T228	Pol_633-642_	CQRIVGLLGF	B1501		B1501			
**T248**	Pol_730-738_	AELLAACFA	B4601/4006	1 patient B5102/5111			B4601>B4006	
**T252**	Pol_353-362_	TEHGEHNIRI	B4006	1 patient B4001	B4006		B4001	
T257	Pol_23-31_	LEEELPRLA	B4006			B4006		
**T260**	Pol_302-311_	VELHNIPPSC	B4006	1 patient B5102/4801	B4006			
T280	Pol_444-452_	MPHLLVGSS	B5401			B5401		
**T1**	HBs_119-127_	AMQWNSTTF	B4601/1502/1301/1501	1 patient B1301/4601	B1301>B4601		B1501>B1502	
**T2**	HBs_16-24_	LSVPNPLGF	B4601/5801	1 patient B5801/3501	B5801>B4601			
T3	HBs_344-353_	FSWLSLLVPF	B4601				B4601	
T27	HBs_252-261_	RRFIIFLFIL	B4001				B4001	
**T29**	HBs_274-283_	YQGMLPVCPL	B4001	1 patient B1301/4601	B4601		B4001>B1301	
T33	HBs_380-389_	YNILSPFLPL	B4001/B4601/1502		B4601>B1502>B4001			
T64	HBs_321-330_	CTCIPIPSSW	B5801				B5801	
**T90**	HBs^371-379^	MMWYWGPSL	B1502	1 patient B1502/1302	B1502		B1302	
**T116**	HBs_390-398_	LPIFFCLWV	B5101/5401	1 patient B5101			B5101	B5401
**T119**	HBs_361-369_	SPTVWLSVI	B5101/5801	2 patients B5101/1501/4001	B5801		B5101>B1501>B4001	
**T120**	HBs_351-360_	VPFVQWFAGL	B5101	3 patients B5101/1501/5102/4801			B5101>B1501	
**T122**	HBs_387-396_	LPLLPIFFCL	B5101	3 patients B5101/1501/4001	B5101		B4001	
**T146**	HBs_99-108_	RQSGRQPTPI	B1301/1302	1 patient B4601		B1301	B4601>B1302	
**T150**	HBs_175-183_	MENTTSGFL	B1301	2 patients B5401/4601/1522	B5401		B1301	B4601
**T176**	HBs_272-280_	LDYQGMLPV	B1302/4006/4001/1301/1501	1 patient B1501/5601			B4001>B1302>B1501>B4006>B1301	
**T177**	HBs_248-256_	WMCLRRFII	B1302	1 patient B5101			B1302>B5101	
T206	HBs_349-357_	LLVPFVQWF	B5801/1501		B1501			B5801
**T207**	HBs_186-194_	LLVLQAGFF	B1501	3 patients B1501/5601/4801/4601/1511			B1501	B4601
T231	HBs_28-36_	HQLDPAFGA	B4006		B4006			
T233	HBs_81-90_	AQGILTTVPA	B4006		B4006			
T261	HBs_25-33_	FPDHQLDPA	B5401	1 patient B5401		B5401		
**T262**	HBs_324-333_	IPIPSSWAFA	B1502/5101/5401	1 patient B4601		B1502	B5101	B4601>B5401
T263	HBs_46-55_	NPNKDHWPEA	B5401			B5401		
T267	HBs_308-317_	FPSCCCTKPS	B5401		B5401			
**T14**	HBx_63-71_	FSSAGPCAL	B5101/4601/4001/5801/1502/1301/1501	1 patient B4001/5101	B5801		B1502>B1301>B5101>B4601>B1501	B4001
T16	HBx_102-111_	AMSTTDLEAY	B1501/4601/5801/1502				B1502>B1301	B5801>B1501>B4601
**T18**	HBx_54-63_	SLRGLPVCAF	B1302/4601/1502/1501	2 patients B4601/1301/4601		B1502	B1301>B1501>B1302	B4601
**T44**	HBx_120-129_	WEELGEEIRL	B4001/4006/1301	1 patient B4001	B4001		B4006>B1301	
**T45**	HBx_125-134_	EEIRLMVFVL	B1302/4006/4001	3 patients B1302/4001/5502			B4001>B1302>B4006	
**T130**	HBx_10-18_	DPARDVLCL	B5101	1 patient B1501/5101	B1501		B5101	
T132	HBx_89-98_	LPKVLHKRTL	B5101				B5101	
T134	HBx_1-9_	MAARVCCQL	B5101/1301				B1301>B5101	
T160	HBx_36-45_	KEFGASVELL	B1301				B1301	
T162	HBx_7-16_	CQLDPARDVL	B1301/1302/4006				B4006>B1302	B1301
**T186**	HBx_13-21_	RDVLCLRPV	B1302/4006	1 patient B4601	B4601		B1302>B4006	
**T220**	HBx_141-150_	LVCSPAPCNF	B1501	1 patient B1501/5601	B1501			
T275	HBx_146-154_	APCNFFTSA	B5401			B5401		

Each validated epitope peptide (VEP) was bolded.

**Table 2:** 32 of 89 candidate epitopes restricted by HLA-C molecules were validated by peptide-PBMC *ex vivo* coculture experiments and their HLA cross-restrictions.

PeptideID	Peptide name	Peptide sequence	HLA-C allotypes binding to epitopes as *in silico* predicted	HLA-C allotypes of the patients displaying positive CD8^+^ T cell response in peptide-PBMCs cocultures	HLA-C allotypes binding to epitopes in competitive peptide binding assay			
					High affinity	Inter affinity	Low affinity	No affinity
W1	HBs_77-85_	FLPSDFFPSI	C0102/0401			C0102		C0401
W2	HBs_16-24_	FFPSIRDLL	C0102/0702/0401/0602/1402/1403		C1403>C0102	C0602	C1402>C0401>C0702	
**W5**	HBs_339-347_	HCSPHHTAL	C0102/0302/0303/0304/0702/0801	1 patient C0102/0702	C0102	C0302>C0801	C0304>C0702>C0303	
**W6**	HBs_118-127_	AYRPPNAPIL	C0102/0702/1402/1403/0401/0602/0701	1 patient C0701/0702	C0701	C0602>C0102>C0702	C1403>C1402>C0401	
W10	HBs_324-332_	RAFPHCLAF	C0702/0801/0304/0302/0303/0401/1202/1502/1403/1203/0102		C0302>C0102>C1502>C1403		C1202>C0801>C0304>C0303> C1203>C0401>C0702	
W38	HBs_234-242_	YRWMCLRRF	C0602/0702/0701		C0701>C0702			C0602
**W74**	HBs_299-307_	STLPETTVV	C0303/0801/1203	1 patient C0303	C0303	C1203>C0801		
**W92**	HBs_371-379_	ASALYREAL	C0303/0801/0304	1 patient C0304/0401		C0401		C0801>C0303
W94	HBs_378-386_	QAILCWGEL	C0304		C0304			
W125	HBs_112-120_	FAAPFTQCGY	C0302/1202/1203			C1203	C1202>C0302	
W162	HBs_246-254_	LLDTASALY	C0401				C0401	
W163	HBs_252-260_	LEDPASREL	C0401				C0401	
**W114**	HBs_342-350_	FGASVELLSF	C0302/1202/0303/0304/0801	1 patient C0801/1402	C1402>C0304	C1202>C0302	C0801>C0303	
**W117**	HBs_157-165_	ASRELVVSY	C0302/0701/1203/0602	1 patient C0602	C0602	C1203	C0701>C0302	
W185	HBs_123-131_	WFHISCLTF	C1402/1403				C1402>C1403	
W210	HBs_205-213_	TVLEYLVSF	C0302/1202				C0302	C1202
W230	HBs_131-139_	HTALRQAIL	C0102				C0102	
**W7**	HBs_336-344_	FAVPNLQSL	C0102/0602/0801/1202/0303	1 patient C0303	C0102	C0602>C1202	C0801>C0303	
**W8**	HBs_382-390_	YHIPLHPAAM	C0102/1402/0701/0302	1 patient C0701/0702	C0102>C0701	C0302	C0702>C1402	
W11	HBs_208-216_	FSYMDDVVL	C0801/0304/0302/0303/1202/1203 0102			C0302>C1203>C0102	C0304>C1202>C0801>C0303	
W12	HBs_146-154_	CGYPALMPL	C0102				C0102	
W14	HBs_328-336_	PLPIHTAEL	C0102			C0102		
W15	HBs_80-88_	FTQCGYPAL	C0304/0303/0401/0102/0801		C0303	C0304	C0102>C0801>C0401	
**W26**	HBs_197-206_	SYVNVNMGL	C0401/0801/0602/0702/1402/1403	5 patients C0702/0401/0801/1402	C0602			C1403
W30	HBe_47-56_	LSYQHFRKL	C0602/1502/1402/1403		C0602	C1502	C1402>C1403	
W31	HBe_52-60_	FYPNLTKYL	C0602/1403/0702/0401/1402		C1402>C0602		C0401>C0702>C1403	
W32	HBe_137-145_	FRNSEPCSEY	C0701			C0701		
W34	HBe_76-84_	SRNLYVSLL	C0602/0702/0701		C0602		C0701>C0702	
W36	HBe_160-169_	VRRAFPHCL	C0602/0701/0702			C0602	C0701>C0702	
W52	HBe_116-124_	LYSSTVPVF	C0702/0401/1402/1403		C1402		C0401>C1403	C0702
W57	HBe_97-105_	KYTSFPWLL	C0702					C0702
**W71**	HBe_170-178_	LATWVGSNL	C0302/0303/0304/0801/1202/1203	1 patient C0303	C0303>C0304	C1203	C1202>C0302>C0801	
**W75**	HBe_63-71_	SSSGHAVEL	C0801/0304/0303/1502	1 patient C0303	C0303		C0801>C0304>C1502	
**W77**	HBe_86-94_	SAAFYHIPL	C0801/0304/0303	1 patient C0303	C0304		C0801>C0303	
W78	HBe_38-47_	FTSAICSVV	C0801/1202/1502		C1502	C0801	C1202	
**W97**	HBe_109-117_	SASSASSCL	C0304	1 patient C0304/0602	C0304			
W100	HBe_59-67_	LSLDVSAAF	C0303				C0303	
W104	HBe_105-113_	YVIGSWGTL	C0304/0303			C0304		C0303
W122	HBe_131-139_	YSHPIILGF	C0302/1202/1203			C1203	C0302>C1202	
W126	HBe_143-151_	FTFSPTYKAF	C0302/0102/1403		C0102	C0302	C1403	
W127	HBe_81-89_	FASPLHVAW	C0302/1202/1203			C1203>C0302	C1202	
**W166**	Pol_407-415_	LLDDEAGPL	C0401	1 patient C0304/0801	C0401	C0304	C0801	
W169	Pol_435-444_	HPAAMPHLL	C0401		C0401			
W171	Pol_539-547_	LYAAVTNFL	C0401/1402/1403/0801		C0401	C0801	C1402	C1403
W173	Pol_547-555_	FADATPTGW	C0401			C0401		
W195	Pol_649-657_	SFVYVPSAL	C1402/1403				C1402>C1403	
W233	Pol_722-730_	VSIPWTHKV	C1502/1203				C1203>C1502	
W235	Pol_646-654_	GSSGLPRYV	C1502				C1502	
W236	Pol_3-11_	KTFGRKLHL	C1502				C1502	
W239	Pol_115-123_	YSLNFMGYV	C1502/1203		C1203	C1502		
**W309**	Pol_328-337_	TRILTIPQSL	C0701	1 patient C0801/1402			C1402>C0701>C0801	
**W4**	Pol_483-491_	LTFGRETVL	C0102/0302/0303/0304/0602/0701/0801/1202/1203	1 patient C0701/0702	C0701>C0102>C0602>C0801>C0302>C1202	C1203>C0304>C0702>C0303		
W16	Pol_537-545_	WSPQAQGIL	C0102				C0102	
**W17**	Pol_62-70_	HSPTSCPPI	C0102	1 patient C0102			C0102	
W18	Pol_756-764_	TTPAQGTSM	C0102				C0102	
**W19**	Pol_296-304_	MMWYWGPSL	C0102/0801/1402/1403	2 patients C0303/1202		C0801>C1402	C0102>C1403	
W20	Pol_431-439_	SLYNILSPF	C0102/0302/1402/1202/0702/1403		C0302>C1202>C0102	C1402>C0702		C1403
W37	Pol_529-537_	LRDSHPQAM	C0602/0702/0701		C0602>C0701		C0702	
**W39**	Pol_268-276_	RRFIIFLFI	C0602	1 patient C0102			C0602>C0102	
**W40**	Pol_426-434_	VRFSWLSLL	C0602/0702/0701	1 patient C0102	C0702>C0701	C0602	C0102	
**W60**	Pol_598-606_	TASPISSIF	C0702/0801/0304/0303/1202/1203/0701	1 patient C0303	C0303>C1203	C0801>C0304	C0701>C1202>C0702	
**W83**	Pol_504-512_	NSTTFHQAL	C0801/0304/0303/1502	1 patient C0303		C1502	C0304>C0801>C0303	
W85	Pol_642-651_	SLDSWWTSL	C0801/0401		C0401		C0801	
**W106**	Pol_667-676_	LSVPNPLGF	C0304/0302/0303/1202	1 patient C0303		C1202	C0302>C0304>C0303	
**W109**	Pol_832-840_	WASVRFSWL	C0304/0303	1 patient C0801/1402	C1402>C0304		C0801>C0303	
W129	Pol_13-21_	QAMQWNSTTF	C0302/1403				C0302>C1403	
W131	Pol_440-448_	IPIPSSWAF	C0302			C0302		
**W174**	Pol_566-574_	LLDPRVRGL	C0401	1 patient C0401	C0401			
W176	Pol_698-706_	LWEWASVRF	C0401		C0401			
**W177**	Pol_776-784_	ILSPFLPLL	C0401	1 patient C0401		C0401		
W196	Pol_48-56_	SWWTSLNFL	C1403/0102			C0102	C1403	
W241	Pol_450-458_	SSSGTVNPV	C1502				C1502	
**W243**	Pol_495-503_	SSWAFARFL	C1502/1203	1 patient C0702		C1203	C1502>C0702	
W246	Pol_591-599_	SARRMETTV	C1502/1203				C1502>C1203	
W305	Pol_587-595_	KRWGYSLNF	C0701		C0701			
W21	HBx_63-71_	FSSAGPCAL	C0102/0401/0602/0801/0304/0302/0303/1402/1202/1502/1203/1403/0701		C0303>C0302>C0701>C0602>C1203	C1502>C1402>C0304>C0801	C1202>C0102>C0401	C1403
W22	HBx_92-100_	VLHKRTLGL	C0102/0602/0702/1402/1403		C0602		C0102>C1402>C0702	C1403
W23	HBx_143-151_	CSPAPCNFF	C0102				C0102	
**W43**	HBx_71-79_	LRFTSARRM	C0702/1402/1403/0701	1 patient C0702/1402			C0701>C1402>C1403	C0702
**W44**	HBx_77-85_	RRMETTVNA	C0602/0702/0701	1 patient C0102/0801	C0702	C0701	C0102>C0602>C0801	
W46	HBx_95-103_	KRTLGLSAM	C0602/0702/1402/1403/0701		C0701	C0602	C1402>C1403	C0702
W64	HBx_55-63_	LRGLPVCAF	C0702/0701			C0701		C0702
**W87**	HBx_1-9_	MAARVCCQL	C0801/0304/0302/0303/1202/1502/1203	1 patient C0302/0702	C1203	C1502>C0304	C0801>C1202>C0302>C0702	C0303
W88	HBx_37-45_	LPSPSSSAV	C0801/0304/0303/0401/0102				C0102>C0304>C0303>C0401	C0801
**W90**	HBx_100-108_	LSAMSTTDL	C0801/0304/0303/1202/1502	2 patients C0304/1203/0303/0702	C0702>C1502		C0304>C1202>C0303	C0801
W137	HBx_104-112_	STTDLEAYF	C0302/04011202/C1203		C1202	C0401>C1203	C0302	
**W178**	HBx_8-16_	QLDPARDVL	C0401	1 patient C0401	C0401			
W266	HBx_75-83_	RGRPVSGPF	C1403					C1403
W286	HBx_26-34_	QAQGILTTV	C1203				C1203	

Each validated epitope peptide (VEP) was bolded.

### 3.2 The candidate epitopes have high conservation of sequences across HBV genotypes

Briefly, a huge number of sequences of HBsAg (n = 1410, 2802, 3124, 1493), HBeAg (n = 2168, 2542, 2669, 1375), HBpol (n = 1160, 2467, 3131, 1276) and HBx (n = 996, 2561, 3682, 1752) for HBV genotype A, B, C, and D were collected from the publicly available HBVdb database (https://hbvdb.lyon.inserm.fr/HBVdb/HBVdbIndex). Multiple sequence alignments were performed using the ClustalW method in the Molecular Evolutionary Genetics Analysis software (MEGA7, version 1.0.0.0), and those positions where a gap were neglected. Then, GeneDoc software (version 2.7.0) was used to analyze the alignment results for defining the conservative region of these sequences across different HBV strains and genotypes. The conservative properties of each amino acid in each protein were judged by the threshold of 100%, 95%, 80%, and followed by highlighting in different colors (red: 100%; yellow: ≥95%; black in grey background: ≥80%; black in white background: <80%). Finally, the 185 candidate epitopes were classified into three categories according to its conservation (≥95%, 80-95%, <80%). Conservation ≥95%: each amino acid with the conservative properties of 95-100%; Conservation 80-95%: at least one amino acid with the conservative properties of ≥80% and <95%; Conservation <80%: at least one amino acid with the conservative properties of <80% (**Supplementary Figure S3**). Finally, the 185 candidate epitopes were classified into three categories according to its conservation (≥95%, 80-95%, <80%) and displayed in **Supplementary Table S2** and **Supplementary Table S3**. Of the 96 HLA-B-restricted candidate epitopes, only 12 peptides exhibit the conservative properties of <80% in the HBV genotype C, while 3 peptides belong to the conservation of <80% in the A and D genotypes. Of the 89 HLA-C-restricted candidate epitopes, no one exhibits the conservative properties of <80% in the repertoire of four HBV genotypes.

### 3.3 57 HLA-B-restricted candidates and 32 HLA-C-restricted candidates were validated as immunogenic epitopes by peptide/PBMCs coculture experiments using PBMCs from 250 CHB patients

**Figure 1:**
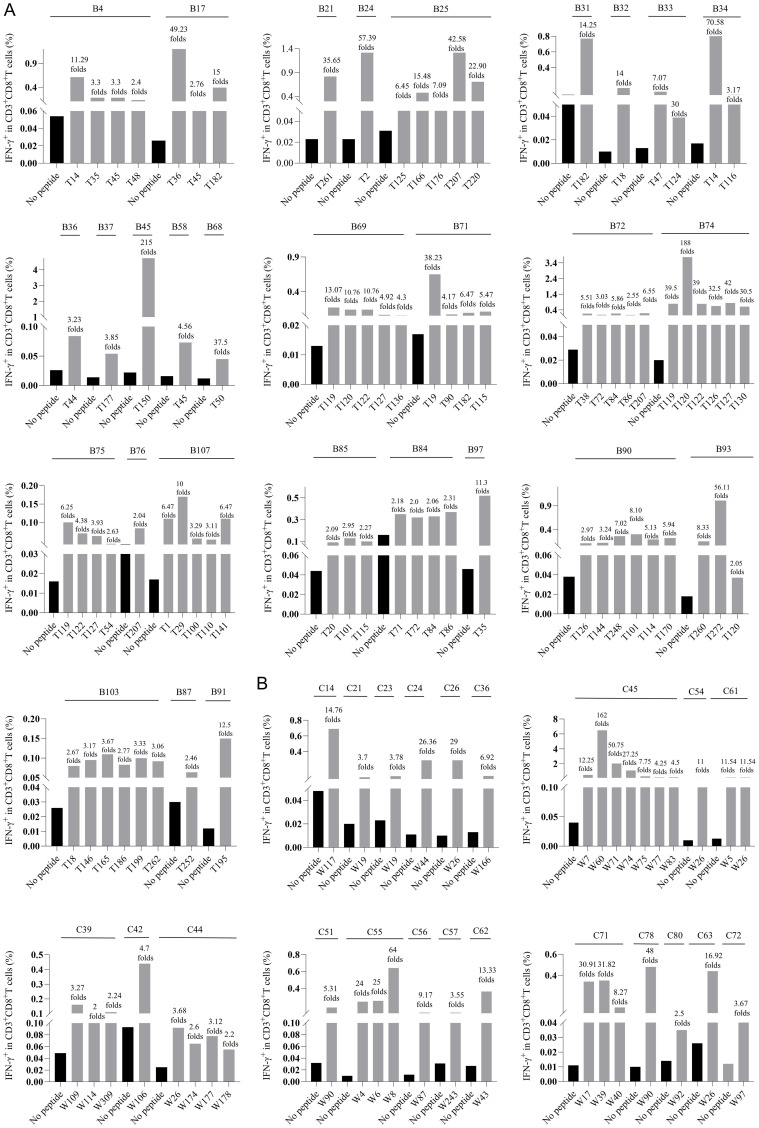
The epitopes inducing CD8^+^ T cell activation in the peptide-PBMCs *ex vivo* co-cultures using PBMCs from 250 CHB patients. Each HBV epitope candidate was co-cultured for 6 hours with PBMCs from the CHB patients with corresponding HLA-B **(A)**, or C **(B)** alleles. Then IFN-γ ICS was used to detect the proportion of IFN-γ^+^ cells in the CD3^+^/CD8^+^ T cell population. The fold changes of IFN-γ^+^/CD8^+^ T cell frequency in the peptide-PBMCs coculture relative to its negative control well (No peptide, PBMCs alone) was presented on top of each column. The serial number of each CHB patient used for HLA-B and HLA-C allele genotyping was displayed as B1 to B150 and C1 to C100. The black bars represent the negative control well (No peptide, PBMCs alone), and the gray bars represent the assay well (immunogenic epitopes validated by *ex vivo* peptide/PBMCs coculture experiments).

In order to determine whether the candidate epitope peptide has immunogenicity in real HBV-infected patients, the peptide/PBMCs coculture experiments were carried out as described with modifications (14, 17). Fresh PBMCs were collected from 250 CHB patients. 150 CHB patients were genotyped for HLA-B alleles followed by peptide/PBMCs cocultures for 96 HLA-B-restricted candidate epitope peptides, and another 100 CHB patients were identified for HLA-C alleles followed by peptide/PBMCs cocultures for 89 HLA-C-restricted candidate epitope peptides. Each candidate epitope peptide corresponding to the indicated HLA-B or C alleles was *ex vivo* co-cultured with the PBMCs from the indicated patient for 6 hours. Flow cytometry was then used to measure the frequency of IFN-γ^+^ cells in the CD3^+^/CD8^+^ T cells. During flow cytometry gating, selecting larger lymphocytes helps exclude a greater number of dead cells. And a PBMC-only well stained with fluorescent antibody was included, which effectively excludes non-specific staining of residual dead cells by fluorescent antibodies. When the frequency of IFN-γ^+^/CD8^+^ T cells in the peptide/PBMCs coculture well was at least twice higher than that in the PBMCs alone well, the candidate epitope peptide was defined as immunogenic epitope peptide, indicating the presence of circulating epitope-specific memory CD8^+^ T cell clone in the patient’s peripheral blood. Totally, of the 96 candidate epitope peptides restricted by HLA-B molecules, 57 peptides were validated as immunogenic peptides (**Figure 1A**, **Table 1**), and 15, 13, 7 and 22 peptides harbor in HBsAg, HBeAg, HBx, and HBpol, respectively. Of the 57 validated peptides in the *ex vivo* peptide-PBMCs cocultures and restricted by HLA-B molecules: 21 exhibited a 2-5-fold increase in frequency, 20 a 5-20-fold increase, and an additional 16 an increase of over 20-fold, all compared with the negative control. In parallel, of 89 candidate epitope peptides restricted by HLA-C molecules, 32 peptides were defined as immunogenic peptides (**Figure 1B**, **Table 2**), and 12, 6, 5 and 9 peptides derive from HBsAg, HBeAg, HBx, and HBpol, respectively. Of the 32 validated peptides in the *ex vivo* peptide-PBMCs cocultures and restricted by HLA-C molecules:13 exhibited a 2-5-fold increase in frequency, 8 a 5-20-fold increase, and an additional 11 an increase of over 20-fold, all compared with the negative control. The FACS gating strategy and IFN-γ intracellular staining data in peptide/PBMCs cocultures for all PBMCs samples were presented in **Supplementary Figure S4–7**.

### 3.4 Binding affinity and cross-binding of 185 candidate epitopes with corresponding HLA-B and C allotypes were analyzed using peptide competitive binding assay

**Figure 2:**
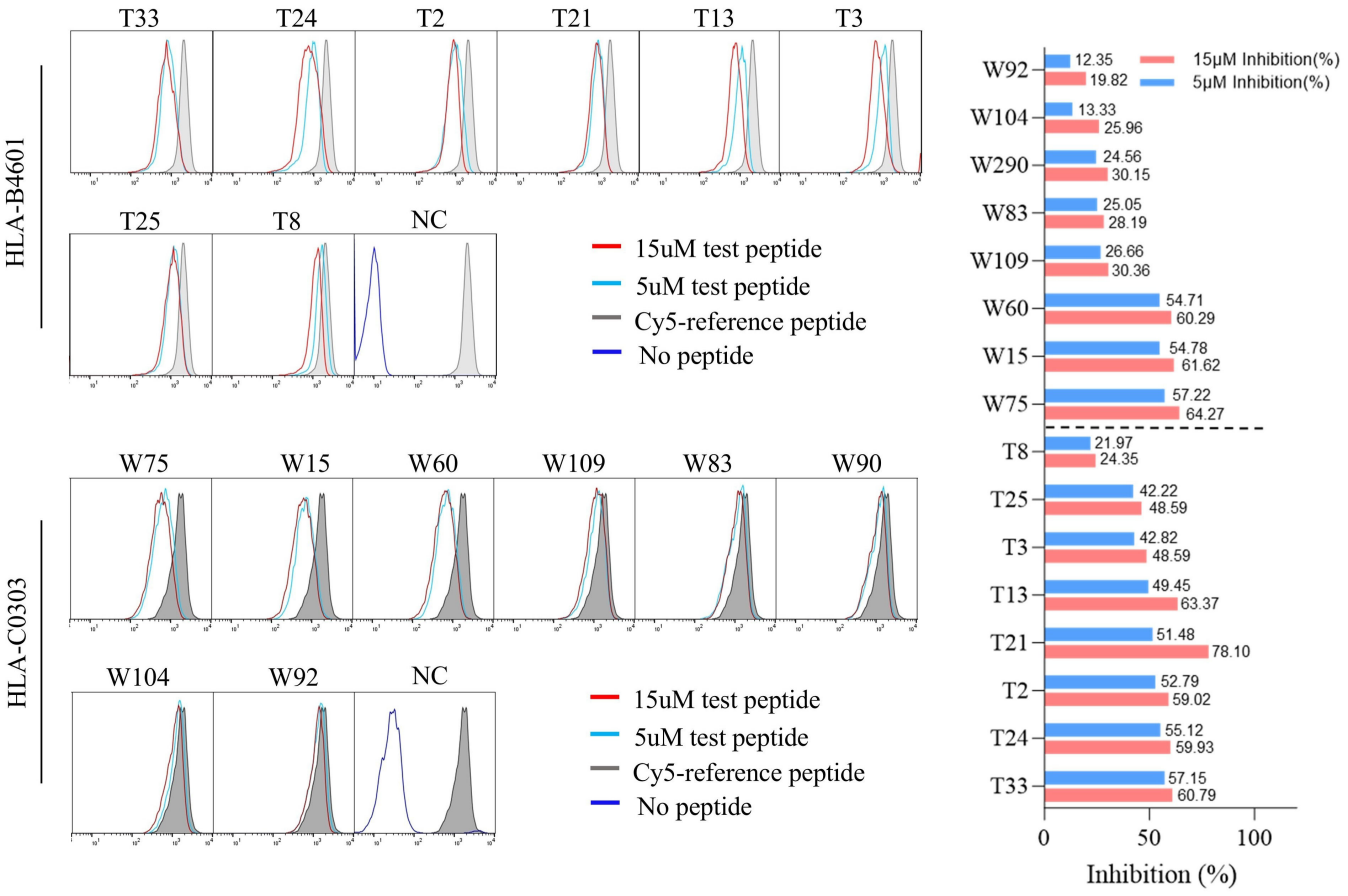
The representative flow cytometric histograms of HBV epitope peptide binding to HLA-B and C allotypes in competitive peptide binding assays. The HMy2.CIR cells constantly expressing indicated HLA-B, C molecule were incubated with Cy5-labeled reference peptide and the no-labeled epitope peptide. After a defined incubation period, the unbound peptides were removed, and the relative binding affinity of the tested epitope peptide to the HLA molecule was quantified by the declined MFI of reference peptide binding to the CIR cell surface at different concentration of tested epitope peptide (5 μM and 15 μM). The red solid line and the blue solid line in the figure show the cell fluorescence peaks of the peptides to be tested at concentrations of 15 µM and 5 µM, respectively, while the gray filled line represents the maximum fluorescence intensity of cells in the presence of only Cy5-reference peptide. The dark blue solid line in the NC figure represents the background fluorescence of cells in the background well. Representative flowcytometry plots reflecting the affinity between epitopes and HLA-B4601 or HLA-C0303. The histogram showed the inhibition rates of each epitope at 5 and 10μM concentrations against the Cy5-reference peptide when incubated with CIR-B4601 or CIR-C0303.

The possible proposed candidate epitope peptides presented by each HLA-B or -C allotype were categorized in a list, and, one by one, they were subjected to the peptide competitive binding assays. Compared with the max control well (CIR cells and Cy5-reference peptide), most of the tested unlabeled peptides led to a leftward shift of the fluorescence peak onto the indicated CIR cell lines (**Figure 2**; **Supplementary Figure S8**), indicating that the unlabeled peptides could efficiently compete with the Cy5-labeled reference peptide for binding to the relevant HLA-B or C molecules. **Supplementary Table S4** and **Supplementary Table S5** displayed the binding affinities between each HLA-B/C allotype and the relevant candidate epitope peptides. Among the 96 candidate epitopes restricted by HLA-B molecules, 48 high-affinity, 12 inter-affinity, 33 low-affinity and 3 no-affinity candidates were defined in the peptide competitive binding assays (**Supplementary Table S4**). For 89 candidate epitopes restricted by HLA-C molecules, 45 high-affinity, 19 inter-affinity, 23 low-affinity and 2 no-affinity candidates were identified (**Supplementary Table S5**).

Among the 57 VEPs validated in the *ex vivo* peptide-PBMCs cocultures and restricted by HLA-B molecules, 39 showed high or intermediate affinity for the relevant HLA-B allotypes (**Table 1**). For the 32 VEPs validated in the *ex vivo* peptide-PBMC cocultures and restricted by HLA-C molecules, 24 showed high or intermediate affinity for the relevant HLA-C allotypes in the competitive peptide binding assays (**Table 2**). These data indicate that the majority of functionally validated epitopes also displayed high or intermediate HLA-binding capacity, supporting a positive relationship between HLA-binding strength and T-cell immunogenicity in our system.

Notably, most of candidate epitope peptides exhibited cross-reactivity with several HLA-B allotypes (64/96) or HLA-C allotypes (69/89) with high or intermediate affinity. Specifically, T33 bound to three HLA-B allotypes with high affinity, while T1, T2, T19, T21, T39, T86, T100, T110, T141, and T166 bound to two HLA-B allotypes with high affinity (**Table 1**). W4 exhibited high affinity with six HLA-C allotypes, while W21 with five allotypes, W10 with four allotypes, and W20 with three allotypes. In addition, candidate epitope peptides such as W60, W71, W37, W114, W109, W90, W31, W2, W38, W8, and W40 displayed high affinity with two HLA-C molecules (**Table 2**).

### 3.5 The broad-spectrum CD8+ T-cell epitope library of HBV main proteins was generated and characterized

Utilizing the functionally validated T-cell epitopes by authors and other researchers, a broad-spectrum CD8^+^ T-cell epitope peptide library was set up. These epitope peptides derived from HBsAg (covering pre-S1, pre-S2 and S), HBeAg (containing HBcAg), HBx, and HBpol proteins, including 103 epitopes restricted by 13 dominant HLA-A allotypes and identified in our previous study (14, 15), 83 epitopes restricted by 15 dominant HLA-B allotypes and 54 epitopes restricted by 14 dominant HLA-C allotypes. Among the 83 HLA-B-restricted epitopes, 57 peptides were validated by *ex vivo* peptide-PBMC cocultures, 18 peptides were defined with high or intermediate affinity by competitive peptide binding assay, and 8 peptides were reported previously (18–21). For the 54 HLA-C-restricted epitopes, 32 peptides were validated by *ex vivo* peptide-PBMC cocultures, 11 were defined with high or intermediate affinity by competitive peptide binding assay, and 1 epitope was reported previously (21). In this epitope library, the numbers of epitopes restricted by each HLA-A, B, or C allotype were designed to match the gene frequency of each HLA allotype. The HLA allotype with higher gene frequency has more epitopes restricted by it. The total gene frequencies of the prevalent 13 HLA-A, 15 HLA-B, and 14 HLA-C allotypes cover over 90%, 70%, and 90% of Northeast Asian populations, respectively, and even more in Chinese population, thus the epitope library has a large population coverage.

As analyzed by a combination of *in silico* prediction, *ex vivo* co-cultures of peptides with PBMCs, and peptide competitive binding assays using HLA-B/C transfected cell lines, many epitope peptides could be cross-presented by 2–5 HLA allotypes (**Tables 1**, **2**). The cross-restriction of each epitope were summarized in **Table S6–S8**. Based on these data, we can infer that the 83 HLA-B-restricted epitopes and 54 HLA-C-restricted epitopes could target 173 and 102 peptide/HLA complex-specific CD8^+^ T cell clones, respectively. Similarly, the previously validated 103 HLA-A-restricted epitope peptides could target at least 262 peptide/HLA complex-specific CD8^+^ T cell clones. Theoretically, the 240 CD8^+^ T-cell epitopes from HBV main proteins could target more than 537 peptide/HLA complex-specific CD8^+^ T cell clones.

Notably, of the 240 validated CD8^+^ T-cell epitopes, 39 ones are sequence-identical epitopes cross-presented by HLA-A, B and C molecules as displayed in **Supplementary Table S9**, and do not need to be used repeatedly. Thus, only 201 sequence-distinct CD8^+^ T-cell epitopes were used to establish the broad-spectrum CD8^+^ T-cell epitope library and synthesized as peptides for further use in ELISpot assay. Of the 201 epitopes, 73, 58, 42, and 28 peptides harbor in HBpol (845 aa), HBsAg (covering pre-S1, pre-S2 and S, 400 aa), HBeAg (containing HBcAg, 214 aa) and HBx (154 aa) proteins, respectively, with a similar epitope distribution density in each protein (**Table 3**).

**Table 3:** Peptide pools of 201 T-cell epitopes used in the in-house ELISpot assay.

Peptide pool	Pool 1	Pool 2	Pool 3	Pool 4	Pool 5	Pool 6	Pool 7	Pool 8	Pool 9	Pool 10	Pool 11
Derived protein	HBsAg	HBsAg	HBsAg	HBpol	HBpol	HBpol	HBpol	HBx	HBx	HBe/cAg	HBe/cAg
Kinds of peptide	20	20	18	20	20	17	16	15	13	20	22

The conservative properties of the 201 CD8^+^ T-cell epitopes across HBV genotypes were further analyzed as described above, and summarized in **Supplementary Figure S9**. Among the 201 epitope peptides, the numbers of epitope peptides belonging to HBV genotypes C, B, A, and D are 179, 138, 119, and 111, respectively. For the prevalent B and C genotypes of HBV, only 5 and 12 epitope peptides, respectively, have conservation properties of <80%.

Taken together, the CD8^+^ T-cell epitope library of HBV is highly conservative for HBV prevalent genotypes, broad-spectrum for the HLA-A\B\C genetic polymorphism of Northeast Asian population, and comprehensively representative for the richness of epitopes in each HBV protein.

### 3.6 The ELISpot assay for counting reactive HBV-specific T cells was established and validated in methodology

**Figure 3:**
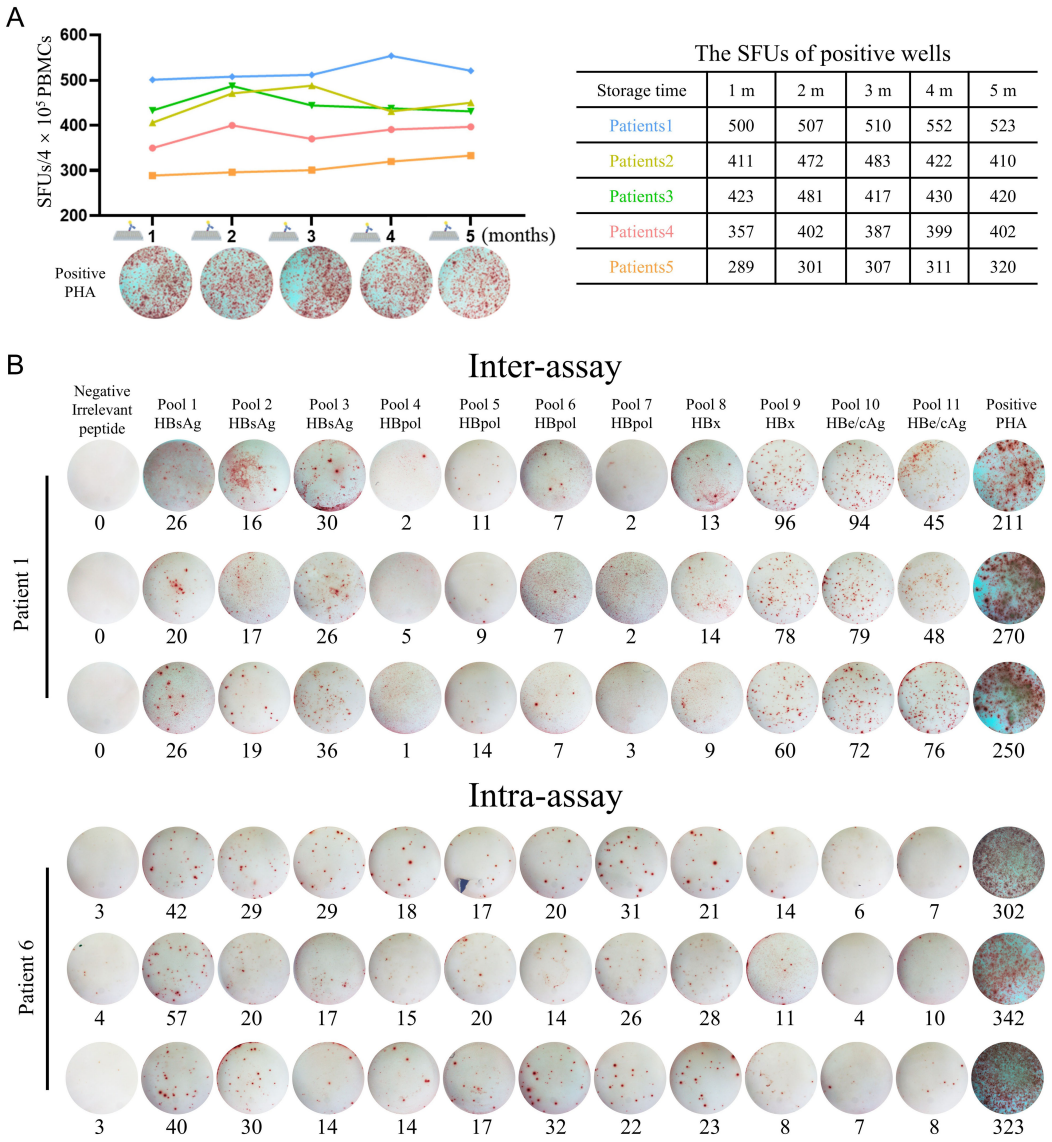
Methodological validation of the improved universal ELISpot assay. **(A)** A series of PVDF membrane plates were pre-coated with antibodies with an interval of one month and immediately stored at 4°C. When the first-batch PVDF membrane plate was stored for 5 months, all batches of plates were harvested and seeded with PBMCs from same CHB patient, then cocultured with PHA followed by ELISpot assay. The SFUs in five patients were summarized in the table. **(B)** For intra-assay, PBMCs from each CHB patient (n = 5) were detected three times under the same conditions by a single operator. For inter-assay, PBMCs from each CHB patient (n = 5) were divided into three equal parts and detected by three independent operators, respectively, under the same conditions. Dot plots of IFN-γ ELISpot from representative subjects were presented.

According to the derived HBV protein and the physiochemical features, the 201 CD8^+^ T-cell epitope peptides were grouped into 11 peptide pools (**Table 3**). Firstly, the stability of PVDF membrane plates pre-coated with IFN-γ capture antibodies was investigated. A series of PVDF membrane plates were pre-coated with antibodies with an interval of one month and immediately stored at 4 °C. When the first-batch PVDF membrane plate was stored for 5 months, all batches of plates were harvested and seeded with PBMCs derived from same CHB patient, then cocultured with PHA followed by ELISpot assay. There was no statistical difference across the SFUs counts developed in the plates which stored for 1 to 5 months at 4 °C (**Figure 3A**).

To evaluate the reproducibility or accuracy of the in-house ELISpot assay, Intra-assay and inter-assay experiments were carried out. For the intra-assay experiment, a single operator performed three tests on the PBMCs of each CHB patient (n = 5) under the same conditions. For the inter-assay experiment, the PBMCs of each CHB patient (n = 5) were divided into three equal parts and conducted by three independent operators. As shown in **Figure 3B**, when 3 aliquots of the same PBMCs sample were tested by a single operator or 3 independent operators, there was no significant difference between the results. The intra- and inter-assay coefficients of variation (CV) of the experiments were 7.22% and 7.84%, respectively (**Supplementary Table S10**).

### 3.7 Clinical tests of reactive HBV-specific T cells using the in-house ELISpot assay

81 CHB patients and 10 HBV-infected LC patients were enrolled in the clinical tests. PBMCs were tested using the 201 CD8^+^ T-cell epitope peptides (11 peptide pools) and in-house ELISpot assay to count reactive HBV-specific T cells (mainly CD8^+^ T cells). The clinical characteristics of the patient cohort at the time of testing are presented in **Supplementary Table S11**. CHB patients showed significantly higher HBsAg levels (388.5 *vs* 186.9 IU/mL, p = 0.024) and HBeAg levels (8.26 *vs* 0.38 COI, p = 0.022) than HBV-infected LC patients (**Supplementary Table S11**). The spot plots reactive to each peptide pool in the in-house ELISpot assay were presented for three representative CHB subjects and two representative LC subjects (**Supplementary Figure S10A**). As shown in **Supplementary Figure S10B**, CHB patients displayed obviously more reactive HBV-specific T cells than HBV-infected LC patients (median SFUs 124 *vs*. 40). HBsAg-, HBpol-, HBe/cAg-, and HBx-specific T cells also displayed the trends similar to total HBV-specific T cells between the two groups (**Supplementary Figure S10B**). ROC analysis confirmed that the counts of HBV-specific T cells (AUC: 0.857; cut-off value: 67.5 SFUs/4×10^5^ PBMCs) and HBsAg-specific T cells (AUC: 0.877; cut-off value:14 SFUs/4×10^5^ PBMCs) exhibited good performance in discriminating between CHB and HBV-infected LC (**Supplementary Figure S10C**).

This finding is, to some extent, consistent with previous researches. The patients with LC frequently exhibit lymphopenia, perturbations of T-cell subsets, and impaired effector function, suggesting that adaptive antiviral immunity may be further compromised at advanced stages of liver disease (22). Therefore, the findings observed in HBV-specific T cells between CHB and LC possibly reflect a suppressive immune dysfunction at the LC stage. Of note, some patients classified as having CHB in this study may have had underlying cirrhosis, but liver biopsy was not performed to confirm the histological diagnosis. Additionally, the sample sizes of the two groups were significantly unbalanced. Therefore, this finding requires further research for confirmation.

### 3.8 Stratified analyses of HBV-specific T cell reactivity in subgroups of CHB patients with different clinical profiles

**Figure 4:**
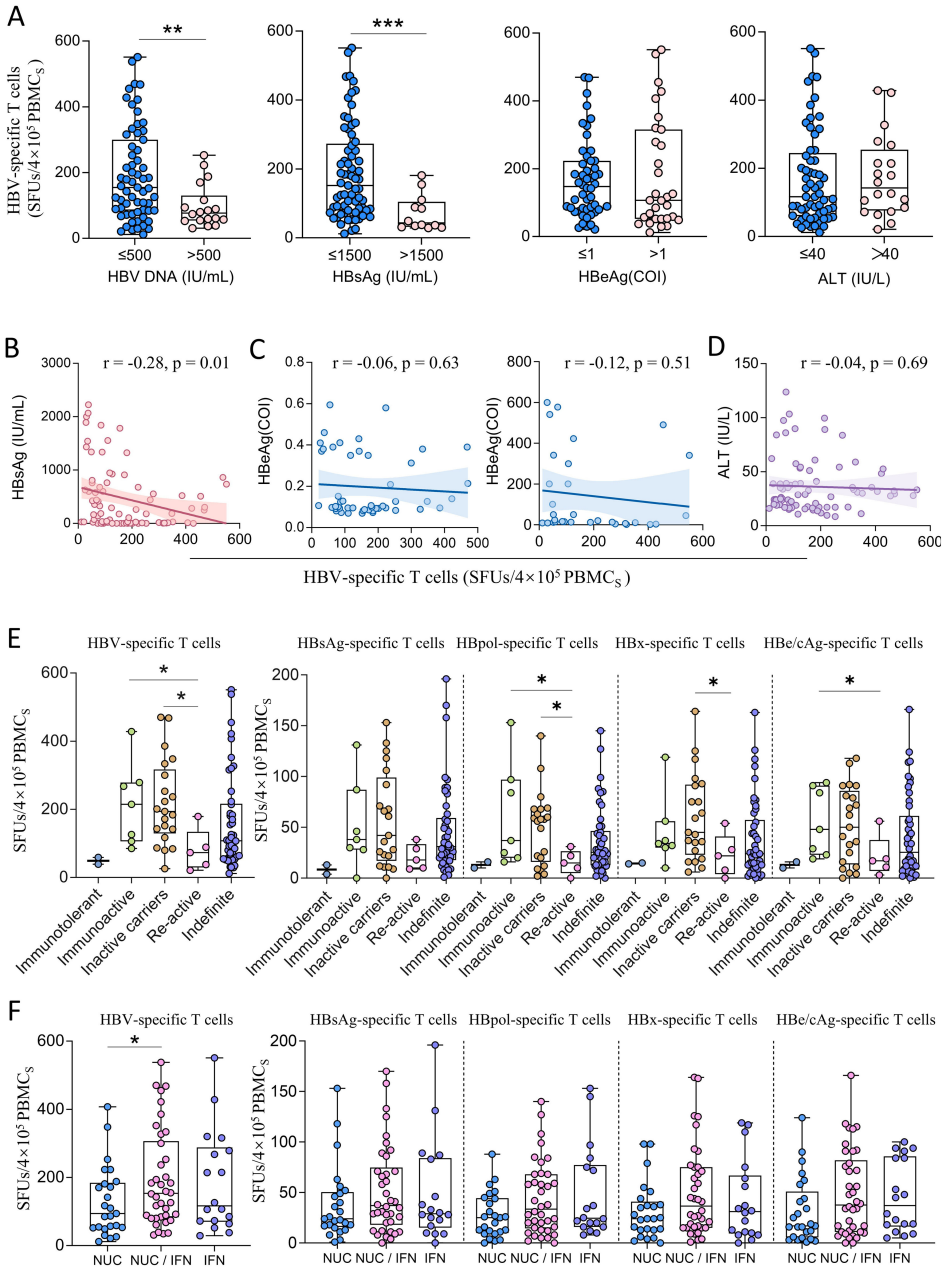
The comparison of HBV-specific T cell reactivity among CHB patients with different clinical profiles. **(A)** Stratified analyses of HBV-specific T cells (SFUs) in CHB patients grouped by HBV DNA load (≤500, n=63; >500, n=18), HBsAg level (≤1500, n=69; >1500, n=12), HBeAg level (≤1, n=50; >1, n=31) and ALT level (≤40, n=61; >40, n=20). **(B)** Spearman correlation tests between counts of reactive HBV-specific T cells (SFUs) and HBsAg levels. **(C)** Spearman correlation tests between counts of reactive HBV-specific T cells (SFUs) and HBeAg levels in either the HBeAg-negative group (left) or the HBeAg-positive group (right). **(D)** Spearman correlation tests between counts of reactive HBV-specific T cells (SFUs) and ALT levels. **(E)** Total HBV-specific T cells (SFUs) in CHB patients at different clinical phases (Immunotolerant, n = 2; Immunoactive, n = 7; Inactive carriers, n = 21; Re-active, n = 5; Indefinite phases, n = 46). **(F)** HBV-specific T cells (SFUs) in CHB patients with different treatments (left). Specific T cells (SFUs) reactive to each HBV protein (HBsAg, HBpol, HBx, HBe/cAg) in different treatment groups (right). NUCs monotherapy, n = 25; NUCs/IFN combination therapy, n = 38; IFN monotherapy, n = 18. *p < 0.05; **p < 0.01; ***p < 0.001.

The CHB patients with low HBV-DNA loads (≤500 IU/mL) and low HBsAg levels (≤1500 IU/mL) showed significantly more reactive HBV-specific T cells than the CHB patients with high viral loads (>500 IU/mL) and high HBsAg levels (>1500 IU/mL) (**Figure 4A**). But spearman correlation tests confirmed that the counts of reactive HBV-specific T cells in PBMCs only weakly and negatively correlated with serum HBsAg levels (r =  -0.28) (**Figure 4B**), but did not correlated with HBeAg levels or ALT levels (**Figures 4C, D**). The HBsAg-, HBpol-, HBx-, or HBe/cAg-specific T cells also negatively correlated with serum HBsAg levels with a low coefficient (r = -0.16 ~ -0.30) (**Supplementary Figure S11A**), but did not correlate with HBeAg levels in either the HBeAg-negative group (upper) or the HBeAg-positive group (lower) (**Supplementary Figure S11B**) or ALT levels (**Supplementary Figure S11C**). Regrettably, due to the fact that 63 out of 81 CHB patients had HBV DNA levels below the limit of detection (<500 IU/mL), the remaining sample size was insufficient to analyze the correlation between HBV-specific T cells and viral DNA loads. The numbers of reactive HBV-specific T cells in PBMCs were compared across the clinical phases of CHB patients. 81 CHB patients enrolled in this study were grouped into immunotolerant, immunoactive, inactive carriers, re-active and indefinite phases according to Guidelines for the Prevention and Treatment of Chronic Hepatitis B (2022 Version). As shown in **Figure 4E**, immunoactive patients (median 215 SFUs) and inactive carriers (median 194 SFUs) displayed the significantly higher HBV-specific T cell reactivity than re-active patients (median 73 SFUs). Notably, the trends of HBpol-, HBx- or HBe/cAg-specific T cells were similar to that of total HBV-specific T cells, but HBsAg-specific T cells displayed no significant differences across groups.

Furthermore, the counts of reactive HBV-specific T cells in PBMCs in NUCs/IFN-α combination group (median 153.5 SFUs) were obviously higher than those in the NUCs (median 94 SFUs) or IFN-α monotherapy (median 116.5 SFUs) groups (**Figure 4F**). The multivariate linear regression analysis also confirmed the correlation between counts of reactive HBV-specific T cells in PBMCs and HBsAg level (p = 0.01) or treatment regimen (p = 0.04).

## 4 Discussion

Restoring or enhancing HBV-specific T cell reactivity is a key strategy for achieving functional cure in CHB patient (23, 24). The increase of reactive HBV-specific T cells in peripheral blood is considered an important biomarker for the safe discontinuation of treatment in CHB patients (25). Therefore, monitoring the dynamic changes in HBV-specific T cell reactivity through routine tests is of great significance for the treatment and management of CHB patients.

Currently, the clinical testing of HBV-specific T cells is greatly limited by the lack of broad-spectrum T-cell epitopes that are compatible with the regionally prevalent HLA supertypes. The reported tests were relying on the uses of custom-designed overlapping peptide (OLP) libraries or *in silico* predicted peptide libraries of indicated HBV protein in ELISpot assay, FluroSpot assay, ICS with flow cytometry, or peptide-HLA tetramer enrichment (8, 26–29). However, recent researches have confirmed that most of these overlapping or predicted peptide segments could be pseudo-epitopes (30–32), and the overlapping peptides induced much weaker T cell activation than the functionally validated T-cell epitopes in ELISpot assay (33). Moreover, OLP is a high-cost and labor-intensive method. For CD8^+^ T cell epitopes, HBsAg, HBeAg, HBx, and HBpol contain 131, 68, 49, and 279 OLPs, respectively, when each 9-mer peptide overlaps by six amino acids. Thus, considerable efforts have been devoted to screening the real-world T-cell epitope library, which contributes to developing a more effective detection system for routine and more sensitive enumeration of reactive HBV-specific T cells. Although many studies have reported that CHB patients often lack HBsAg or HBV-specific T cell responses, in our previous studies, the reactive HBsAg-, HBx-, HBc/eAg-, and HBpol-specific T cells in CHB patients were successfully and routinely enumerated by using IFN-γ ELISpot assay and 103 validated T-cell epitope peptides (which restricted by 13 prevalent HLA-A allotypes) for the random Northeast Asian patients (15). This study aims to improve the ELISpot assay by using the comprehensive HBV-specific CD8^+^ T cell epitope peptides restricted by prevalent HLA-A, B, and C allotypes rather than the T-cell epitopes restricted only by prevalent HLA-A molecules. What deserves our attention is that this study differs from previous ones in three aspects.

First, this is a large-scale screening for a broad-spectrum CD8^+^ T-cell epitope library, comprising three aspects. The epitopes restricted by a series of prevalent HLA-B and HLA-C allotypes were screened, which cumulatively cover 70% and 90% of Northeast Asian populations, respectively. The entire amino acid sequences of HBsAg (covering pre-S1, pre-S2 and S), HBeAg (containing HBcAg), HBx and HBpol proteins from the C, B, A and D genotypes of HBV were screened using PBMCs from 250 CHB patients. The combined evaluation system of *in silico* prediction, cellular functional validation and peptide competitive binding assay was used to validate these epitopes. However, it still does not guarantee that all epitopes have been identified, and additional methods can still be incorporated, such as mass spectrometry analysis for the peptides eluted from HLA molecules.

Secondly, it is well-known that a T-cell epitope can usually be presented by several HLA allotypes with different binding affinities, which is referred to as HLA cross-restriction. However, due to the lack of standardized methods, it is often challenging to determine the HLA restriction of T-cell epitope. Therefore, most previously reported HBV T-cell epitopes lack detailed HLA restriction information, especially regarding cross-restriction by multiple HLA allotypes. In this study, not only were the immunogenicities of 57 T-cell epitopes restricted by HLA-B molecules and 32 T-cell epitopes restricted by HLA-C molecules verified through *ex vivo* cellular functional assays, but also the HLA cross-restrictions of these epitope peptides were further analyzed through peptide competitive binding experiments of HLA molecules using a in-house cell line array.

Thirdly, in combination with peptides previously reported by our group (14), this study provides a CD8^+^ T-cell epitope library of HBV main protein, which is highly conservative for HBV prevalent genotypes, has large coverage for HLA-A/B/C genetic polymorphisms in Northeast Asia, and can comprehensively represent the richness of epitopes in each HBV protein. This achievement provides a universal tool for counting reactive HBV-specific CD8^+^ T cells. Direct *ex vivo* ELISpot assay using this peptide library avoids biases introduced by *in vitro* expansion (e.g., IL-2 stimulation), which may artificially restore functionality in exhausted T cells and misrepresent the *in vivo* immune status. In addition, this HBV T-cell epitope library also lays a foundation for vaccine design and the development of immunotherapy strategies based on T-cell epitopes.

Accumulating evidence indicates that both the quantity and functional quality of HBV-specific T cells not only shape the natural course of infection but also serve as key immunological predictors of a functional cure. In NUC withdrawal studies, patients who retain a relatively large pool of functionally competent, minimally exhausted HBV-specific T cells at or shortly before treatment cessation were more likely to achieve sustained virological control and even HBsAg loss (8, 23, 24, 34, 35). In NUC treatment study, compared with treatment-naïve patients and those with spontaneous viral control, NUC-treated patients displayed HBV-specific T cell responses skewed toward IFN-γ and IL-2 production, with relatively low granzyme B (GrzB) secretion (36). Our study did not find the IFN-γ production increase of HBV-specific T cells during NUC treatment, but the pegIFN-α/NUCs combination therapy could markedly enhance HBV-specific T cell responsiveness when compared to NUC or pegIFN-α monotherapy. Consistently, Tenofovir monotherapy did not substantially restore HBV-specific T cell function, although HBV DNA, HBsAg and ALT levels decline during therapy (37, 38). By contrast, co-administration of Tenofovir with GS-4774 (a therapeutic HBV vaccine) significantly enhanced production of IFN-γ, TNF-α and IL-2 of HBV-specific CD8^+^ T cells (38). In addition, IFN-α monotherapy could promote interleukin-12 (IL-12) production and partially restore HBV-specific T cell function (39), and NUCs/IFN-α combination therapy restored HBV-specific T cell activation and differentiation in CHB patients (40). Taken together, these data suggest that future functional cure strategies for CHB are likely to prioritize pegIFN-α-based regimens combined with NUCs. Such combinations not only suppress viral replication but also enhance antiviral immunity, thereby increasing the likelihood of durable responses and reducing the risk of relapse.

The omission of viability staining in the Peptide-PBMCs coculture experiment has certain limitations, as viable cell proportions often vary significantly across human individuals and dead cells can exhibit substantial non-specific binding of stained antibodies. However, the following measures were performed to minimize the impact of dead cells. All PBMCs in this study were processed immediately after blood collection. Moreover, during flow cytometry gating, dead cells typically display a smaller size and localize to the lower-left quadrant of the scatter plot, enabling discrimination based on reduced forward scatter (a surrogate for cell volume). Thus, the gated population primarily consists of viable lymphocytes. Meanwhile, a PBMC-only control well stained with fluorescent antibodies were included, which effectively eliminates non-specific binding interference between residual dead cells and fluorescent antibodies. In future studies, we will incorporate viability staining as a routine component in subsequent ICS experiments to minimizing non-specific staining interference.

In conclusion, 89 novel CD8^+^ T-cell epitopes restricted by prevalent HLA-B and C allotypes were identified from HBV main proteins. Furthermore, 201 validated CD8^+^ T-cell epitope peptides restricted by predominant HLA-A, B, and C allotypes were integrated to construct a broad-spectrum epitope peptide library of HBV proteins. Using this library, an optimized ELISpot assay was established for the routine detection of reactive HBV-specific T cells in CHB patients. The counts of reactive HBV-specific T cells in PBMCs showed a negative correlation with serum HBsAg levels and no correlation with HBeAg or ALT levels. NUCs/IFN-α combination elicited significantly more reactive HBV-specific T cells than NUCs or IFN-α monotherapy.

## Data Availability

The original contributions presented in the study are included in the article/**Supplementary Material**. Further inquiries can be directed to the corresponding author.
