# Supplementary material for: Large-scale screening of HBV epitopes restricted by multiple prevalent HLA-B/C allotypes and routine detection of HBV-specific T cells in CHB patients
Source: Front Immunol. 2026 Jan 9;16:1717543. doi: 10.3389/fimmu.2025.1717543 (PMC12827522; doi:10.3389/fimmu.2025.1717543)
Supplement: Supplementary file 1 [file DataSheet1.pdf]

Large-scale screening of HBV epitopes restricted by multiple prevalent HLA-B/C allotypes and routine detection of HBV-specific T cells in CHB patients

Yandan Wu, Yu Zhao, Ruixue Ji, Pinqing Li, Huijuan Chen, Fangping Yue, Yi Wu, Jie Qiu, Chuanlai Shen

Corresponding author:
Chuanlai Shen, Ph.D., Professor, Department of Microbiology and Immunology, Medical School of Southeast University, Nanjing, Jiangsu, China 210009
E-mail: chuanlaishen@seu.edu.cn

Supplementary Results

Table S1 The Cy5-labeled reference peptides for each HLA-B or C allotype

**Reference peptides**	**HLA-B or C allotypes**
Cy5-SLRGLPVCAF	HLA-B4601, B1301
Cy5-MENTTSGFL	HLA-B5401
Cy5-VSYVNVNMGL	HLA-B5801, B4801, C0102, C0401, C0702, C0801, C1402
Cy5-MMWYWGPSL	HLA-B1502, B1302, B0702, B5201, C0303, C1202
Cy5-SASSASSCL	HLA-B3501, C0304, C0602
Cy5-LSAMSTTDL	HLA-C1203
Cy5-LPLLPIFFCL	HLA-B4001, B5101, B1501
Cy5-AEWDRLHPV	HLA-B4006
Cy5-KRQDILDLWVY	HLA-C0701
Cy5-LLHERLDEF	HLA-C0302
Cy5-QQNWWTLLV	HLA-C1502
Cy5-CYMEAVAL	HLA-B4403, C1403

Table S2 The sequences, genotypes and conservative properties of 96 candidate $CD8^+$ T-cell epitope peptides restricted by prevalent HLA-B molecules

peptide	protein	start	end	sequence	genotypes	conservative properties
T2	HBsAg	16	24	LSVPNPLGF	A/B/C	> 95%
T261	HBsAg	25	33	FPDHQLDPA	A/B/C/D	C: < 80%;A/B/D:> 95%
T231	HBsAg	28	36	HQLDPAFGA	A/C	> 95%
T263	HBsAg	46	55	NPNKDHWPEA	A/C	C:80-95%; A: < 80%
T233	HBsAg	81	90	AQGILTTVPA	C	< 80%
T146	HBsAg	99	108	RQSGRQPTPI	C	> 95%

T1	HBsAg	119	127	AMQWNSTTF	B/C	C: > 95%; B:80-95%
T150	HBsAg	175	183	MENTTSGFL	C	< 80%
T207	HBsAg	186	194	LLVLQAGFF	A/B/C/D	A/C/D: > 95%; B:80-95%
T177	HBsAg	248	256	WMCLRRFII	A/B/C/D	A/C/D: > 95%; B:80-95%
T27	HBsAg	252	261	RRFIIFLFIL	A/C/D	> 95%
T176	HBsAg	272	280	LDYQGMLPV	A/B/C/D	> 95%
T267	HBsAg	308	317	FPSCCCTKPS	C	> 95%
T64	HBsAg	321	330	CTCIPIPSSW	A/B/C/D	> 95%
T262	HBsAg	324	333	IPIPSSWAFA	A/B/C	> 95%
T29	HBsAg	274	283	YQGMLPVCPL	A/B/C/D	> 95%
T3	HBsAg	344	353	FSWLSLLVPF	A/B/C/D	> 95%
T206	HBsAg	349	357	LLVPFVQWF	A/B/C/D	> 95%
T120	HBsAg	351	360	VPFVQWFAGL	C	80-95%
T119	HBsAg	361	369	SPTVWLSVI	A/B/C	B/C: > 95%; A: < 80%
T90	HBsAg	371	379	MMWYWGPSL	A/C/D	C: > 95%; A/D:80-95%
T33	HBsAg	380	389	YNILSPFLPL	C	< 80%
T122	HBsAg	387	396	LPLLPIFFCL	C/D	> 95%
T116	HBsAg	390	398	LPIFFCLWV	A/C	> 95%
T124	HBeAg	1	9	MQLFHLCLI	A/B/C/D	> 95%
T126	HBeAg	14	22	CPTVQASKL	A/B/C/D	> 95%
T69	HBeAg	18	26	QASKLCLGW	A/B/C/D	> 95%
T101	HBeAg	26	35	WLRGMDIDTY	C	> 95%
T13	HBeAg	38	47	FGASVELLSF	B/C	> 95%
T70	HBeAg	45	53	LSFLPSDFF	A/B/C/D	A/B/C: > 95%; D:80-95%
T127	HBeAg	48	56	LPSDFFPSI	B/C	> 95%
T38	HBeAg	68	76	REALESPEH	A/B/C/D	A/B/C: > 95%; D:80-95%
T272	HBeAg	78	87	SPHHTALRQA	B/C/D	> 95%
T40	HBeAg	92	101	GELMNLATWV	B/C	> 95%
T35	HBeAg	105	113	LEDPASREL	B/C	> 95%
T125	HBeAg	107	115	DPASRELVV	B/C	> 95%
T39	HBeAg	111	120	RELVVSYVNV	B/C	80-95%
T72	HBeAg	115	124	VSYVNVNMGL	B/C	80-95%
T71	HBeAg	122	131	MGLKIRQLLW	A/B/C	> 95%
T182	HBeAg	127	135	RQLLWFHIS	A/B/C/D	> 95%
T100	HBeAg	131	139	WFHISCLTF	A/B/C/D	> 95%
T8	HBeAg	139	147	FGRETVLEY	A/B/C	> 95%
T36	HBeAg	141	149	RETVLEYLV	A/B/C	> 95%
T273	HBeAg	166	175	APILSTLPET	A/B/C/D	> 95%
T270	HBeAg	184	192	SPRRRTPSP	A/B/C/D	> 95%
T61	HBpol	5	14	YQHFRKLLLL	A/C	> 95%
T55	HBpol	16	25	DEAGPLEEEL	C/D	> 95%
T257	HBpol	23	31	LEEELPRLA	A/B/C/D	> 95%
T48	HBpol	40	48	AEDLNLGNL	A/C/D	> 95%
T86	HBpol	65	74	STVPVFNPEW	C	< 80%
T201	HBpol	74	82	WQTPSFPNI	C	< 80%
T199	HBpol	93	101	QQYVGPLTV	C	> 95%
T144	HBpol	111	119	MPARFYPNL	C	80-95%
T50	HBpol	162	170	RETTRSASF	A/C	> 95%
T52	HBpol	175	184	WEQELQHGRL	C	80-95%

T260	HBpol	302	311	VELHNIPPSC	C	< 80%
T57	HBpol	331	339	SEPCSDYCL	C	< 80%
T252	HBpol	353	362	TEHGEHNIRI	C	< 80%
T195	HBpol	370	378	RVTGGVFLV	A/B/C/D	> 95%
T54	HBpol	384	392	TESRLVVDF	B/C	> 95%
T166	HBpol	389	397	RLVVDFSQF	A/B/C/D	> 95%
T203	HBpol	393	402	SQFSRGSTHV	C	80-95%
T20	HBpol	409	417	FAVPNLQSL	A/B/C/D	> 95%
T21	HBpol	428	436	LSLDVSAAF	A/B/C/D	> 95%
T141	HBpol	440	449	HPAAMPHLLV	B/C/D	> 95%
T280	HBpol	444	452	MPHLLVGSS	B/C/D	80-95%
T165	HBpol	502	510	KLHLYSHPI	A/B/C/D	> 95%
T47	HBpol	529	538	AQFTSAICSV	A/B/C/D	> 95%
T19	HBpol	541	549	RAFPHCLAF	A/B/C/D	> 95%
T143	HBpol	547	556	LAFSYMDDVV	A/B/C/D	> 95%
T84	HBpol	560	568	KSVQHLESL	A/C/D	C: > 95%; A:80-95%; D: < 80%
T167	HBpol	594	602	SLNFMGYVI	A/B/C	A: > 95%; B/C:80-95%
T228	HBpol	633	642	CQRIVGLLGF	B/C/D	> 95%
T110	HBpol	640	648	GLLGFAAPF	A/B/C/D	> 95%
T25	HBpol	644	653	FAAPFTQCGY	A/B/C/D	> 95%
T170	HBpol	661	669	IQSKQAFTF	C/D	C: < 80%; D:80-95%
T115	HBpol	667	675	QAFTFSPTY	A/B/C/D	A/B/C: > 95%; D:80-95%
T114	HBpol	676	685	FTFSPTYKAF	A/B/C/D	A/B/C: > 95%; D:80-95%
T59	HBpol	679	687	KQYLHLYPV	C	< 80%
T248	HBpol	730	738	AELLAACFA	A/B/C/D	> 95%
T136	HBpol	760	768	FPWLLGCAA	B/C/D	C/D: > 95%; B:80-95%
T111	HBpol	772	781	WILRGTSFVY	A/B/C/D	> 95%
T24	HBpol	834	842	FASPLHVAW	A/B/C/D	> 95%
T134	HBx	1	9	MAARVCCQL	B/C	> 95%
T162	HBx	7	16	CQLDPARDVL	B/C/D	C/D:80-95%; B: > 95%
T130	HBx	10	18	DPARDVLCL	B/C/D	C/D:80-95%; B: > 95%
T186	HBx	13	21	RDVLCLRPV	A/B/C	B/C: > 95%; A:80-95%
T160	HBx	36	45	KEFGASVELL	B/C	> 95%
T18	HBx	54	63	SLRGLPVCAF	A/B/C/D	> 95%
T14	HBx	63	71	FSSAGPCAL	A/B/C/D	A/C/D:＞95%; B:80-95%
T132	HBx	89	98	LPKVLHKRTL	A/B/C/D	C:80-95%; A/B/D: > 95%
T16	HBx	102	111	AMSTTDLEAY	A/B/C	C:80-95%; A/B: > 95%
T44	HBx	120	129	WEELGEEIRL	A/B/C/D	80-95%
T45	HBx	125	134	EEIRLMVFVL	C	< 80%
T220	HBx	141	150	LVCSPAPCNF	B/C	80-95%
T275	HBx	146	154	APCNFFTSA	B/C/D	C/D:80-95%; B: > 95%

Table S3 The sequences, genotypes and conservative properties of 89 candidate $CD8^{+}$ T-cell epitope peptides restricted by prevalent HLA-C molecules

peptide	protein	start	end	sequence	genotypes	conservative properties
W16	HBsAg	77	85	WSPQAQGIL	C	80-95%

W106	HBsAg	16	24	LSVPNPLGF	A/B/C	> 95%
W109	HBsAg	339	347	WASVRFSWL	A/B/C	> 95%
W129	HBsAg	118	127	QAMQWNSTTF	B/C	C: > 95%; B:80-95%
W131	HBsAg	324	332	IPIPSSWAF	A/B/C/D	> 95%
W17	HBsAg	234	242	HSPTSCPPI	A/C	C:80-95%; A: > 95%
W18	HBsAg	299	307	TTPAQGTSM	B/C	80-95%
W19	HBsAg	371	379	MMWYWGPSL	A/C/D	C: > 95%; A/D:80-95%
W20	HBsAg	378	386	SLYNILSPF	A/C	80-95%
W37	HBsAg	112	120	LRDSHPQAM	A/C	C:80-95%;
W38	HBsAg	246	254	YRWMCLRRF	A/B/C/D	A/C/D: > 95%; B:80-95%
W39	HBsAg	252	260	RRFIIFLFI	A/C/D	> 95%
W40	HBsAg	342	350	VRFSWLSLL	A/B/C	> 95%
W60	HBsAg	157	165	TASPISSIF	C	80-95%
W83	HBsAg	123	131	NSTTFHQAL	C	> 95%
W85	HBsAg	205	213	SLDSWWTSL	A/B/C/D	> 95%
W174	HBsAg	131	139	LLDPRVRGL	C	> 95%
W176	HBsAg	336	344	LWEWASVRF	A/C	> 95%
W177	HBsAg	382	390	ILSPFLPLL	C/D	80-95%
W196	HBsAg	208	216	SWWTSLNFL	A/B/C/D	A/C: > 95%; B/D:80-95%
W241	HBsAg	146	154	SSSGTVNPV	C/D	80-95%
W243	HBsAg	328	336	SSWAFARFL	C	> 95%
W286	HBsAg	80	88	QAQGILTTV	C	80-95%
W309	HBsAg	197	206	TRILTIPQSL	A/C/D	A/C: > 95%; D:80-95%
W1	HBeAg	47	56	FLPSDFFPSI	B/C	> 95%
W2	HBeAg	52	60	FFPSIRDLL	C	> 95%
W4	HBeAg	137	145	LTFGRETVL	A/B/C	> 95%
W5	HBeAg	76	84	HCSPHHTAL	A/B/C/D	> 95%
W6	HBeAg	160	169	AYRPPNAPIL	A/B/C/D	> 95%
W26	HBeAg	116	124	SYVNVNMGL	B/C	80-95%
W71	HBeAg	97	105	LATWVGSNL	B/C	> 95%
W74	HBeAg	170	178	STLPETTVV	A/B/C/D	> 95%
W92	HBeAg	63	71	ASALYREAL	A/B/C/D	A/B/C:>95%; D:80-95%
W94	HBeAg	86	94	QAILCWGEL	B/C/D	B/C:>95%; D:80-95%
W114	HBeAg	38	47	FGASVELLSF	B/C	> 95%
W117	HBeAg	109	117	ASRELVVSY	B/C	80-95%
W162	HBeAg	59	67	LLDTASALY	A/B/C/D	> 95%
W163	HBeAg	105	113	LEDPASREL	B/C	> 95%
W185	HBeAg	131	139	WFHISCLTF	A/B/C/D	> 95%
W210	HBeAg	143	151	TVLEYLVSF	A/B/C	> 95%
W230	HBeAg	81	89	HTALRQAIL	B/C/D	> 95%
W7	HBpol	407	415	FAVPNLQSL	A/B/C/D	> 95%
W8	HBpol	435	444	YHIPLHPAAM	A/C	> 95%
W10	HBpol	539	547	RAFPHCLAF	A/B/C/D	> 95%
W11	HBpol	547	555	FSYMDDVVL	A/B/C/D	> 95%
W12	HBpol	649	657	CGYPALMPL	A/B/C/D	A/B/C:>95%; D:80-95%
W14	HBpol	722	730	PLPIHTAEL	A/B/C/D	> 95%
W15	HBpol	646	654	FTQCGYPAL	A/B/C/D	> 95%
W30	HBpol	3	11	LSYQHFRKL	A/C	> 95%
W31	HBpol	115	123	FYPNLTKYL	C	80-95%
W32	HBpol	328	337	FRNSEPCSEY	B	80-95%
W34	HBpol	483	491	SRNLYVSLL	C/D	80-95%

W36	HBpol	537	545	VRRAFPHCL	A/B/C/D	> 95%
W52	HBpol	62	70	LYSSTVPVF	C	> 95%
W57	HBpol	756	764	KYTSFPWLL	A/B/C/D	> 95%
W75	HBpol	296	304	SSSGHAVEL	B/C	80-95%
W77	HBpol	431	439	SAAFYHIPL	A/C	> 95%
W78	HBpol	529	537	FTSAICSVV	A/B/C/D	> 95%
W97	HBpol	268	276	SASSASSCL	B	80-95%
W100	HBpol	426	434	LSLDVSAAF	A/B/C/D	> 95%
W104	HBpol	598	606	YVIGSWGTL	A/B/C	B/C:80-95%; A: > 95%
W122	HBpol	504	512	YSHPIILGF	A/B/C/D	> 95%
W125	HBpol	642	651	FAAPFTQCGY	A/B/C/D	> 95%
W126	HBpol	667	676	FTFSPTYKAF	A/B/C/D	A/B/C: > 95%; D:80-95%
W127	HBpol	832	840	FASPLHVAW	A/B/C/D	> 95%
W166	HBpol	13	21	LLDDEAGPL	C/D	> 95%
W169	HBpol	440	448	HPAAMPHLL	A/B/C/D	> 95%
W171	HBpol	566	574	LYAAVTNFL	B	80-95%
W173	HBpol	698	706	FADATPTGW	A/C/D	> 95%
W195	HBpol	776	784	SFVYVPSAL	A/B/C/D	> 95%
W233	HBpol	48	56	VSIPWTHKV	A/B/C/D	> 95%
W235	HBpol	450	458	GSSGLPRYV	C	80-95%
W236	HBpol	495	503	KTFGRKLHL	C	> 95%
W239	HBpol	591	599	YSLNFMGYV	A/B/C	C: > 95%; A/B:80-95%
W305	HBpol	587	595	KRWGYSLNF	A/B/C	C: > 95%; A/B:80-95%
W21	HBx	63	71	FSSAGPCAL	A/B/C/D	> 95%
W22	HBx	92	100	VLHKRTLGL	A/B/C	> 95%
W23	HBx	143	151	CSPAPCNFF	B/C	80-95%
W43	HBx	71	79	LRFTSARRM	B/C/D	C:80-95%; B/D: > 95%
W44	HBx	77	85	RRMETTVNA	B/C/D	C:80-95%; B/D: > 95%
W46	HBx	95	103	KRTLGLSAM	B/C	C:80-95%; B: > 95%
W64	HBx	55	63	LRGLPVCAF	A/B/C/D	> 95%
W87	HBx	1	9	MAARVCCQL	B/C	> 95%
W88	HBx	37	45	LPSPSSSAV	C	> 95%
W90	HBx	100	108	LSAMSTTDL	B/C	C:80-95%; B: > 95%
W137	HBx	104	112	STTDLEAYF	A/B/C/D	> 95%
W178	HBx	8	16	QLDPARDVL	B/C/D	C/D:80-95%; B: > 95%
W246	HBx	75	83	SARRMETTV	B/C/D	> 95%
W266	HBx	26	34	RGRPVSGPF	C	> 95%

Table S4 Binding affinities of each HLA-B allotype with relevant peptides as detected by peptide competitive binding assays using 15 transfected HMy2.CIR cell lines expressing indicated HLA-B allotype.

B4601		B4001		B5801		B1502		B5101		B1301		B1302		B1501	
T100	High	T59	High	T84	High	T19	High	T141	High	T1	High	T39	Low	T119	High
T29	High	T35	High	T119	High	T100	High	T144	High	T124	High	T203	Low	T111	High
T165	High	T39	High	T2	High	T33	High	T126	High	T146	Inter	T195	Low	T38	High
T186	High	T33	High	T86	High	T114	High	T136	High	T170	Inter	T162	Low	T207	High
T110	High	T44	High	T14	High	T262	Inter	T122	High	T100	Inter	T199	Low	T8	High
T199	High	T50	High	T71	High	T18	Inter	T127	Inter	T134	Low	T201	Low	T86	High
T33	High	T54	High	T40	High	T111	Inter	T124	Inter	T166	Low	T186	Low	T110	High
T141	High	T55	Inter	T19	High	T8	Inter	T125	Low	T19	Low	T167	Low	T21	High
T24	High	T29	Low	T24	Inter	T70	Inter	T54	Low	T165	Low	T177	Low	T130	High
T2	High	T36	Low	T21	Inter	T21	Inter	T119	Low	T14	Low	T146	Low	T70	High
T21	High	T57	Low	T64	Low	T16	Low	T19	Low	T141	Low	T176	Low	T182	High
T1	High	T176	Low	T70	Low	T1	Low	T134	Low	T61	Low	T124	Low	T220	High
T13	Inter	T195	Low	T69	Low	T14	Low	T120	Low	T110	Low	T115	Low	T166	High
T146	Low	T45	Low	T16	No	T182	Low	T132	Low	T150	Low	T165	Low	T206	Low
T3	Low	T47	Low	T206	No	T110	Low	T177	Low	T40	Low	T45	Low	T127	Low
T25	Low	T52	Low			T101	Low	T14	Low	T35	Low	T182	Low	T18	Low
T124	Low	T27	Low			T115	Low	T130	Low	T47	Low	T47	Low	T126	Low
T248	Low	T127	Low					T116	Low	T69	Low	T36	Low	T125	Low
T14	Low	T252	Low					T262	Low	T176	Low	T19	Low	T228	Low
T150	Low	T119	Low					T143	No	T44	Low	T35	Low	T120	Low
T8	Low	T61	Low							T160	Low	T18	Low	T176	Low
T207	Low	T48	Low							T29	Low			T50	Low
T262	No	T14	No							T162	No			T39	Low
T101	No													T72	Low

T125	No													T19	Low
T115	No													T14	Low
T20	No													T84	Low
T16	No													T1	Low
T47	No													T16	No
T19	No														
B4006		B5401		B3501		B5201		B0702		B4403		B4801			
T260	High	T270	High	T1	High	T162	High	T141	High	T248	High	T260	High		
T252	High	T267	High	T220	High	T176	Inter	T272	Low	T150	High	T207	High		
T59	Inter	T275	Inter	T72	Inter	T127	Low	T275	Low	T47	Inter	T120	High		
T231	Inter	T263	Inter	T272	Inter	T201	Low	T262	Low	T54	Inter	T72	High		
T233	Inter	T261	Inter	T8	Inter	T124	Low	T119	Low	T186	Low	T38	Inter		
T257	Inter	T280	Inter	T262	Low	T47	Low	T19	Low	T50	Low	T84	Inter		
T162	Low	T25	Low	T19	Low	T59	Low	T132	Low	T36	Low	T182	Inter		
T36	Low	T273	Low	T136	Low	T39	Low	T126	Low	T69	Low	T40	Low		
T39	Low	T122	Low	T100	Low	T19	Low	T257	Low	T39	Low	T90	Low		
T52	Low	T141	No	T141	Low	T14	No	T270	No	T206	Low	T261	Low		
T176	Low	T272	No	T16	Low	T203	No			T40	Low	T146	Low		
T186	Low	T116	No	T261	Low					T44	Low	T165	Low		
T47	Low	T125	No	T14	Low					T14	Low	T44	Low		
T248	Low	T262	No	T25	Low							T29	Low		
T44	Low	T136	No									T14	Low		
T45	Low	T19	No									T177	Low		
T50	Low											T124	Low		
T38	Low											T272	Low		
T40	No											T162	Low		
												T86	No		

Table S5 Binding affinities of each HLA-C allotype with relevant peptides as detected by competitive peptide binding assays using 14 transfected HMy2.CIR cell lines expressing indicated HLA-C allotype.

C0102		C0602		C0702		C0801		C0304		C0302		C0303	
W8	High	W30	High	W44	High	W4	High	W94	High	W20	High	W71	High
W4	High	W37	High	W90	High	W19	Inter	W114	High	W10	High	W21	High
W10	High	W34	High	W40	High	W60	Inter	W109	High	W21	High	W74	High
W126	High	W117	High	W38	High	W78	Inter	W97	High	W4	High	W75	High
W2	High	W31	High	W6	Inter	W171	Inter	W71	High	W11	Inter	W15	High
W5	High	W4	High	W20	Inter	W74	Inter	W77	High	W8	Inter	W60	High
W7	High	W21	High	W4	Inter	W5	Inter	W60	Inter	W126	Inter	W4	Low
W20	High	W22	High	W8	Low	W21	Inter	W166	Inter	W5	Inter	W100	Low
W6	Inter	W26	High	W5	Low	W75	Low	W4	Inter	W131	Inter	W11	Low
W14	Inter	W2	Inter	W36	Low	W11	Low	W104	Inter	W127	Inter	W10	Low
W196	Inter	W6	Inter	W2	Low	W83	Low	W92	Inter	W114	Inter	W7	Low
W1	Inter	W7	Inter	W31	Low	W7	Low	W21	Inter	W122	Low	W5	Low
W11	Inter	W36	Inter	W243	Low	W10	Low	W15	Inter	W125	Low	W109	Low
W12	Low	W46	Inter	W34	Low	W77	Low	W87	Inter	W106	Low	W83	Low
W17	Low	W40	Inter	W22	Low	W15	Low	W11	Low	W71	Low	W90	Low
W15	Low	W44	Low	W87	Low	W166	Low	W83	Low	W129	Low	W77	Low
W18	Low	W39	Low	W10	Low	W87	Low	W5	Low	W210	Low	W106	Low
W88	Low	W38	No	W37	Low	W71	Low	W106	Low	W137	Low	W114	Low
W22	Low			W60	Low	W309	Low	W10	Low	W117	Low	W88	Low
W44	Low			W64	No	W44	Low	W75	Low	W87	Low	W87	No
W23	Low			W52	No	W114	Low	W88	Low			W104	No
W40	Low			W57	No	W109	Low					W92	No
W16	Low			W46	No	W85	Low						
W21	Low			W43	No	W88	No						

W19	Low					W92	No						
W230	Low					W90	No						
W39	Low												
C0401		C1402		C1202		C1502		C1403		C1203		C0701	
W176	High	W114	High	W137	High	W90	High	W2	High	W87	High	W4	High
W85	High	W109	High	W20	High	W78	High	W10	High	W21	High	W6	High
W178	High	W31	High	W4	High	W10	High	W6	Low	W60	High	W38	High
W174	High	W52	High	W7	Inter	W21	Inter	W126	Low	W239	High	W8	High
W169	High	W19	Inter	W114	Inter	W30	Inter	W129	Low	W74	Inter	W21	High
W171	High	W20	Inter	W106	Inter	W87	Inter	W196	Low	W122	Inter	W37	High
W166	High	W21	Inter	W78	Low	W239	Inter	W19	Low	W127	Inter	W305	High
W92	Inter	W309	Low	W11	Low	W83	Inter	W46	Low	W4	Inter	W40	High
W137	Inter	W185	Low	W125	Low	W235	Low	W52	Low	W125	Inter	W46	High
W177	Inter	W195	Low	W127	Low	W241	Low	W195	Low	W117	Inter	W44	Inter
W173	Inter	W2	Low	W10	Low	W236	Low	W43	Low	W71	Inter	W32	Inter
W163	Low	W22	Low	W90	Low	W246	Low	W185	Low	W137	Inter	W64	Inter
W31	Low	W43	Low	W71	Low	W233	Low	W30	Low	W243	Inter	W36	Low
W52	Low	W30	Low	W21	Low	W243	Low	W31	No	W246	Low	W309	Low
W15	Low	W46	Low	W122	Low	W75	Low	W20	No	W10	Low	W34	Low
W21	Low	W6	Low	W60	Low			W266	No	W233	Low	W43	Low
W2	Low	W8	Low	W87	Low			W21	No	W286	Low	W60	Low
W162	Low	W171	Low	W210	No			W22	No			W117	Low
W6	Low							W26	No				
W10	Low							W171	No				
W88	Low												
W1	No												

Table S6 Antigen distribution and HLA-A restrictions of 103 T-cell epitopes of HBV antigens used in the ELISpot assay

HLA allotype	Allele frequency	The number of HBV $CD8^+$ T-cell epitopes				Total
		HBsAg	HBpol	HBx	HBeAg (covering HBcAg)	
A1101	22.4%	10	11	7	8	36
A2402	15.3%	11	6	5	9	31
A0201	13.9%	12	14	10	14	50
A0207	9.5%	10	2	1	9	22
A3303	8.1%	5	5	2	2	14
A0206	6.1%	2	7	1	5	15
A3001	5.2%	1	3	3	3	10
A0203	3.9%	5	10	2	6	23
A3101	3.4%	3	1	2	2	8
A0101	2.2%	4	3	1	2	10
A1102	2.3%	7	3	5	4	19
A2601	1.8%	0	2	1	1	4
A0301	1.4%	6	6	5	3	20
Tatol	95.5%	76	73	45	68	262

Table S7 Antigen distribution and HLA-B restrictions of 83 T-cell epitopes of HBV antigens used in the ELISpot assay

HLA allotype	Allele frequency	The number of HBV $CD8^+$ T-cell epitopes				Total
		HBsAg	HBpol	HBx	HBeAg (covering HBcAg)	
B4601	13.32%	8	7	2	4	21
B4001	9.81%	3	6	4	4	17
B5801	7.45%	3	6	1	3	13
B1502	6.43%	4	4	2	4	14
B5101	5.52%	6	7	2	4	19
B1301	5.36%	2	5	1	3	11
B1302	4.92%	1	1	1	3	6
B1501	4.88%	5	7	2	8	22
B4006	3.18%	2	4	0	1	7
B5401	3.03%	4	3	1	2	10
B3501	2.56%	1	4	0	4	9
B5201	2.06%	1	0	1	0	2
B0702	1.89%	2	5	1	0	8
B4403	1.73%	1	3	0	1	5
B4801	1.54%	2	3	0	4	9
Total	73.68%	45	65	18	45	173

Table S8 Antigen distribution and HLA-C restrictions of 54 T-cell epitopes of HBV antigens used in the ELISpot assay

HLA allotype	Allele frequency	The number of HBV CD8^{+} T-cell epitopes				Total
		HBsAg	HBpol	HBx	HBeAg (covering HBcAg)	
C0102	15.38%	4	4	1	3	12
C0602	13.56%	2	4	2	3	11
C0702	12.75%	3	1	4	4	12
C0801	8.57%	2	1	1	3	7
C0304	8.31%	1	3	1	4	9
C0302	6.96%	1	1	2	1	5
C0303	6.94%	4	3	1	2	10
C0401	6.52%	4	3	1	2	10
C1402	4.49%	1	2	1	2	6
C1202	3.61%	2	0	1	1	4
C1502	2.57%	0	2	1	0	3
C1403	2.16%	0	1	0	1	2
C1203	1.30%	1	0	3	0	4
C0701	1.22%	2	2	1	2	7
Total	94.34%	27	27	20	28	102

Table S9 39 sequence-identical epitopes cross-restricted by HLA-A, B, and C molecules

Protein	Peptide	HLA-A restriction	HLA-B restriction	HLA-C restriction
HBsAg	P95/T176	A2402/A0203	B1501/B5201	
	P72/P108/<br>T90/W19	P108:A1102/A0101/A0301<br>P72:A0301/A1102/A0101/A0207/A0201/A2402	B1502/B1302	C0303/C1202
	P141/W177	A0201		C0401
	P71/P107	P71: A0207/A0101<br>P107: A0301/A1101		
	W106/T2		B4601/B5801	C0303
	P40/P103	P103:A1101/A2402/A0207/A1102<br>P40:A1101		
	P77/P59	P77:A0201/A3303<br>P59:A3303 A3101		
HBpol	P134/T165	A0201/A0203	B4601	
	P120/T297	A2601	B0702	
	P105/T115	A0301/A6801/A1102/A1101	B1502/1302/4601	
	P68/T111	A0201/A0206/A0203	B1502/B1501	

	T19/W10		B5801/B1502/B1302	C0102/C0302/C1502/C1403
	W126/T114		B1502	C0102
	P130/T292	A0101/A2902/A3002	B5801	
	P112/T21	A2902/A3002	B4601/B5801/B1502/B1501	
	W7/T20		B4601	C0102/C0303
	W52/P56	A2402/A0201/A0207		C1402
	W169/T14		B4601/B5101/B1301	C0401
HBx	P84/T14/W21	A2402	B4001/B5801/B5101	C0602/C0302/C1203
	P94/P35/W22	P94:A0201/A3001<br>P35:A0203/A0201/A0207		C0602
	T162/P70/W178	A0201	B5201	C0401
	P18/P121/W137	P18:A1101/A0201<br>P121: A1101/A1102/A2601		C1202
	T220/P26	A2402	B1502/B1501	
HBeAg	P66/ T124	A0201/A0206	B4601/B5101/B1301	
	P87/W6	A0201/A3001		C0702/C0701
	P52/T100	A2402/A0201/A0207	B4601/B1502/B1301	
	T13/W114		B4601	C0801/C0304/C1402
	P31/T127	A1101/A2402/A0201/A0207/A0203	B4001/B5101/B1501	
	P127/W4	A0101/A2902/A3002		C0102/C0602/C0702/C0801/C0302/C1202/C0701
	P88/W117	A0201/A0206/A3001		C0602
	P53/W26/T72	A2402/A0207	B5801/B1501/B3501/B4801	C0602/C0702/C0801/C0401/C1402
	P42/W74	A1101/A0201/A0207/A0206/A0203/A0301/A0202/A6801/6802		C0303

Table S10 Intra-assay and inter-assay CV values of SFUs in the in-house ELISpot assay

	Patient	First test (SFUs)	Second test (SFUs)	Third test (SFUs)	Mean (SFUs)	SD	CV (%)	Mean CV (%)
Intra-assay	Patient 1	342	305	323	323	13.08	4.05%	7.22%
	Patient 2	145	156	165	155	7.08	4.56%	
	Patient 3	57	40	45	47	6.17	13.05%	
	Patient 4	87	92	100	93	4.63	4.99%	
	Patient 5	123	155	158	145	13.71	9.44%	
Inter-assay	Patient 6	208	204	212	208	3.26	1.57%	7.84%
	Patient 7	33	40	45	39	4.92	12.51%	
	Patient 8	157	198	177	177	16.73	9.44%	
	Patient 9	456	400	389	415	29.33	7.07%	
	Patient 10	77	89	95	87	7.48	8.60%	

Table S11 Characteristics of 81 CHB patients and 10 HBV-infected LC patients detected by peptide pool

Patients in the cohort	CHB (n = 81)	LC (n = 10)	P value
Age(years)	65 (33-88)	65 (57-83)	0.227
Gender (Male/Female)	55/26	6/4	
HBV DNA (IU/ml)	<500 (<2-170000000)	<2 (<2-650000)	0.859
HBsAg (IU/mL)	186.9 (0.17-2224.1)	388.5 (44.56-3899.3)	**0.024**
HBeAg (COI)	0.38 (0.07-600.1)	8.26 (0.37-299.62)	**0.022**
HBsAb (IU/ml)	0.27 (0-95.66)	0 (0-56.04)	0.196
HBcAb (COI)	7.495 (3.09-9.44)	6.59 (5.85-7.92)	0.102
HBeAb (COI)	2.675 (0.01-50.55)	2.135 (0.01-16.56)	0.080
ALT (U/L)	28.7 (8.5-123.8)	38.65 (16.1-85.5)	0.875
NUCs treatment (TMF/ TDF/ TAF/ ETV)	25 (10/8/3/4)	10 (0/1/4/5)	
IFN treatment	38	0	
NUCs / IFN treatment	18	0	

Note: TMF: Tenofovir Amibufenamide, 25 mg QD; TDF: Tenofovir Disoproxil Fumarate, 300 mg QD; TAF: Tenofovir alafenamide Fumarate, 25 mg QD; ETV: Entecavir, 25 mg QD; IFN: PEG-interferon-α, 180 μg QW

```
                           10        20        30        40        50        60        70        80
                  ....|....|....|....|....|....|....|....|....|....|....|....|....|....|....|....|
HBsAg-C(P31868)   MGGWSSKPRQGMGTNLSVPNPLGFFPDHQLDPAFGANSNNPDWDFNPNKDHWPEANQVGVGTFGPGFTPPHGGLLGWSPQ
HBsAg-A(P31873)   .....A...K...................................................A................S.
HBsAg-B(Q67926)   .........K........................K...E.....L..H..N..D..K....A..................
HBsAg-D(P03139)   -----------..Q...TS...............R..T............T..D..K..A.A..L...............

                           90       100       110       120       130       140       150       160
                  ....|....|....|....|....|....|....|....|....|....|....|....|....|....|....|....|
HBsAg-C(P31868)   AQGILTTVPAAPPPASTNRQSGRQPTPISPPLRDSHPQAMQWNSTTFHQALLDPRVRGLYFPAGGSSSGTVNPVPTTASP
HBsAg-A(P31873)   ...T.H....V.........T........................A.Q...Q.......F.............A.NI..H
HBsAg-B(Q67926)   ...L.......................F......T............L.T.Q.S...A..L..........S.AQN.V.A
HBsAg-D(P03139)   ....MQ.L..N................L.....TT.....H........T.Q.........................T..

                          170       180       190       200       210       220       230       240
                  ....|....|....|....|....|....|....|....|....|....|....|....|....|....|....|....|
HBsAg-C(P31868)   ISSIFSRTGDPAPNMENTTSGFLGPLLVLQAGFFLLTRILTIPQSLDSWWTSLNFLGGAPTCPGQNSQSPTSNHSPTSCP
HBsAg-A(P31873)   ....S.......L....I........................................S.V.L.................
HBsAg-B(Q67926)   ....S.K....V.....IA..L..H.........S..K....................T.A........QI.S....C..
HBsAg-D(P03139)   .......I....L....I........................................TTV.L.......I.........

                          250       260       270       280       290       300       310       320
                  ....|....|....|....|....|....|....|....|....|....|....|....|....|....|....|....|
HBsAg-C(P31868)   PICPGYRWMCLRRFIIFLFILLLCLIFLLVLLDYQGMLPVCPLLPGTSTTSTGPCKTCTTPAQGTSMFPSCCCTKPSDGN
HBsAg-A(P31873)   ...........................................I..ST................N...........T...
HBsAg-B(Q67926)   ..................C........................T..ST............................T...
HBsAg-D(P03139)   .T.........................................I..S......S.R........I..Y............

                          330       340       350       360       370       380       390       400
                  ....|....|....|....|....|....|....|....|....|....|....|....|....|....|....|....|
HBsAg-C(P31868)   CTCIPIPSSWAFARFLWEWASVRFSWLSLLVPFVQWFAGLSPTVWLSVIWMMWYWGPSLYNILSPFLPLLPIFFCLWVYI
HBsAg-A(P31873)   .............KY......................V............................I.............
HBsAg-B(Q67926)   .............KY..G...................V...............F.........R..M....T........
HBsAg-D(P03139)   ............GK.......A...............V....I.................S................A..


                           10        20        30        40        50        60        70        80
                  ....|....|....|....|....|....|....|....|....|....|....|....|....|....|....|....|
HBeAg-C(P0C6H5)   MQLFHLCLIISCSCPTVQASKLCLGWLWGMDIDPYKEFGASVELLSFLPSDFFPSIRDLLDTASALYREALESPEHCSPH
HBeAg-A(Q91C37)   ............T...........................T..............V........................
HBeAg-B(P0C6G7)   ................................................................................
HBeAg-D(P0C573)   ........................................T..............V........................

                           90       100       110       120       130       140       150       160
                  ....|....|....|....|....|....|....|....|....|....|....|....|....|....|....|....|
HBeAg-C(P0C6H5)   HTALRQAILCWGELMNLATWVGSNLEDPASRELVVSYVNVNMGLKIRQLLWFHISCLTFGRETVLEYLVSFGVWIRTPPA
HBeAg-A(Q91C37)   .....ET........T......N........D...N...T........................................
HBeAg-B(P0C6G7)   ................................................................................
HBeAg-D(P0C573)   ...............T......V........D.......T.....F..................I...............

                          170       180       190       200       210
                  ....|....|....|....|....|....|....|....|....|....|....
HBeAg-C(P0C6H5)   YRPPNAPILSTLPETTVVRRRGRSPRRRTPSPRRRRSQSPRRRRSQSRESQC
HBeAg-A(Q91C37)   .....................D.GRSP.RRT.SP..RR.QSP..RR.QSRESQC
HBeAg-B(P0C6G7)   ....................................................
HBeAg-D(P0C573)   ....................................................
```

```
                           10        20        30        40        50        60        70        80
                   ....|....|....|....|....|....|....|....|....|....|....|....|....|....|....|....|
HBpol-C(P0C688)    MPLSYQHFRKLLLLDDE--AGPLEEELPRLADEGLNRRVAEDLNLGNLNVSIPWTHKVGNFTGLYSSTVPVFNPEWQTPS
HBpol-A(Q02314)    .................TE.............AD....................................I.........
HBpol-B(Q67925)    ..........M....E.--...........................D.......................C...Q.....
HBpol-D(Q9QMI1)    .........R.......--................................................S......H.K..T

                           90       100       110       120       130       140       150       160
                   ....|....|....|....|....|....|....|....|....|....|....|....|....|....|....|....|
HBpol-C(P0C688)    FPNIHLQEDIINRCQQYVGPLTVNEKRRLKLIMPARFYPNLTKYLPLDKGIKPYYPEHAVNHYFKTRHYLHTLWKAGILY
HBpol-A(Q02314)    ..K...H...A.....F.......................S...........T...D.V.....Q...............
HBpol-B(Q67925)    ..S.......VD..K.F........N..............V................YI.D...Q...............
HBpol-D(Q9QMI1)    ......HQ....K.E.F............Q..........F.................L.....Q...............

                          170       180       190       200       210       220       230       240
                   ....|....|....|....|....|....|....|....|....|....|....|....|....|....|....|....|
HBpol-C(P0C688)    KRETTRSASFCGSPYSWEQELQHGRLVFQTSTRHGDESFCSQSSGILSRSPVGPCIRSQLKQSRLGLQPQQGSLARGKSG
HBpol-A(Q02314)    .....................H.....IK..Q.....P....P.......S........F.........H..P..TSQP.
HBpol-B(Q67925)    ...S...............D...........K....K...P..P...P..S.....Q...RK....P..A..Q..GRQQ.
HBpol-D(Q9QMI1)    .....H.............K....-----------A...HQ..P.....P...SSLQ.KHQK......S...H...RQQ.

                          250       260       270       280       290       300       310       320
                   ....|....|....|....|....|....|....|....|....|....|....|....|....|....|....|....|
HBpol-C(P0C688)    RSGSIWARVHSTTRRSFGVEPSGSGHIDNSASSASSCLYQSAVRKTAYSHLSTSKRQSSSGHAVELHNIPPSCARSQSEG
HBpol-A(Q02314)    ...........P...C...........GHR..D.....H......A...................F.SF...S.....Q.
HBpol-B(Q67925)    G...R.....PSPWGTV........PTH.C...S....H......A..L.....GH...........F..NSS.F..Q.
HBpol-D(Q9QMI1)    ..W.R.....P.A..P.....A....TT.F..KSA..S...P...A..PTV.....R.......DF..L...S......R

                          330       340       350       360       370       380       390       400
                   ....|....|....|....|....|....|....|....|....|....|....|....|....|....|....|....|
HBpol-C(P0C688)    PISSCWWLQFRNSEPCSDYCLTHIVNLLEDWGPCTEHGEHNIRIPRTPARVTGGVFLVDKNPHNTTESRLVVDFSQFSRG
HBpol-A(Q02314)    .VF.........TQ...N...S.L................H........................A..............
HBpol-B(Q67925)    .VP..............E...S.....I............R..T....................................
HBpol-D(Q9QMI1)    .VFP.........K.......S..................H........................A..............

                          410       420       430       440       450       460       470       480
                   ....|....|....|....|....|....|....|....|....|....|....|....|....|....|....|....|
HBpol-C(P0C688)    STHVSWPKFAVPNLQSLTNLLSSNLSWLSLDVSAAFYHIPLHPAAMPHLLVGSSGLPRYVARLSSTSRNINYQHGTMQDL
HBpol-A(Q02314)    ..R...............................................I.....S........N..IN.N....L.N.
HBpol-B(Q67925)    N.R...................................L.................S........H..IN.N......N.
HBpol-D(Q9QMI1)    NYR...................................L.................S........N..IFDH......N.

                          490       500       510       520       530       540       550       560
                   ....|....|....|....|....|....|....|....|....|....|....|....|....|....|....|....|
HBpol-C(P0C688)    HDSCSRNLYVSLLLLYKTFGRKLHLYSHPIILGFRKIPMGVGLSPFLLAQFTSAICSVVRRAFPHCLAFSYMDDVVLGAK
HBpol-A(Q02314)    ......Q.....M.....Y.W...........................................................
HBpol-B(Q67925)    .N..........M.....Y.............................................................
HBpol-D(Q9QMI1)    ..Y.............Q...............................................................

                          570       580       590       600       610       620       630       640
                   ....|....|....|....|....|....|....|....|....|....|....|....|....|....|....|....|
HBpol-C(P0C688)    SVQHLESLFTSITNFLLSLGIHLNPHKTKRWGYSLNFMGYVIGSWGTLPQEHIVLKIKQCFRKLPVNRPIDWKVCQRIVG
HBpol-A(Q02314)    ........Y.AV.............N........................D...Q...H..................L..
HBpol-B(Q67925)    ......A.YAAV..........................................Q...M.....................
HBpol-D(Q9QMI1)    ..........AV.............N.........H..........S...D...H.L.E.....................

                          650       660       670       680       690       700       710       720
                   ....|....|....|....|....|....|....|....|....|....|....|....|....|....|....|....|
HBpol-C(P0C688)    LLGFAAPFTQCGYPALMPLYACIQSKQAFTFSPTYKAFLCKQYLHLYPVARQRSGLCQVFADATPTGWGLAIGQSGMRGT
HBpol-A(Q02314)    ........................A..............S...MN........P...................HQR..E.
HBpol-B(Q67925)    ........................A..............S....N........P......R............HQR....
HBpol-D(Q9QMI1)    .......................................Y...MN..........................M.HQR....

                          730       740       750       760       770       780       790       800
                   ....|....|....|....|....|....|....|....|....|....|....|....|....|....|....|....|
HBpol-C(P0C688)    FVAPLPIHTAELLAACFARSRSGAKLIGTDNSVVLSRKYTSFPWLLGCAANWILRGTSFVYVPSALNPADDPSRGRLGLY
HBpol-A(Q02314)    ....................................Q...........T...............................
HBpol-B(Q67925)    ..S..........................H..................................................
HBpol-D(Q9QMI1)    .Q......................NIL....................................................C

                          810       820       830       840
                   ....|....|....|....|....|....|....|....|....|
HBpol-C(P0C688)    RPLLHLPFRPTTGRASLYAVSPSVPSHLPVRVHFASPLHVAWRPP
HBpol-A(Q02314)    ....R..Y......T..............................
HBpol-B(Q67925)    ....R..YQ.....T....D.........D...............
HBpol-D(Q9QMI1)    ....R.........T..............DH..............
```

```
                           10        20        30        40        50        60        70        80
                   ....|....|....|....|....|....|....|....|....|....|....|....|....|....|....|....|
HBx-C(P0C686)      MAARVCCQLDPARDVLCLRPVGAESRGRPVSGPFGPLPSPSSSAVPADHGARLSLRGLPVCAFSSAGPCALRFTSARRME
HBx-A(Q91C38)      ....LY....SS.................LA..L.A.S...P....S....H.........................C..
HBx-B(Q67923)      .............................LP..L.T..PA.PP...T....H............................
HBx-D(Q9QMI3)      ....L............................L.S.S.S.P....T....H............................

                           90       100       110       120       130       140       150
                   ....|....|....|....|....|....|....|....|....|....|....|....|....|....|....
HBx-C(P0C686)      TTVNAHQVLPKVLHKRTLGLSAMSTTDLEAYFKDCLFKDWEELGEEIRLMVFVLGGCRHKLVCSPAPCNFFTSA
HBx-A(Q91C38)      .......I............P..............V.............K............FA.SS.......
HBx-B(Q67923)      ......GN...........................V.NE..........K........................
HBx-D(Q9QMI3)      .......I...I.........T...........................K.............A..........
```

FIGURE S1 | Homologous analysis of HBsAg (covering pre-S1, pre-S2 and S), HBeAg (containing HBcAg), HBx and HBpol proteins from HBV C, A, B, and D genotypes. The entire amino acid sequences of each protein from different genotypes of HBV were obtained from the UniProt database, aligned and used for *in silico* prediction of T-cell epitopes presented by HLA-B and HLA-C allotypes.

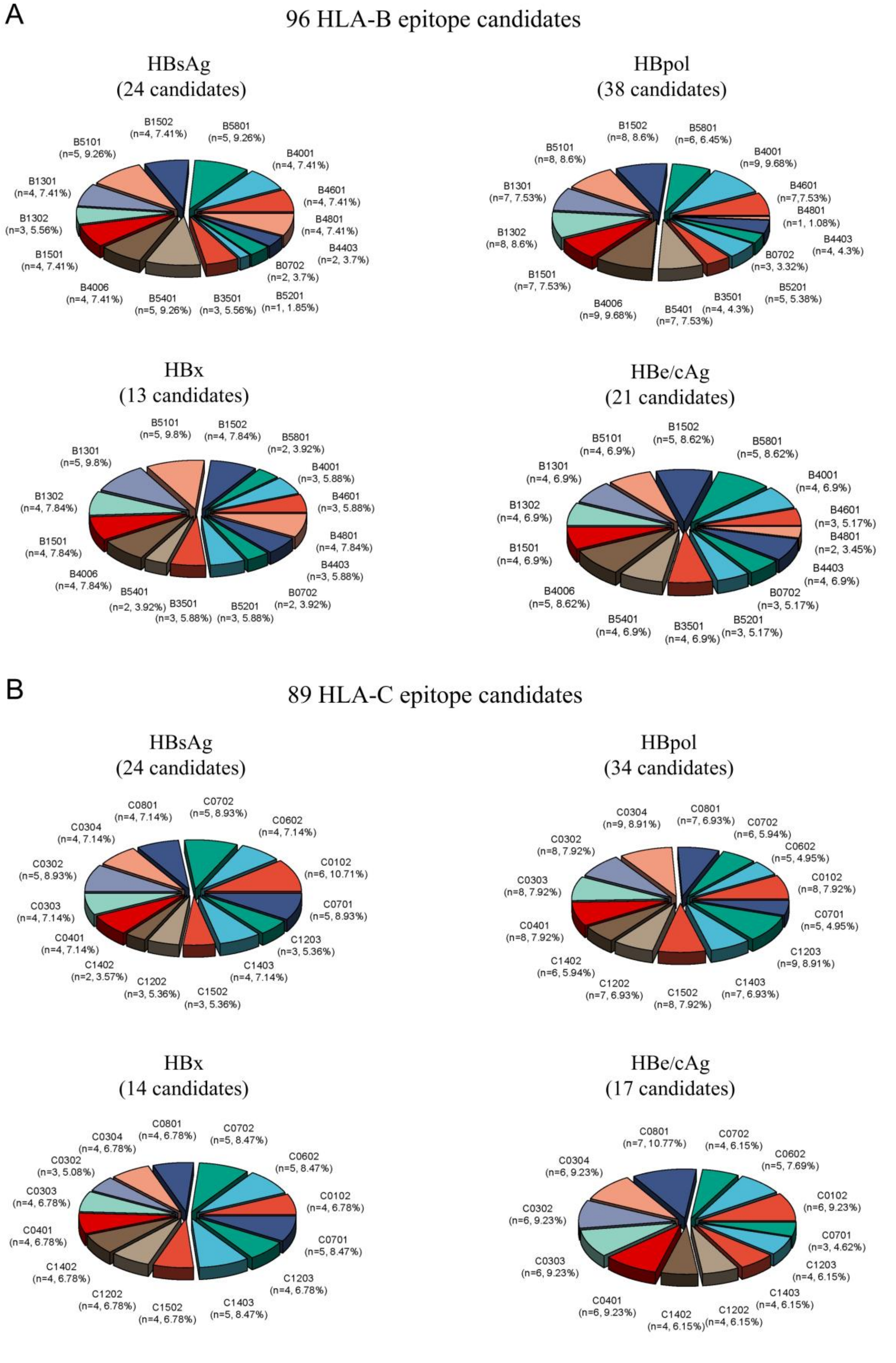

FIGURE S2 | Prediction and selection of candidate T-cell epitopes derived from HBV main

proteins and restricted by 15 prevalent HLA-B and 14 prevalent HLA-C allotypes. (A) The numbers of candidate epitopes harboring in each protein and restricted by each HLA-B allotype. (B) The numbers of candidate epitopes harboring in each protein and restricted by each HLA-C allotype.

HBV C genotype-HBsAg

sp|P31868|:

MGGWSSKPRQGMGTNLSVPNPLGFFPDHQLDPAFGANSNNPDWDFNPNKDHWPEANQVGVGT
FGPGFTPPHGGLLGWSPQAQGILTTVPAAPPPASTNRQSGRQPTPISPPLRDSHPQAMQWNS
TTFHQALLDPRVRGLYFPAGGSSSGTVNPVPTTASPISSIFSRTGDPAPNMENTTSGFLGPL
LVLQAGFFLLTRILTIPQSLDSWWTSLNFLGGAPTCPGQNSQSPTSNHSPTSCPPICPGYRW
MCLRRFIIFLFILLLCLIFLLVLLDYQGMLPVCPLLPGTSTTSTGPCKTCTTPAQGTSMFPS
CCCTKPSDGNCTCIPIPSSWAFARFLWEWASVRFSWLSLLVPFVQWFAGLSPTVWLSVIWMM
WYWGPSLYNILSPFLPLLPIFFCLWVYI

HBV C genotype-HBeAg

sp|P0C6H5|:

SKLCLGWLWGMDIDPYKEFGASVELLSFLPSDFFPSIRDLLDTASALYREALESPEHCSPHH
TALRQAILCWGELMNLATWVGSNLEDPASRELVVSYVNVNMGLKIRQLLWFHISCLTFGRET
VLEYLVSFGVW

HBV C genotype-HBx

sp|P0C686|:

MAARVCCQLDPARDVLCLRPVGAESRGRPVSGPFGPLPSPSSSAVPADHGARLSLRGLPVCA
FSSAGPCALRFTSARRMETTVNAHQVLPKVLHKRTLGLSAMSTTDLEAYFKDCLFKDWEELG
EEIRLMVFVLGGCRHKLVCSPAPCNFFTSA

HBV C genotype-HBpol

sp|P0C688|:

MPLSYQHFRKLLLLDDEAGPLEEELPRLADEGLNRRVAEDLNLGNLNVSIPWTHKVGNFTGL
YSSTVPVFNPEWQTPSFPNIHLQEDIINRCQQYVGPLTVNEKRRLKLIMPARFYPNLTKYLP
LDKGIKPYYPEHAVNHYFKTRHYLHTLWKAGILYKRETTRSASFCGSPYSWEQELQHGRLVF
QTSTRHGDESFCSQSSGILSRSPVGPCIRSQLKQSRLGLQPQQGSLARGKSGRSGSIWARVH
STTRRSFGVEPSGSGHIDNSASSASSCLYQSAVRKTAYSHLSTSKRQSSSGHAVELHNIPPS
CARSQSEGPISSCWWLQFRNSEPCSDYCLTHIVNLLEDWGPCTEHGEHNIRIPRTPARVTGG
VFLVDKNPHNTTESRLVVDFSQFSRGSTHVSWPKFAVPNLQSLTNLLSSNLSWLSLDVSAAF
YHIPLHPAAMPHLLVGSSGLPRYVARLSSTSRNINYQHGTMQDLHDSCSRNLYVSLLLLYKT
FGRKLHLYSHPIILGFRKIPMGVGLSPFLLAQFTSAICSVVRRAFPHCLAFSYMDDVVLGAK
SVQHLESLFTSITNFLLSLGIHLNPHKTKRWGYSLNFMGYVIGSWGTLPQEHIVLKIKQCFR

KLPVNRPIDWKVCQRIVGLLGFAAPFTQCGYPALMPLYACIQSKQAFTFSPTYKAFLCKQYL
HLYPVARQRSGLCQVFADATPTGWGLAIGQSGMRGTFVAPLPIHTAELLAACFARSRSGAKL
IGTDNSVVLSRKYTSFPWLLGCAANWILRGTSFVYVPSALNPADDPSRGRLGLYRPLLHLPF
RPTTGRASLYAVSPSVPSHLPVRVHFASPLHVAWRPP

HBV B genotype-HBsAg

sp|Q67926|

MGGWSSKPRKGMGTNLSVPNPLGFFPDHQLDPAFKANSENPDWDLNPHKDNWPDANKVGVGA
FGPGFTPPHGGLLGWSPQAQGLLTTVPAAPPPASTNRQSGRQPTPFSPPLRDTHPQAMQWNS
TTFLQTLQDSRVRALYLPAGGSSSGTVSPAQNTVSAISSISSKTGDPVPNMENIASGLLGHL
LVLQAGFFSLTKILTIPQSLDSWWTSLNFLGGTPACPGQNSQSQISSHSPTCCPPICPGYRW
MCLRRFIIFLCILLLCLIFLLVLLDYQGMLPVCPLTPGSTTTSTGPCKTCTTPAQGTSMFPS
CCCTKPTDGNCTCIPIPSSWAFAKYLWGWASVRFSWLSLLVPFVQWFVGLSPTVWLSVIWMM
WFWGPSLYNILRPFMPLLPTFFCLWVYI

HBV B genotype-HBeAg

sp|P0C6G7|

SKLCLGWLWGMDIDPYKEFGASVELLSFLPSDFFPSIRDLLDTASALYREALESPEHCSPHH
TALRQAILCWGELMNLATWVGSNLEDPASRELVVSYVNVNMGLKIRQLLWFHISCLTFGRET
VLEYLVSFGVW

HBV B genotype-HBx

sp|Q67923|

MAARVCCQLDPARDVLCLRPVGAESRGRPLPGPLGTLPPASPPAVPTDHGAHLSLRGLPVCA
FSSAGPCALRFTSARRMETTVNAHGNLPKVLHKRTLGLSAMSTTDLEAYFKDCVFNEWEELG
EEIRLKVF VLGGCRHKLVCSPAPCNFFTSA

HBV B genotype-HBpol

sp|Q67925| :

MPLSYQHFRKMLLLDEEAGPLEEELPRLADEGLNRRVAEDLNLGDLNVSIPWTHKVGNFTGL
YSSTVPCFNPQWQTPSFPSIHLQEDIVDRCKQFVGPLTVNENRRLKLIMPARFYPNVTKYLP
LDKGIKPYYPEYIVDHYFQTRHYLHTLWKAGILYKRESTRSASFCGSPYSWEQDLQHGRLVF
QTSKRHGDKSFCPQSPGILPRSSVGPCIQSQLRKSRLGPQPAQGQLAGRQQGGSGSIRARVH
PSPWGTVGVEPSGSGTHNCASSSSSCLHQSAVRKAAYSLLSTSKGHSSSGHAVELHNFPPNS
SRFQSQGPVPSCWWLQFRNSEPCSEYCLSHIVNLIEDWGPCTEHGEHRIRTPRTPARVTGGV
FLVDKNPHNTTESRLVVDFSQFSRGNTRVSWPKFAVPNLQSLTNLLSSNLSWLSLDVSAAFY
HLPLHPAAMPHLLVGSSGLSRYVARLSSHSRINNNQHGTMQNLHNSCSRNLYVSLMLLYKTY
GRKLHLYSHPIILGFRKIPMGVGLSPFLLAQFTSAICSVVRRAFPHCLAFSYMDDVVLGAKS
VQHLEALYAAVTNFLLSLGIHLNPHKTKRWGYSLNFMGYVIGSWGTLPQEHIVQKIKMCFRK
LPVNRPIDWKVCQRIVGLLGFAAPFTQCGYPALMPLYACIQAKQAFTFSPTYKAFLSKQYLN
LYPVARQRPGLCQVFRDATPTGWGLAIGHQRMRGTFVSPLPIHTAELLAACFARSRSGAKLI
GTHNSVVLSRKYTSFPWLLGCAANWILRGTSFVYVPSALNPADDPSRGRLGLYRPLLRLPYQ

PTTGRTSLYADSPSVPSHLPDRVHFASPLHVAWRPP

HBV A genotype-HBsAg

sp|P31873| :

MGGWSAKPRKGMGTNLSVPNPLGFFPDHQLDPAFGANSNNPDWDFNPNKDHWPEANQVGVGA
FGPGFTPPHGGLLGWSSQAQGTLHTVPAVPPPASTNRQTGRQPTPISPPLRDSHPQAMQWNS
TAFQQALQDPRVRGLFFPAGGSSSGTVNPAPNIASHISSISSRTGDPALNMENITSGFLGPL
LVLQAGFFLLTRILTIPQSLDSWWTSLNFLGGSPVCLGQNSQSPTSNHSPTSCPPICPGYRW
MCLRRFIIFLFILLLCLIFLLVLLDYQGMLPVCPLIPGSTTTSTGPCKTCTTPAQGNSMFPS
CCCTKPTDGNCTCIPIPSSWAFAKYLWEWASVRFSWLSLLVPFVQWFVGLSPTVWLSVIWMM
WYWGPSLYNILSPFIPLLPIFFCLWVYI

HBV A genotype-HBeAg

sp|Q91C37| :

SKLCLGWLWGMDIDPYKEFGATVELLSFLPSDFFPSVRDLLDTASALYREALESPEHCSPHH
TALRETILCWGELMTLATWVGNNLEDPASRDLVVNYVNTNMGLKIRQLLWFHISCLTFGRET
VLEYLVSFGVW

HBV A genotype-HBx

sp|Q91C38| :

MAARLYCQLDSSRDVLCLRPVGAESRGRPLAGPLGALSSPSPSAVPSDHGAHLSLRGLPVCA
FSSAGPCALRFTSARCMETTVNAHQILPKVLHKRTLGLPAMSTTDLEAYFKDCVFKDWEELG
EEIRLKVFVLGGCRHKLVFAPSSCNFFTSA

HBV A genotype-HBpol

sp|Q02314| :

MPLSYQHFRKLLLLDDETEAGPLEEELPRLADADLNRRVAEDLNLGNLNVSIPWTHKVGNFT
GLYSSTVPIFNPEWQTPSFPKIHLHEDIANRCQQFVGPLTVNEKRRLKLIMPARFYPNSTKY
LPLDKGIKTYYPDHVVNHYFQTRHYLHTLWKAGILYKRETTRSASFCGSPYSWEQELHHGRL
VIKTSQRHGDEPFCSQPSGILSRSSVGPCIRSQFKQSRLGLQPHQGPLATSQPGRSGSIWAR
VHSPTRRCFGVEPSGSGHIGHRASDASSCLHQSAVRKAAYSHLSTSKRQSSSGHAVEFHSFP
PSSARSQSQGPVFSCWWLQFRNTQPCSNYCLSHLVNLLEDWGPCTEHGEHHIRIPRTPARVT
GGVFLVDKNPHNTAESRLVVDFSQFSRGSTRVSWPKFAVPNLQSLTNLLSSNLSWLSLDVSA
AFYHIPLHPAAMPHLLIGSSGLSRYVARLSSNSRINNNQHGTLQNLHDSCSRQLYVSLMLLY
KTYGWKLHLYSHPIILGFRKIPMGVGLSPFLLAQFTSAICSVVRRAFPHCLAFSYMDDVVLG
AKSVQHLESLYTAVTNFLLSLGIHLNPNKTKRWGYSLNFMGYVIGSWGTLPQDHIVQKIKHC
FRKLPVNRPIDWKVCQRLVGLLGFAAPFTQCGYPALMPLYACIQAKQAFTFSPTYKAFLSKQ
YMNLYPVARQRPGLCQVFADATPTGWGLAIGHQRMRETFVAPLPIHTAELLAACFARSRSGA
KLIGTDNSVVLSQKYTSFPWLLGCTANWILRGTSFVYVPSALNPADDPSRGRLGLYRPLLRL
PYRPTTGRTSLYAVSPSVPSHLPVRVHFASPLHVAWRPP

HBV D genotype-HBsAg

sp|P03139| :

MGQNLSTSNPLGFFPDHQLDPAFRANTNNPDWDFNPNKDTWPDANKVGAGAFGLGFTPPHGG
LLGWSPQAQGIMQTLPANPPPASTNRQSGRQPTPLSPPLRTTHPQAMHWNSTTFHQTLQDPR
VRGLYFPAGGSSSGTVNPVPTTTSPISSIFSRIGDPALNMENITSGFLGPLLVLQAGFFLLT
RILTIPQSLDSWWTSLNFLGGTTVCLGQNSQSPISNHSPTSCPPTCPGYRWMCLRRFIIFLF
ILLLCLIFLLVLLDYQGMLPVCPLIPGSSTTSTGSCRTCTTPAQGISMYPSCCCTKPSDGNC
TCIPIPSSWAFGKFLWEWASARFSWLSLLVPFVQWFVGLSPIVWLSVIWMMWYWGPSLYSIL
SPFLPLLPIFFCLWAYI

HBV D genotype-HBeAg

sp|P0C573| :

SKLCLGWLWGMDIDPYKEFGATVELLSFLPSDFFPSVRDLLDTASALYREALESPEHCSPHH
TALRQAILCWGELMTLATWVGVNLEDPASRDLVVSYVNTNMGLKFRQLLWFHISCLTFGRET
VIEYLVSFGVW

HBV D genoype-HBx

sp|Q9QMI3| :

MAARLCCQLDPARDVLCLRPVGAESRGRPVSGPLGSLSSSSPSAVPTDHGAHLSLRGLPVCA
FSSAGPCALRFTSARRMETTVNAHQILPKILHKRTLGLSTMSTTDLEAYFKDCLFKDWEELG
EEIRLKVFVLGGCRHKLVCAPAPCNFFTSA

HBV D genotype-HBpol

sp|Q9QMI1|

MPLSYQHFRRLLLLDDEAGPLEEELPRLADEGLNRRVAEDLNLGNLNVSIPWTHKVGNFTGL
YSSSVPVFNPHWKTPTFPNIHLHQDIINKCEQFVGPLTVNEKRRLQLIMPARFYPNFTKYLP
LDKGIKPYYPEHLVNHYFQTRHYLHTLWKAGILYKRETTHSASFCGSPYSWEQKLQHGAESF
HQQSPGILSRPPVGSSLQSKHQKSRLGLQSQQGHLARRQQGRSWSIRARVHPTARRPFGVEP
AGSGHTTNFASKSASCSYQSPVRKAAYPTVSTSKRRSSSGHAVDFHNLPPSSARSQSERPVF
PCWWLQFRNSKPCSDYCLSHIVNLLEDWGPCTEHGEHHIRIPRTPARVTGGVFLVDKNPHNT
AESRLVVDFSQFSRGNYRVSWPKFAVPNLQSLTNLLSSNLSWLSLDVSAAFYHLPLHPAAMP
HLLVGSSGLSRYVARLSSNSRIFDHQHGTMQNLHDYCSRNLYVSLLLLYQTFGRKLHLYSHP
IILGFRKIPMGVGLSPFLLAQFTSAICSVVRRAFPHCLAFSYMDDVVLGAKSVQHLESLFTA
VTNFLLSLGIHLNPNKTKRWGYSLHFMGYVIGSWGSLPQDHIVHKLKECFRKLPVNRPIDWK
VCQRIVGLLGFAAPFTQCGYPALMPLYACIQSKQAFTFSPTYKAFLYKQYMNLYPVARQRSG
LCQVFADATPTGWGLAMGHQRMRGTFQAPLPIHTAELLAACFARSRSGANILGTDNSVVLSR
KYTSFPWLLGCAANWILRGTSFVYVPSALNPADDPSRGRLGLCRPLLRLPFRPTTGRTSLYA
VSPSVPSHLPDHVHFASPLHVAWRPP

FIGURE S3 | Consensus sequences of each amino acid in HBsAg (covering pre-S1, pre-S2 and S), HBeAg (containing HBcAg), HBx and HBpol across different HBV genotypes. A huge number of sequences of HBsAg, HBeAg, HBx and HBpol in HBV genotype A, B, C and D were collected from HBVdb database. Multiple sequence alignments were performed and analyzed to obtain the conservative regions of these sequences in a genotype-dependent

way. The conservative properties of each amino acid in each protein were judged by the threshold of 100%, 95%, 80%, and highlighted the amino acids in different colors (red: 100%; yellow: ≥ 95%; black in gray backgrouund: ≥ 80%; black in white background : < 80%).

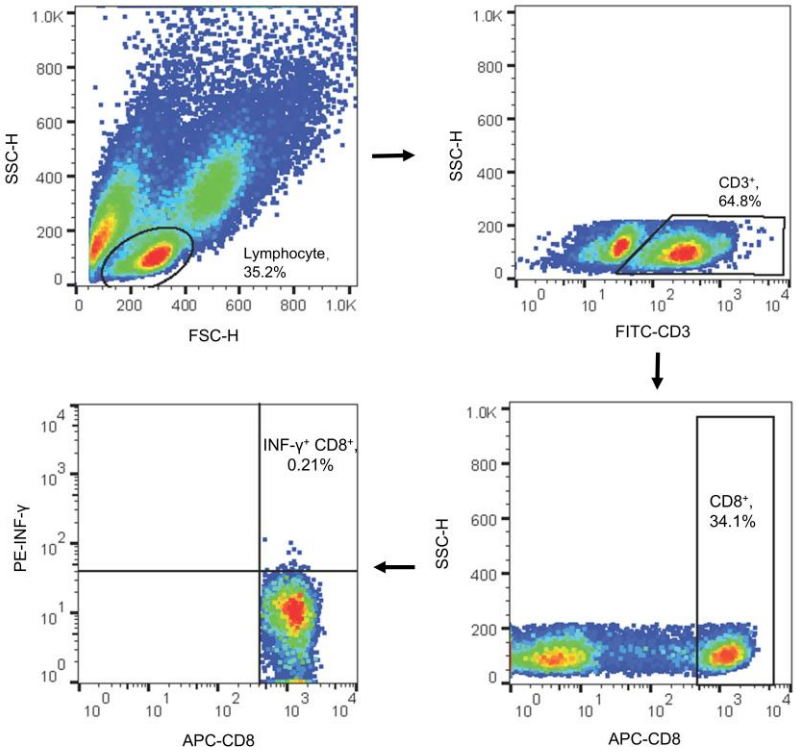

FIGURE S4 | The FACS gating strategy of intracellular IFN-γ staining in the peptide-PBMCs *ex vivo* co-cultures with PBMCs from CHB patients.

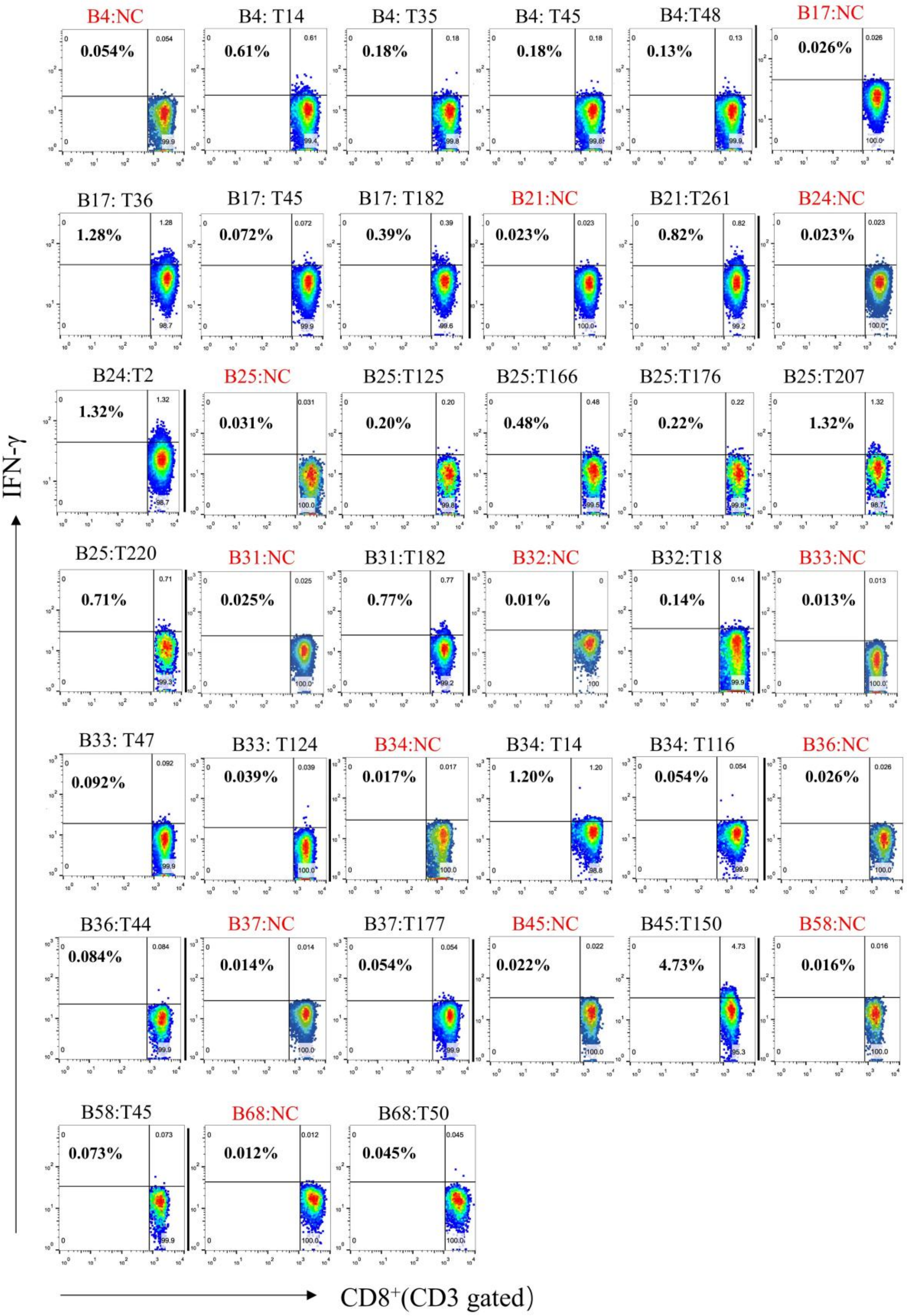

B4:NC
0.054%
B4: T14
0.61%
B4: T35
0.18%
B4: T45
0.18%
B4:T48
0.13%
B17:NC
0.026%
B17: T36
1.28%
B17: T45
0.072%
B17: T182
0.39%
B21:NC
0.023%
B21:T261
0.82%
B24:NC
0.023%
B24:T2
1.32%
B25:NC
0.031%
B25:T125
0.20%
B25:T166
0.48%
B25:T176
0.22%
B25:T207
1.32%
B25:T220
0.71%
B31:NC
0.025%
B31:T182
0.77%
B32:NC
0.01%
B32:T18
0.14%
B33:NC
0.013%
B33: T47
0.092%
B33: T124
0.039%
B34:NC
0.017%
B34: T14
1.20%
B34: T116
0.054%
B36:NC
0.026%
B36:T44
0.084%
B37:NC
0.014%
B37:T177
0.054%
B45:NC
0.022%
B45:T150
4.73%
B58:NC
0.016%
B58:T45
0.073%
B68:NC
0.012%
B68:T50
0.045%
IFN-γ
CD8+(CD3 gated)

Figure S5 (continued)

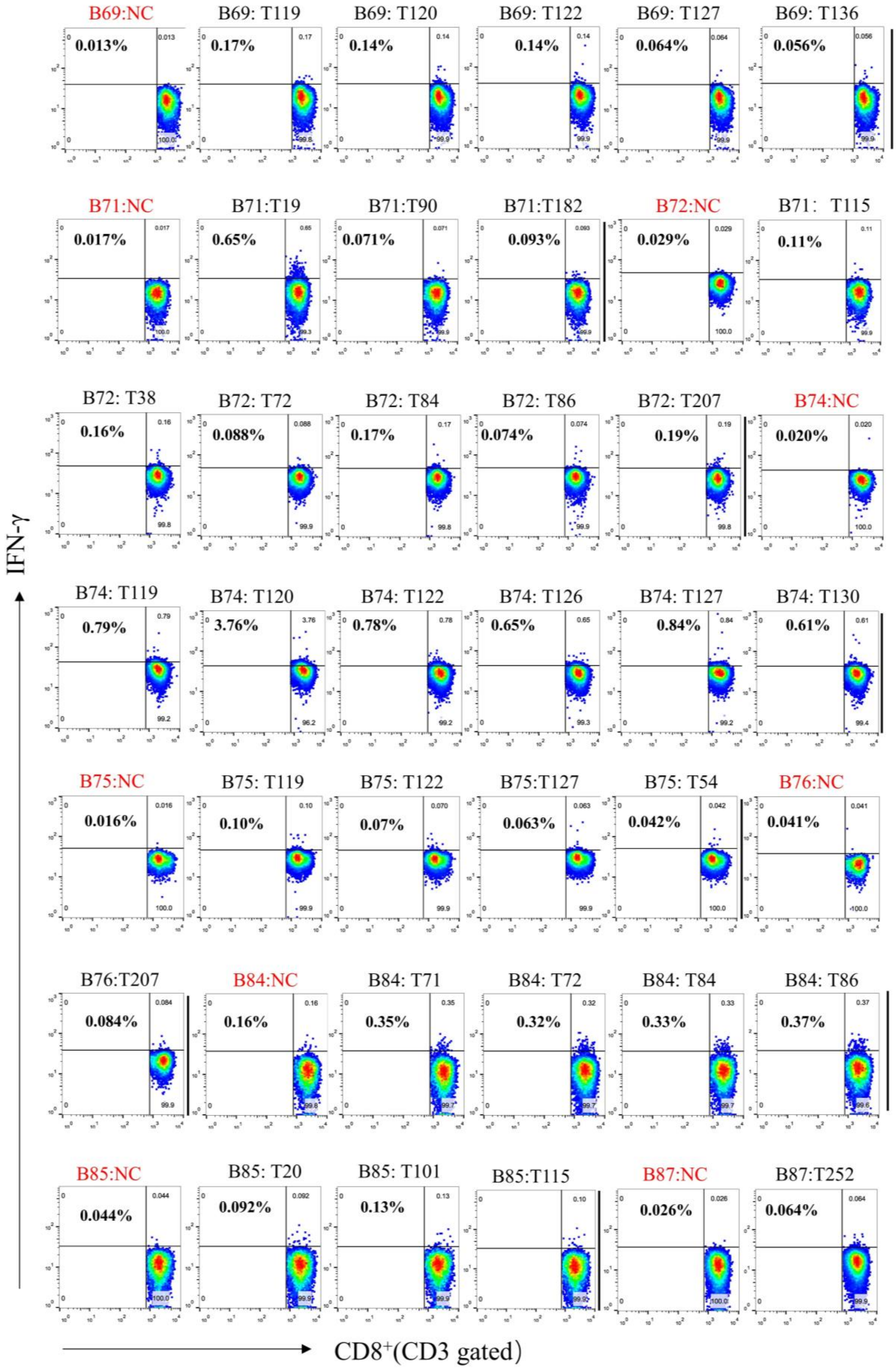

Figure S5 (continued)

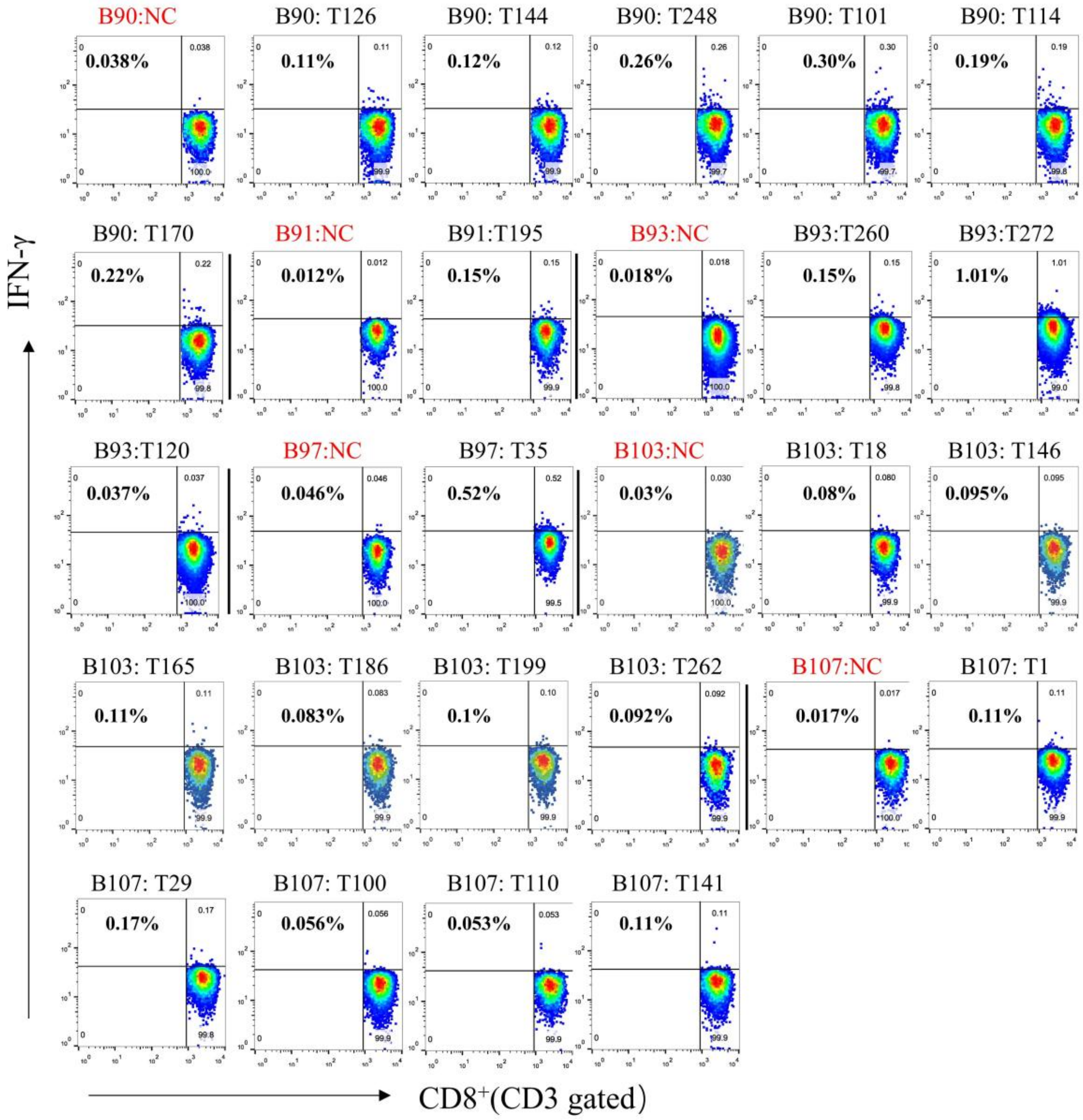

FIGURE S5 | The flow cytometric dot plots of intracellular IFN-γ staining for 57 HLA-B-restricted epitopes which inducing CD8+ T cell activation in the peptide-PBMCs *ex vivo* co-cultures with PBMCs from 29 CHB patients. After *ex vivo* cocultures of PBMCs with HLA-B-restricted candidate epitope peptides, the cells were harvested and followed by intracellular IFN-γ staining using FITC-conjugated anti-human CD3, APC-conjugated anti-human CD8 and PE-conjugated anti-human IFN-γ antibodies. After washing, the cells were harvested and analyzed by flow cytometry to determine the frequencies of IFN-γ^+ cells in $CD3^+/CD8^+$ populations. Negative control means PBMCs alone well. The serial number of each CHB patient providing PBMCs was displayed as B1 to B150. Black lines split the results of

each PBMCs sample.

Figure S5 (continued)

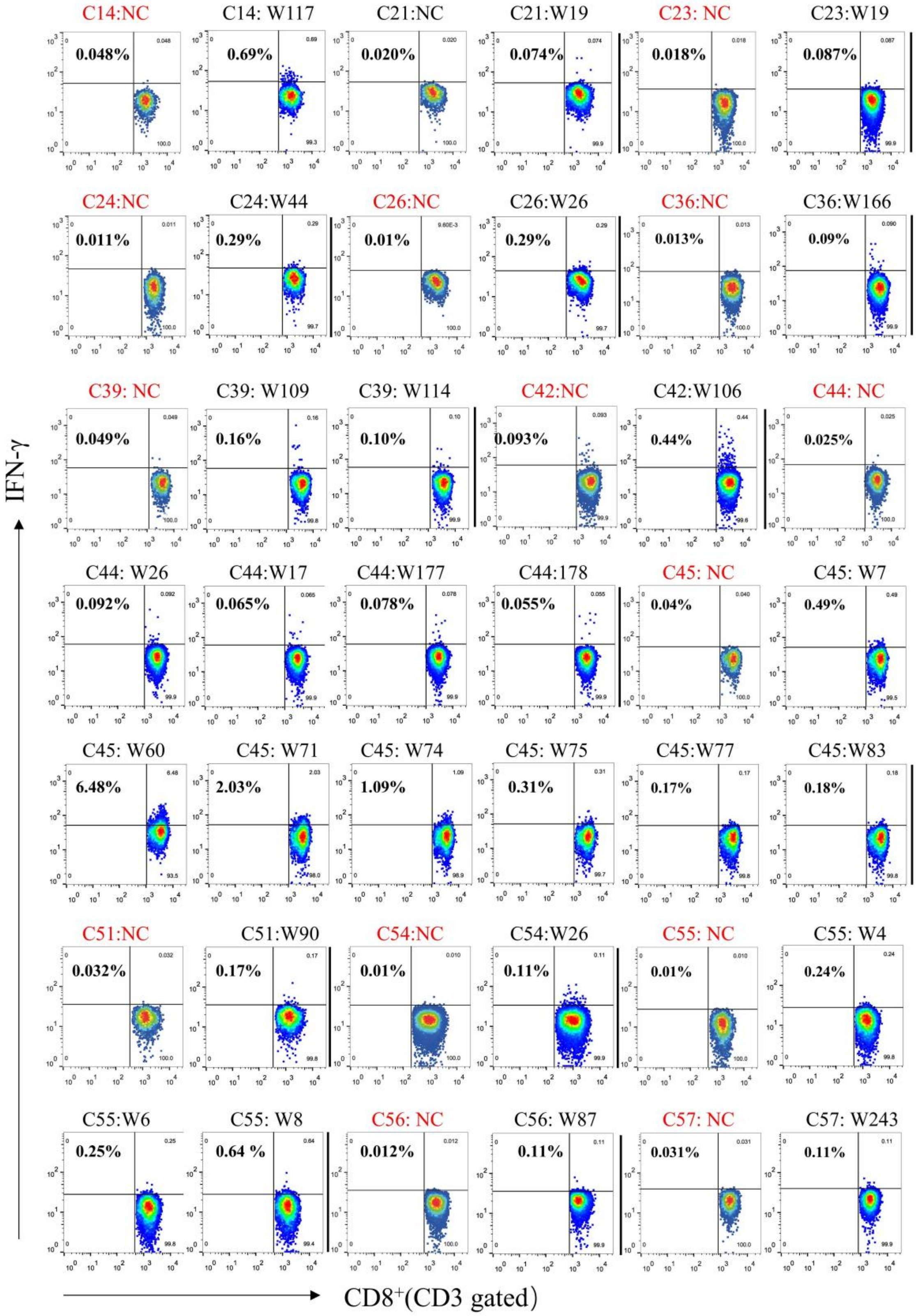

Figure S6 (continued)

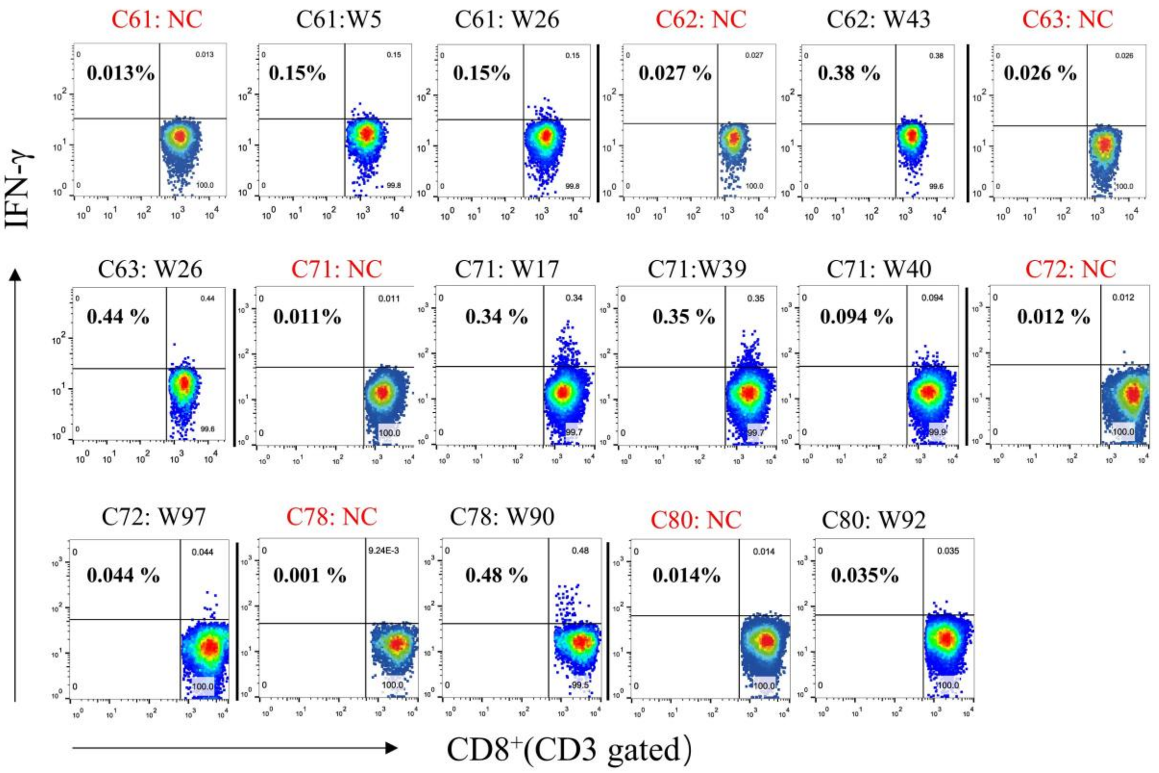

FIGURE S6 | The flow cytometric dot plots of intracellular IFN-γ staining for 32 HLA-C-restricted epitopes which inducing $CD8^+$ T cell activation in the peptide-PBMCs *ex vivo* co-cultures with PBMCs from 22 CHB patients. After *ex vivo* cocultures of PBMCs with HLA-C-restricted candidate epitope peptides, the cells were harvested and followed by intracellular IFN-γ staining using FITC-conjugated anti-human CD3, APC-conjugated anti-human CD8 and PE-conjugated anti-human IFN-γ antibodies. After washing, the cells were harvested and analyzed by flow cytometry to determine the frequencies of IFN-γ^+ cells in $CD3^+/CD8^+$ populations. Negative control means PBMCs alone well. The serial number of each CHB patient providing PBMCs was displayed as C1 to C100. Black lines split the results of each PBMCs sample.

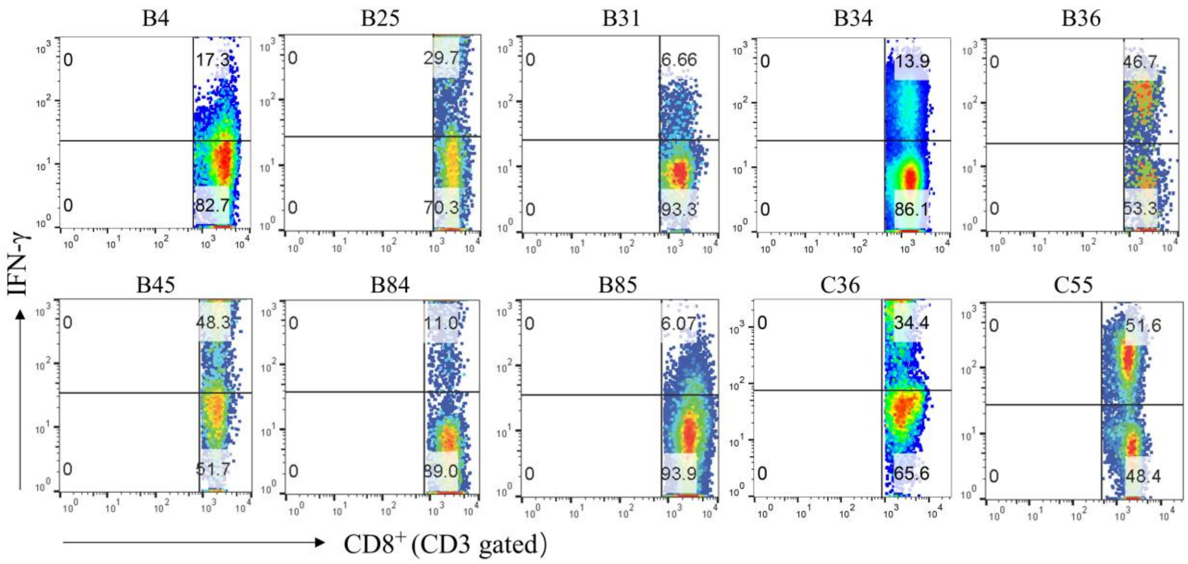

FIGURE S7 | The representative flow cytometric dot plots of intracellular IFN-γ staining for PHA stimulated positive control samples in the peptide-PBMCs *ex vivo* co-cultures with PBMCs from 10 CHB patients. The serial number above the plots was the identifier of each CHB patient providing PBMCs.

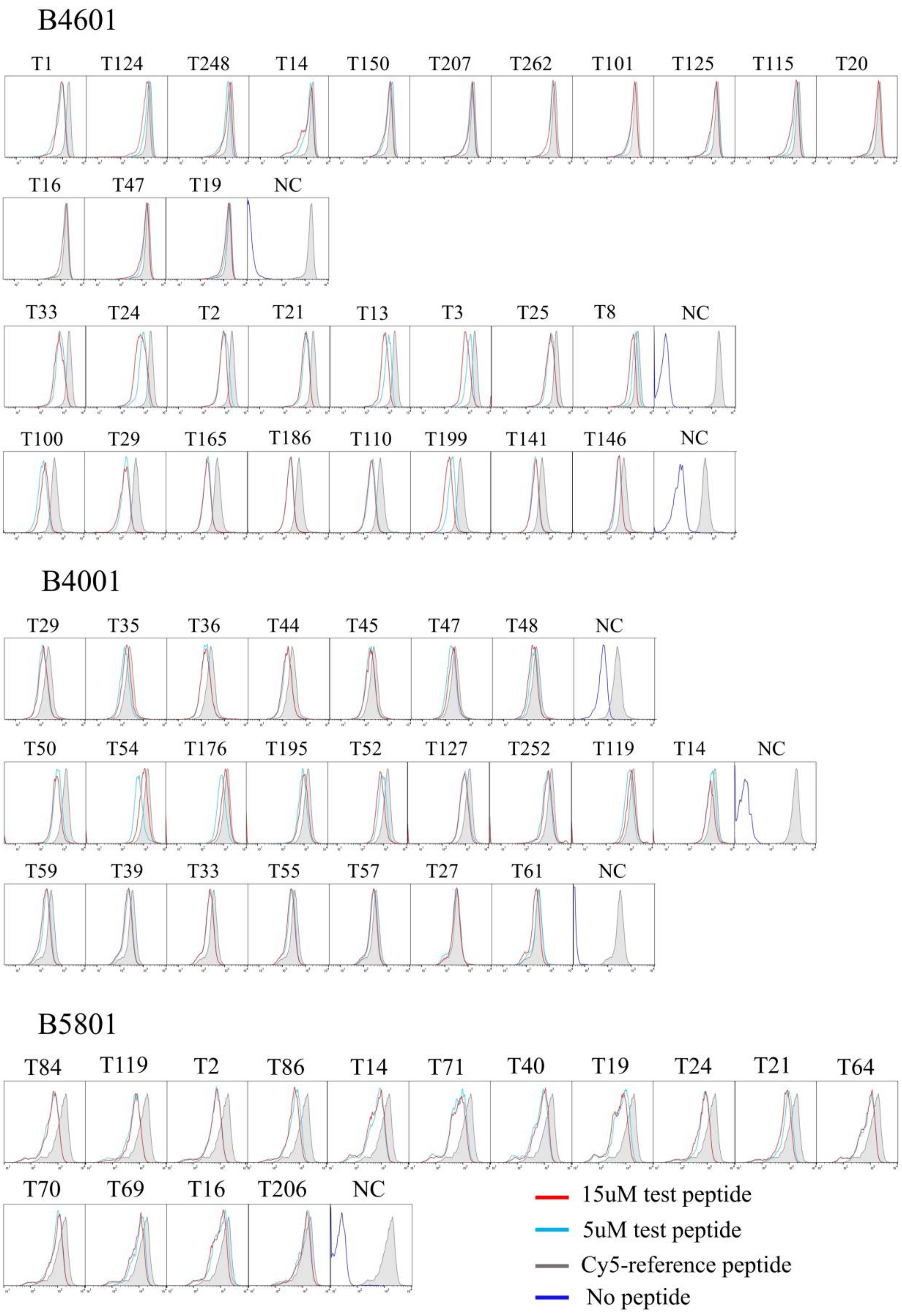
B4601
T1 T124 T248 T14 T150 T207 T262 T101 T125 T115 T20
T16 T47 T19 NC
T33 T24 T2 T21 T13 T3 T25 T8 NC
T100 T29 T165 T186 T110 T199 T141 T146 NC
B4001
T29 T35 T36 T44 T45 T47 T48 NC
T50 T54 T176 T195 T52 T127 T252 T119 T14 NC
T59 T39 T33 T55 T57 T27 T61 NC
B5801
T84 T119 T2 T86 T14 T71 T40 T19 T24 T21 T64
T70 T69 T16 T206 NC
15uM test peptide
5uM test peptide
Cy5-reference peptide
No peptide

Figure S8 (continued)

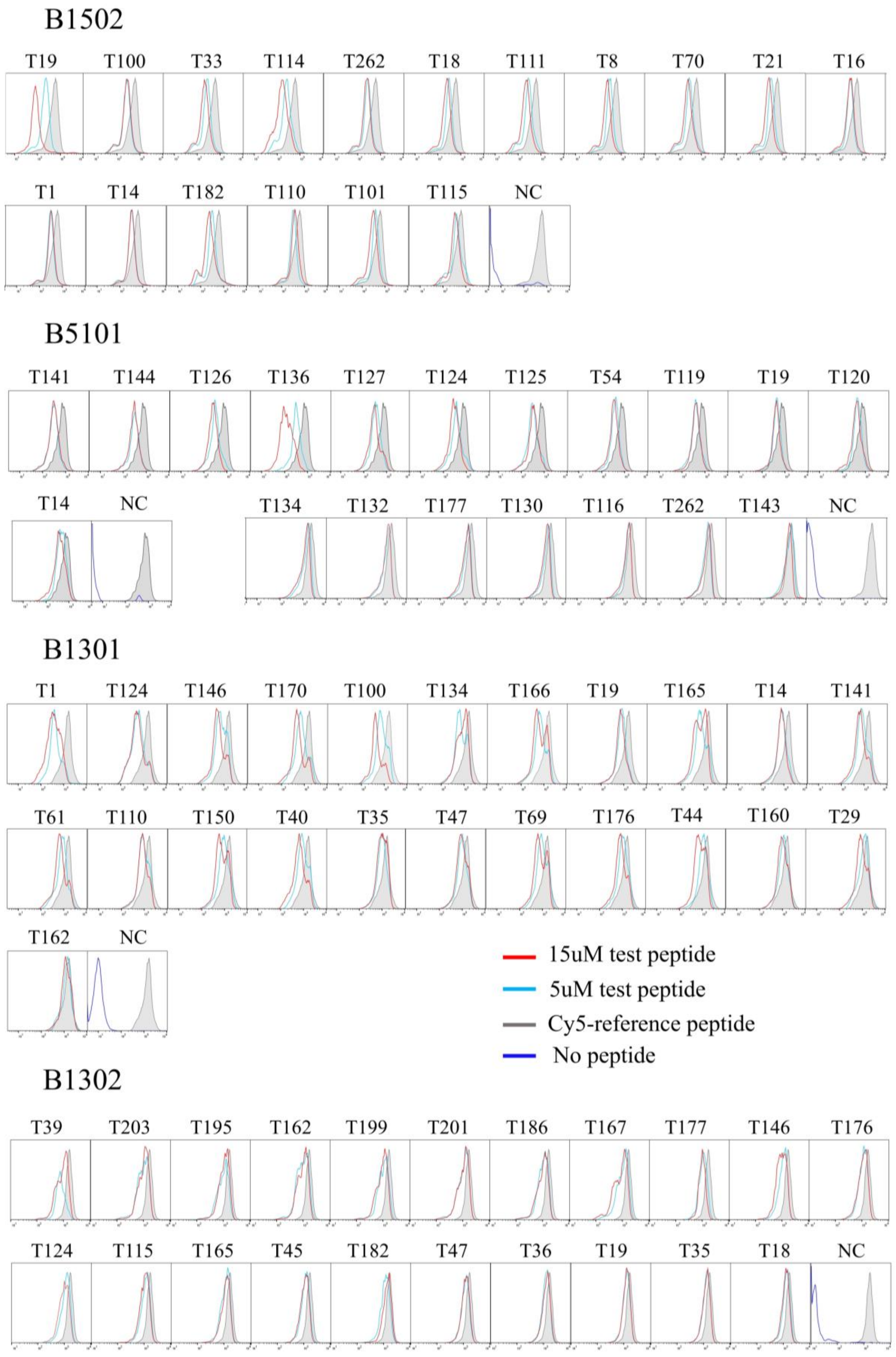

Figure S8 (continued)

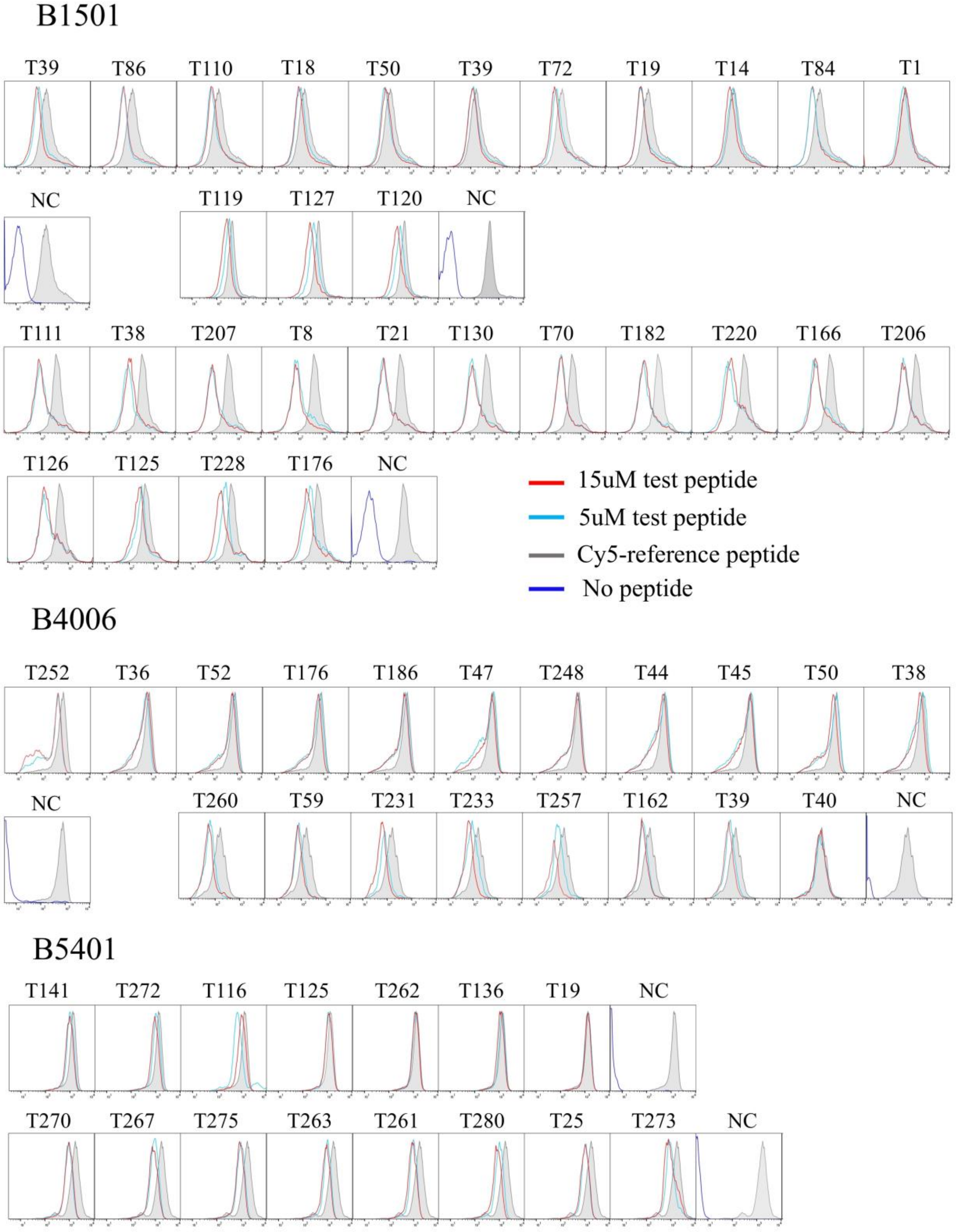

Figure S8 (continued)

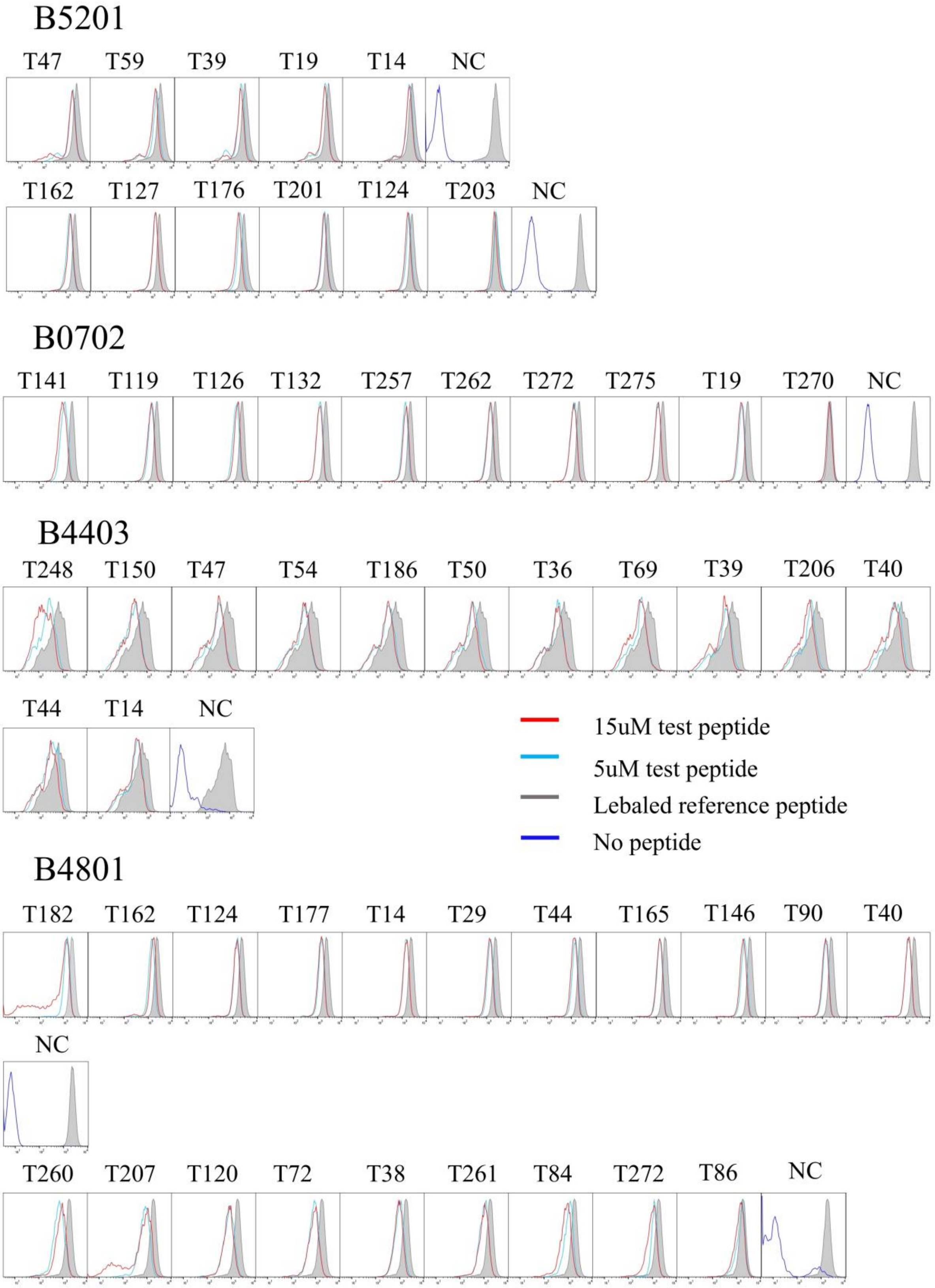

Figure S8 (continued)

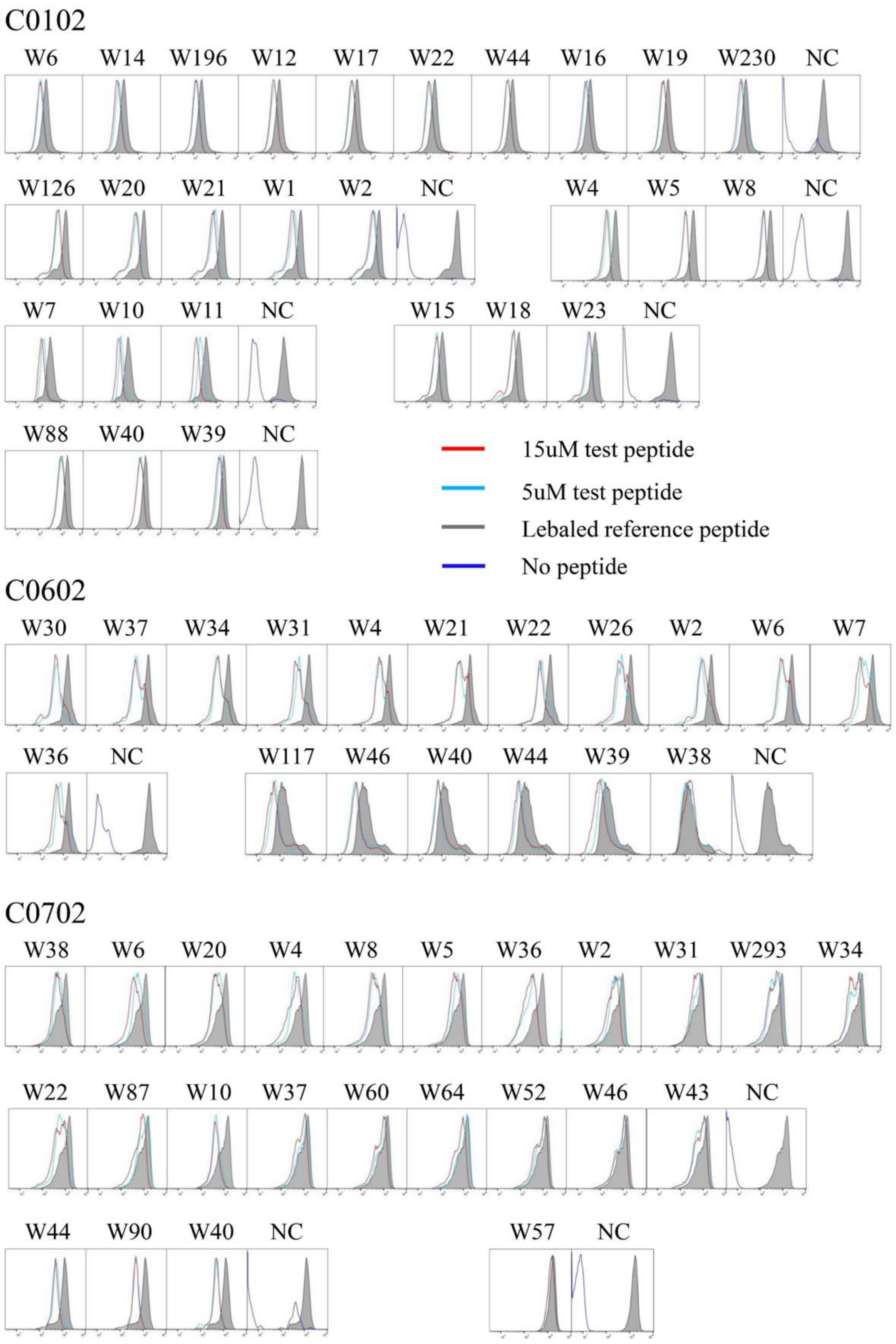

Figure S8 (continued)

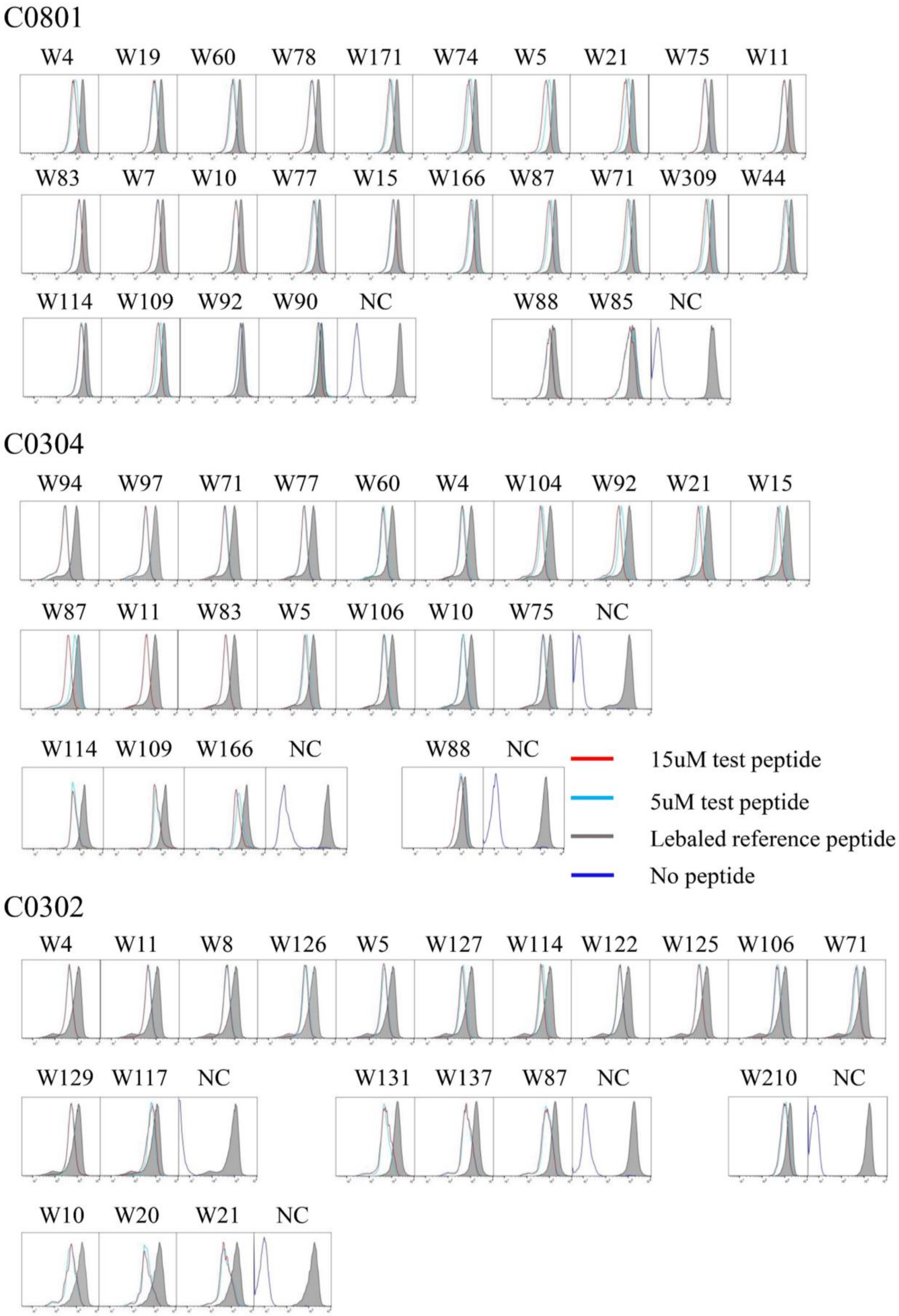

Figure S8 (continued)

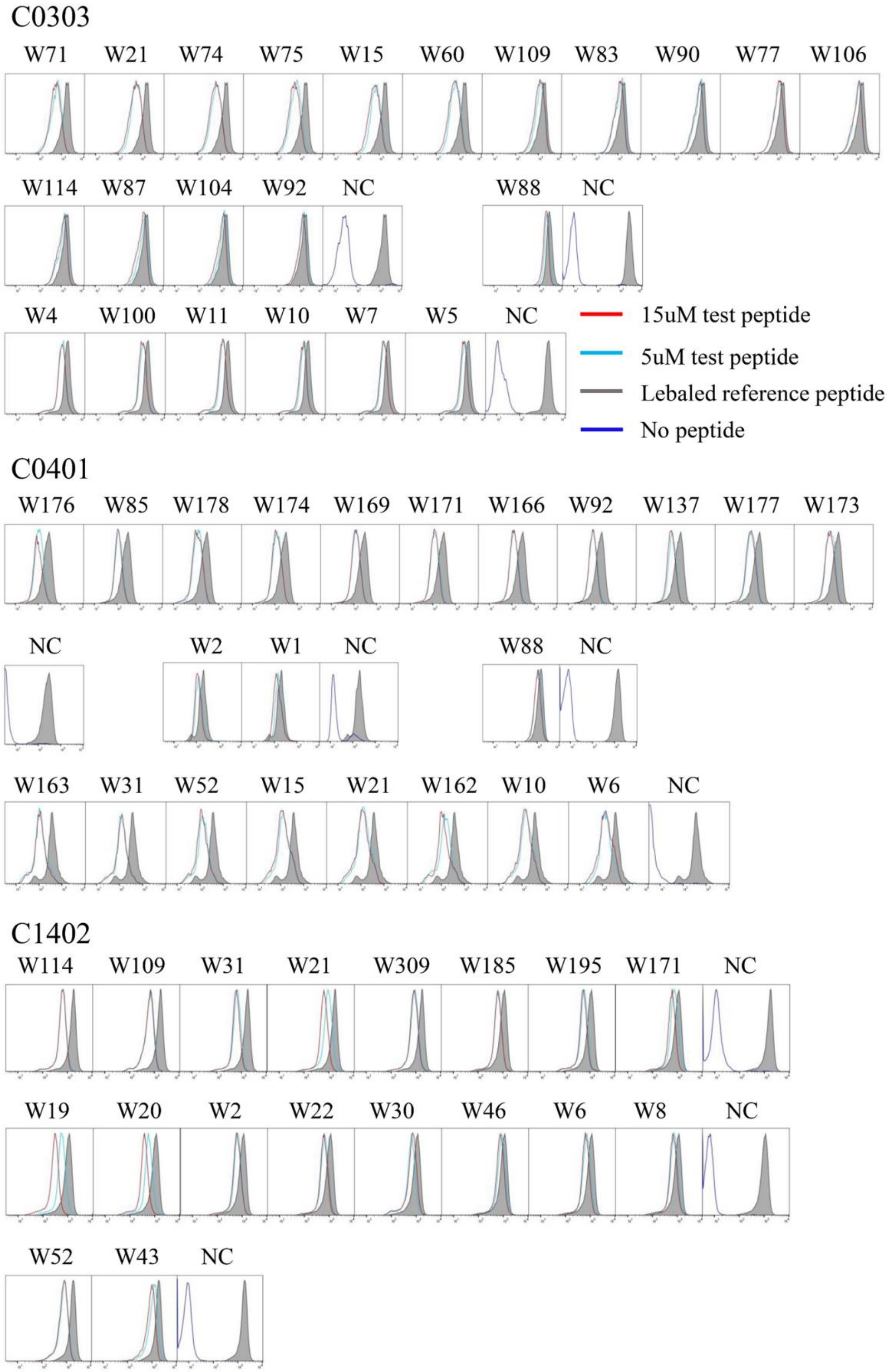

Figure S8 (continued)

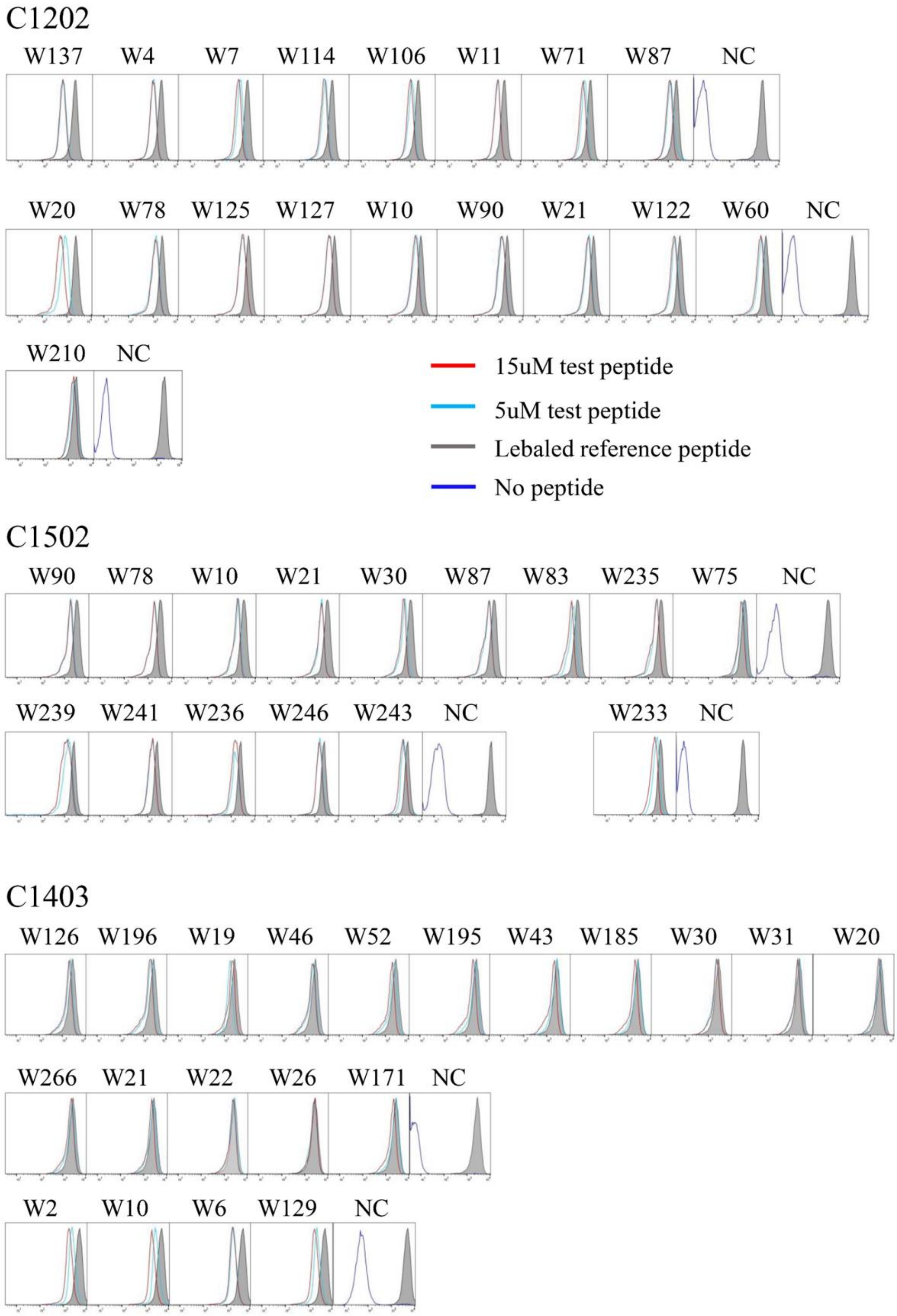

Figure S8 (continued)

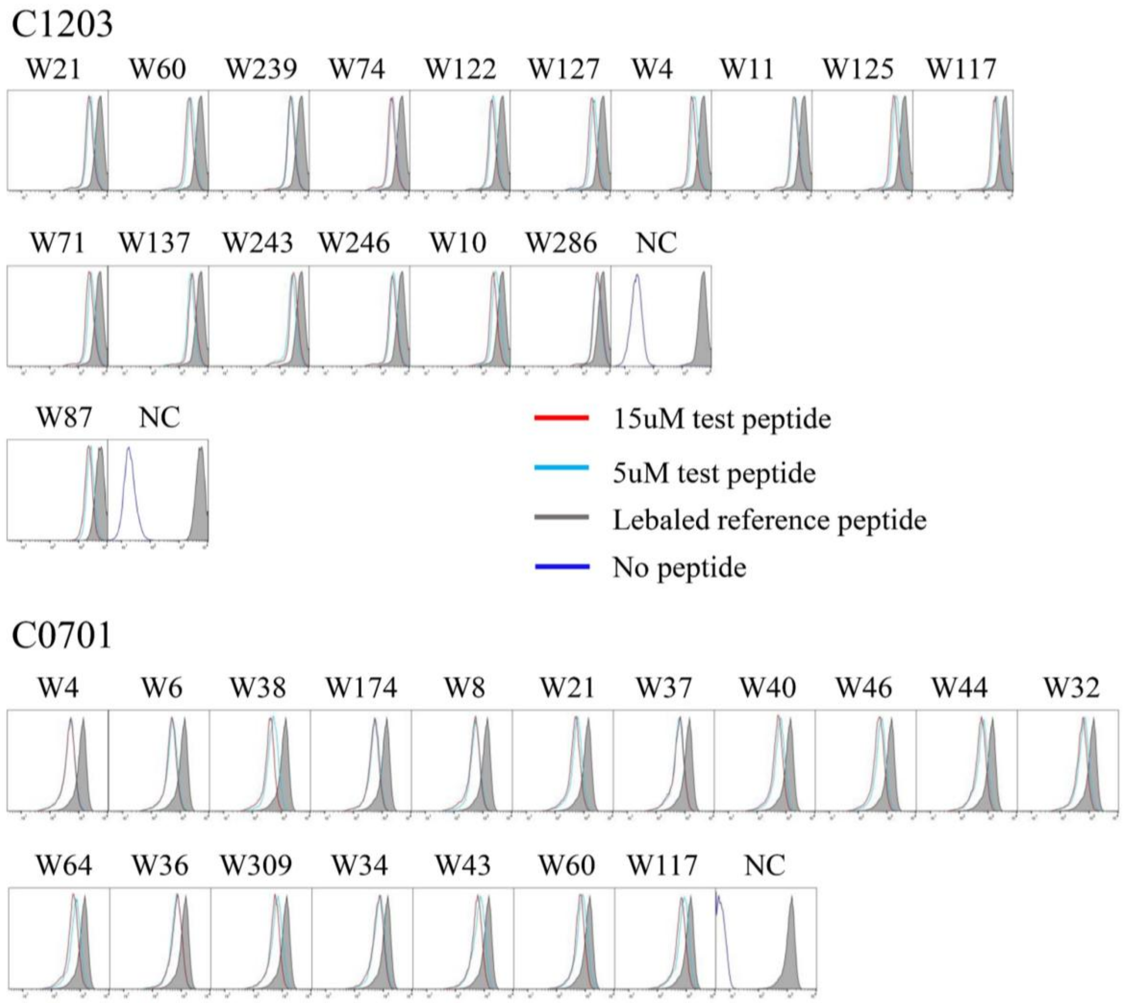

FIGURE S8 | The flow cytometric histograms of each candidate epitope peptide binding to HLA-B and C allotypes in peptide competitive binding assays. The HMy2.CIR cells stably expressing indicated HLA-B, or C molecule were incubated with Cy5-labeled reference peptide and the no-labeled candidate epitope peptide. After a defined incubation period, the unbound peptides were removed, and the relative binding affinity of the candidate epitope peptide to the HLA molecule was quantified by the declined MFI of reference peptide binding to the CIR cell surface at different concentration of candidate epitope peptide (5 μM and 15 μM). The red solid line and the blue solid line in the figure show the cell fluorescence peaks of the peptides to be tested at concentrations of 15 μM and 5 μM, respectively, while the grey filled line represents the maximum fluorescence intensity of cells in the presence of only the Cy5-reference peptide. The dark blue solid line in the NC figure represents the background fluorescence of cells in the

background well.

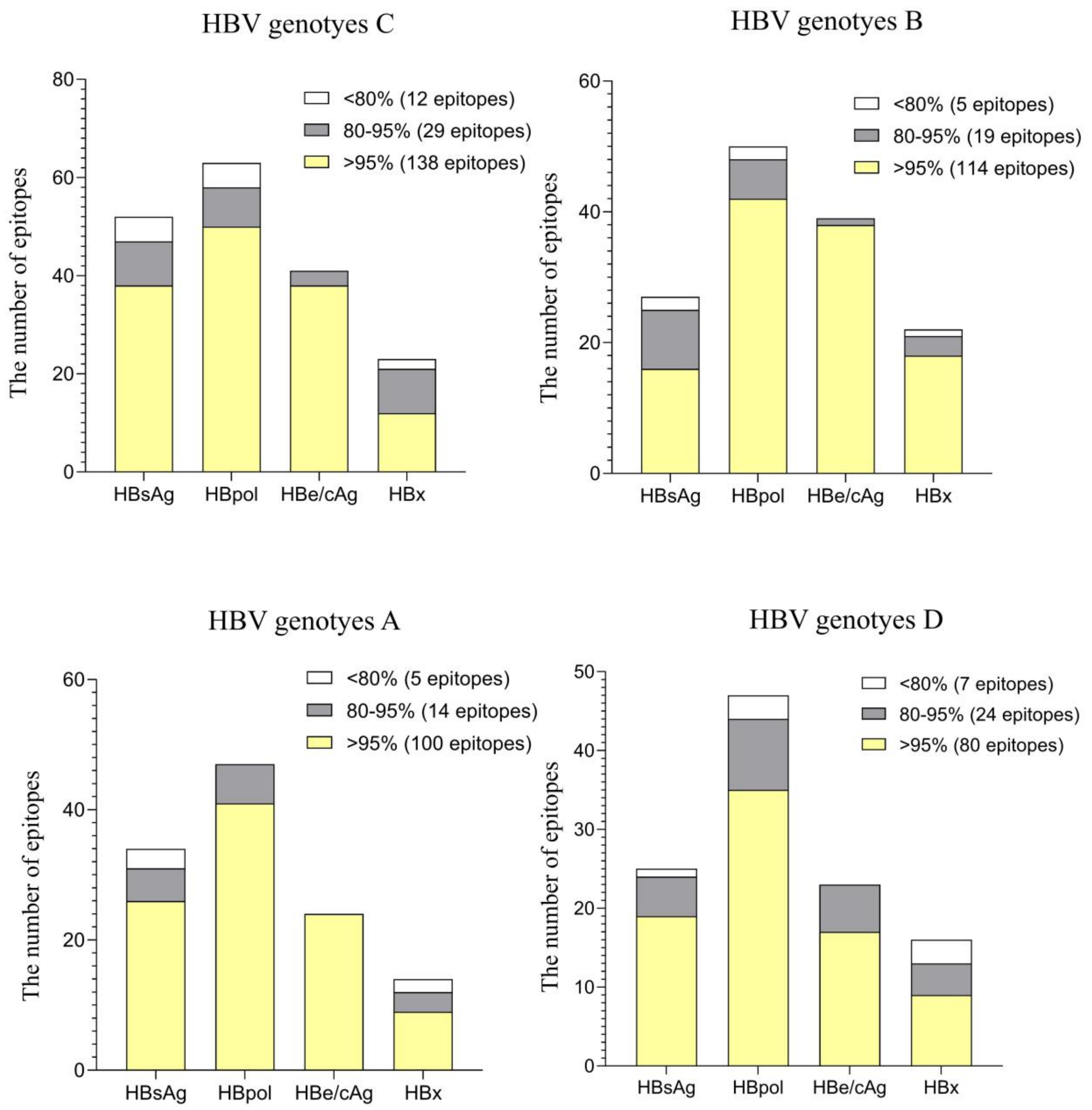

FIGURE S9 | The conservative properties of the 201 CD8$^+$ T-cell epitope peptides in the in-house ELISpot assay. For HBV genotype C, B, A and D, the numbers of epitopes with the different conservative properties were represented by different colors (yellow: ≥ 95%; grey: ≥ 80%; white: <80%).

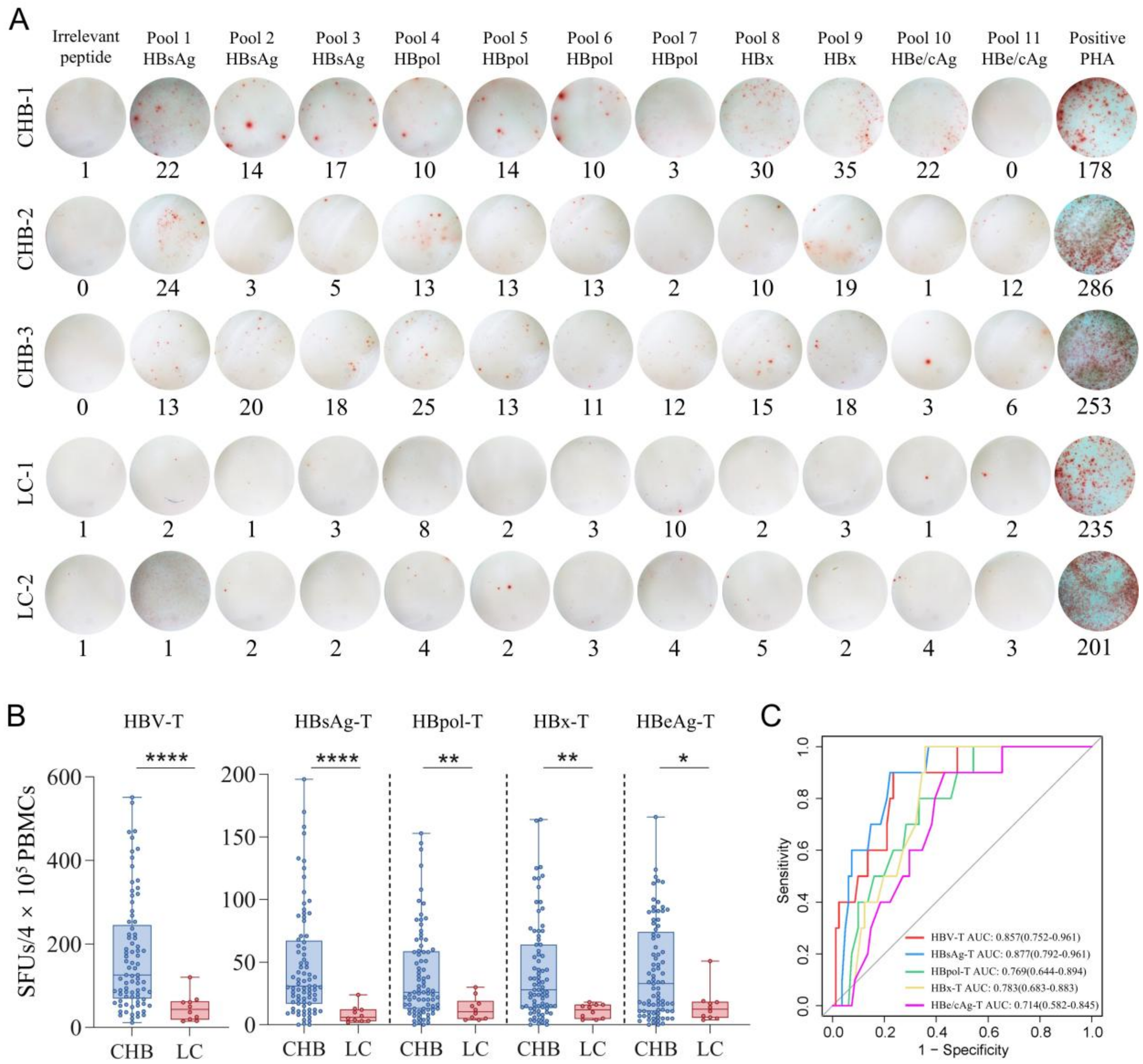

FIGURE S10 | Clinical tests of HBV-specific T cell reactivity using the improved ELISpot assay. (A) Dot plots of IFN-γ ELISpot of 3 CHB patients and 2 HBV-infected LC patients. (B) Comparison of HBV-specific-T cell reactivity between CHB and LC patients, including total HBV-specific T cells and the deconvolution of HBV-specific T cells from total antigens into the indicated HBV proteins (HBsAg, HBpol, HBe/cAg, HBx). (C) ROC curve analyses of HBV-specific T cells (SFUs/4×10^5 PBMCs) and specific T cells reactive to each HBV protein (HBsAg, HBpol, HBe/cAg, HBx) were performed to discriminate the CHB and HBV-infected LC patients. *p < 0.05; **p < 0.01; ****p < 0.0001; CHB, chronic hepatitis B; LC, liver cirrhosis.

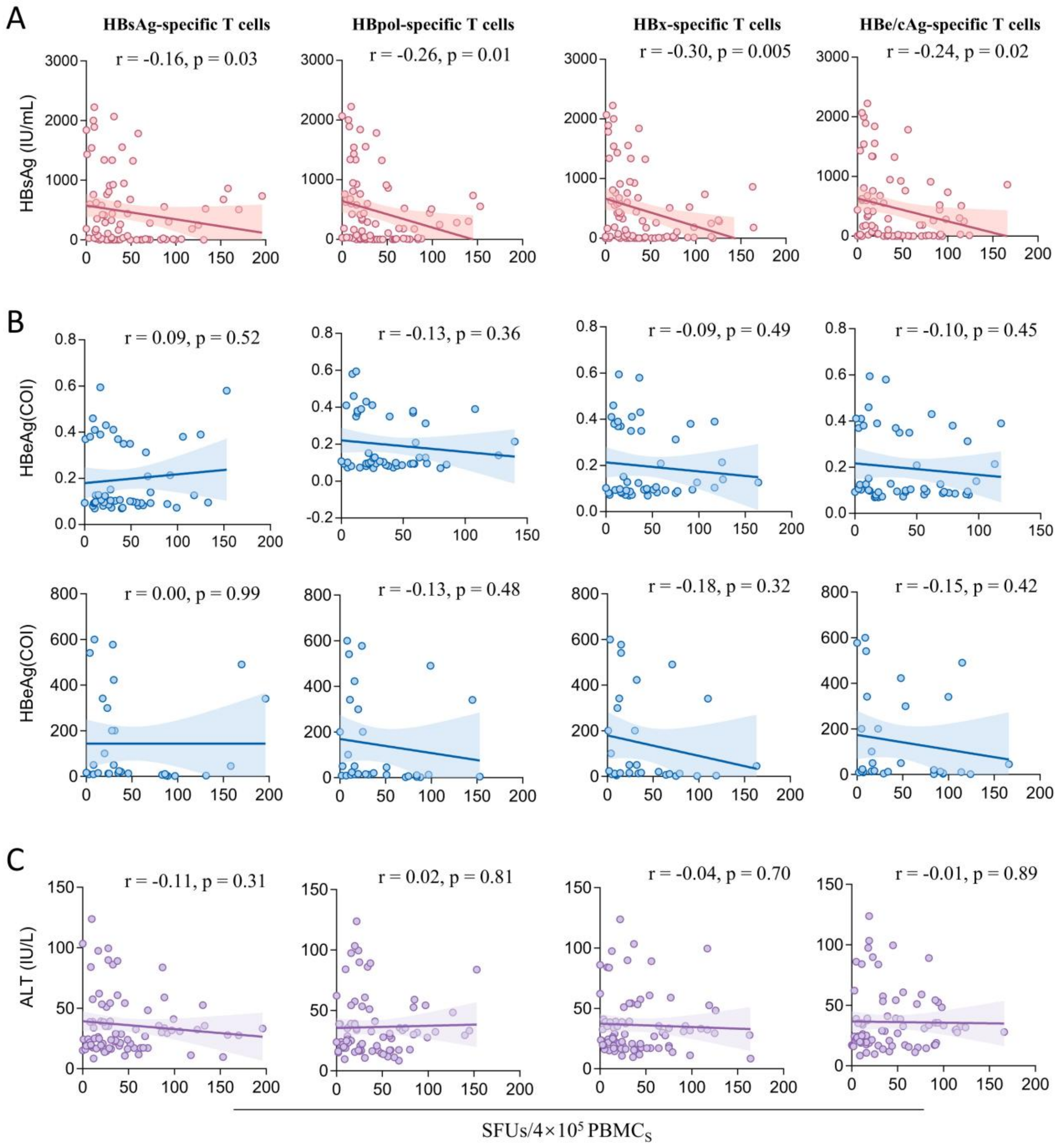

FIGURE S11 | Correlation between the specific T cells reactive to each HBV protein and sero-virological parameters in CHB patients. Spearman correlation tests between HBsAg-, HBpol-, HBx-, or HBeAg-specific T cells and HBsAg level **(A)**, HBeAg level in either the HBeAg-negative group (upper) or the HBeAg-positive group (lower) **(B)**, or ALT level **(C)** were performed.
